# Giant Armadillo Optimization: A New Bio-Inspired Metaheuristic Algorithm for Solving Optimization Problems

**DOI:** 10.3390/biomimetics8080619

**Published:** 2023-12-17

**Authors:** Omar Alsayyed, Tareq Hamadneh, Hassan Al-Tarawneh, Mohammad Alqudah, Saikat Gochhait, Irina Leonova, Om Parkash Malik, Mohammad Dehghani

**Affiliations:** 1 Department of Mathematics, Faculty of Science, The Hashemite University, P.O. Box 330127, Zarqa 13133, Jordan; omarm_re@hu.edu.jo; 2 Department of Matematics, Al Zaytoonah University of Jordan, Amman 11733, Jordan; t.hamadneh@zuj.edu.jo; 3 Department of Data Sciences and Artificial Intelligence, Al-Ahliyya Amman University, Amman 11942, Jordan; h.altarawneh@ammanu.edu.jo; 4 Department of Basic Sciences, German Jordanian University, Amman 11180, Jordan; mohammad.qudah@gju.edu.jo; 5 Symbiosis Institute of Digital and Telecom Management, Constituent of Symbiosis International Deemed University, Pune 412115, India; saikat.gochhait@sidtm.edu.in; 6 Neuroscience Research Institute, Samara State Medical University, 89 Chapaevskaya str., 443001 Samara, Russia; irina.leonova@unn.ru; 7 Faculty of Social Sciences, Lobachevsky University, 603950 Nizhny Novgorod, Russia; 8 Department of Electrical and Software Engineering, University of Calgary, Calgary, AB T2N 1N4, Canada; maliko@ucalgary.ca; 9 Department of Electrical and Electronics Engineering, Shiraz University of Technology, Shiraz 7155713876, Iran

**Keywords:** optimization, bio-inspired, metaheuristic, giant armadillo, exploration, exploitation

## Abstract

In this paper, a new bio-inspired metaheuristic algorithm called Giant Armadillo Optimization (GAO) is introduced, which imitates the natural behavior of giant armadillo in the wild. The fundamental inspiration in the design of GAO is derived from the hunting strategy of giant armadillos in moving towards prey positions and digging termite mounds. The theory of GAO is expressed and mathematically modeled in two phases: (i) exploration based on simulating the movement of giant armadillos towards termite mounds, and (ii) exploitation based on simulating giant armadillos’ digging skills in order to prey on and rip open termite mounds. The performance of GAO in handling optimization tasks is evaluated in order to solve the CEC 2017 test suite for problem dimensions equal to 10, 30, 50, and 100. The optimization results show that GAO is able to achieve effective solutions for optimization problems by benefiting from its high abilities in exploration, exploitation, and balancing them during the search process. The quality of the results obtained from GAO is compared with the performance of twelve well-known metaheuristic algorithms. The simulation results show that GAO presents superior performance compared to competitor algorithms by providing better results for most of the benchmark functions. The statistical analysis of the Wilcoxon rank sum test confirms that GAO has a significant statistical superiority over competitor algorithms. The implementation of GAO on the CEC 2011 test suite and four engineering design problems show that the proposed approach has effective performance in dealing with real-world applications.

## 1. Introduction

There are many problems in mathematics, science, and real-world applications that have more than one feasible solution. These types of problems are known as optimization problems, and the process of obtaining the best feasible solution among all these existing solutions is called optimization [1]. Each optimization problem is mathematically modeled using three main parts: decision variables, problem constraints, and an objective function. The goal in optimization is to allocate appropriate values for decision variables so that the objective function is optimized by respecting the constraints of the problem [2]. There are numerous optimization problems in science, mathematics, engineering, technology, industry, and real-world applications that need to be solved using optimization techniques. Problem-solving techniques for solving optimization problems are classified into two classes: deterministic and stochastic approaches [3]. Deterministic approaches in two categories, gradient-based and non-gradient-based, are effective in solving linear, convex, continuous, differentiable, and low-dimensional problems [4]. However, as optimization problems become more complex, especially as the problem dimensions increase, deterministic approaches stop getting stuck in local optima [5]. This is despite the fact that many practical optimization problems are non-linear, non-convex, non-differentiable, non-continuous, and high-dimensional. The disadvantages of deterministic approaches in order to solve practical optimization problems in science have led to researchers’ efforts in designing stochastic approaches [6].

Metaheuristic algorithms are among the most efficient and well-known stochastic approaches that have been used to deal with numerous optimization problems. These algorithms are able to provide suitable solutions for optimization problems based on random search in the problem-solving space and benefit from random operators and trial-and-error processes. The optimization mechanism in metaheuristic algorithms starts with the random generation of a certain number of candidate solutions under the name of algorithm population. Then, these candidate solutions are improved during successive iterations and based on the population update steps of the algorithm. After the full implementation of the algorithm, the best candidate solution obtained is presented as a solution to the problem [7]. The nature of stochastic search results in no guarantee of definitively achieving the global optimum using metaheuristic algorithms. However, due to being close to the global optimum, the solutions obtained from metaheuristic algorithms are acceptable as pseudo-optimal [8]. The desire of researchers to achieve more effective solutions closer to the global optimum for optimization problems has led to the design of numerous metaheuristic algorithms [9]. These metaheuristic algorithms have been used to tackle optimization problems in various sciences, such as static optimization problems [10], green product design [11], feature selection [12], design for disassembly [13], image segmentation [14], and wireless sensor network applications [15].

Metaheuristic algorithms will be able to achieve effective solutions for optimization problems when they search the problem-solving space well at both global and local levels. Global search expresses the exploration power of the algorithm in the extensive search in the problem-solving space with the aim of discovering the main optimal area and preventing the algorithm from getting stuck in local optima. Local search represents the exploitation power of the algorithm in the exact search near the promising areas of the problem-solving space and the discovered solutions. In addition to exploration and exploitation abilities, what leads to the success of a metaheuristic algorithm in providing a suitable solution for an optimization problem is their balancing during the search process in the problem-solving space [16].

The main research question is: according to the many metaheuristic algorithms designed so far, is there still a need to introduce newer metaheuristic algorithms in science or not? In response to this question, the No Free Lunch (NFL) [17] theorem explains that the successful performance of a metaheuristic algorithm in solving a set of optimization problems is no guarantee for the similar performance of that algorithm in solving other optimization problems. In fact, an algorithm may even converge to the global optimum in solving an optimization problem but fail in solving another problem by getting stuck in the local optimum. Therefore, there is no assumption about the failure or success of implementing a metaheuristic algorithm on an optimization problem. The NFL theorem explains that in no way can it be claimed that a unique metaheuristic algorithm is the best optimizer for all optimization problems. The NFL theorem, by keeping active the studies of metaheuristic algorithms, motivates researchers to be able to achieve more effective solutions for optimization problems by designing newer algorithms.

The innovation and novelty of this paper is the introduction of a new metaheuristic algorithm called Giant Armadillo Optimization (GAO) to solve optimization problems in various sciences. The main contributions of this study are as follows:

- GAO is designed based on simulating the natural behavior of giant armadillos in the wild.
- The fundamental inspiration for GAO is taken from the strategy of giant armadillos when attacking termite mounds.
- The GAO theory has been described and mathematically modeled in two phases: (i) exploration based on simulating the movement of giant armadillos towards termite mounds, and (ii) exploitation based on simulating giant armadillos’ digging skills in order to prey on and rip open termite mounds.
- The performance of GAO is evaluated on the CEC 2017 test suite for problem dimensions of 10, 30, 50, and 100.
- The performance of GAO in handling real-world applications is evaluated in handling twenty-two constrained optimization problems from the CEC 2011 test suite and four engineering design problems.
- The results obtained from GAO are compared with the performance of twelve well-known metaheuristic algorithms.

The proposed GAO approach has several advantages for global optimization problems. The first advantage of GAO is that there is no control parameter in the design of this algorithm, and therefore there is no need to control the parameters in any way. The second advantage of GAO is its high effectiveness in dealing with a variety of optimization problems in various sciences as well as complex, high-dimensional problems. The third advantage of the proposed GAO method is that it shows its great ability to balance exploration and exploitation in the search process, which allows it high-speed convergence to provide suitable values for decision variables in optimization tasks, especially in complex problems. The fourth advantage of the proposed GAO is its powerful performance in handling real-world optimization applications.

The structure of this paper is as follows: A literature review is presented in Section 2. Then, the proposed Giant Armadillo Optimization (GAO) is introduced and modeled in Section 3. Simulation studies and results are presented in Section 4. The effectiveness of GAO in solving real-world applications is investigated in Section 5. Conclusions and suggestions for future research are provided in Section 6.

## 2. Literature Review

Metaheuristic algorithms have been developed with inspiration from various natural phenomena, the behaviors of living organisms in the wild, genetic, biological, and physics sciences, game rules, human interactions, and other evolutionary phenomena. Metaheuristic algorithms are classified into five groups based on the main idea in design: swarm-based, evolutionary-based, physics-based, human-based, and game-based approaches.

Swarm-based metaheuristic algorithms are inspired by the lifestyles of animals, birds, insects, aquatics, reptiles, and other living creatures in the wild. The most well-known algorithms in this group are: Particle Swarm Optimization (PSO) [18], Ant Colony Optimization (ACO) [19], Artificial Bee Colony (ABC) [20], and Firefly Algorithm (FA) [21]. PSO is inspired by the group movement of flocks of birds and fish towards food sources. ACO is inspired by the ability of ants to discover the optimal communication path between the colony and the food source. ABC is inspired by the activities of colony bees searching for food sources. FA is inspired by optical communication between fireflies. The Grey Wolf Optimizer (GWO) is a swarm-based metaheuristic algorithm that is inspired by the hierarchical leadership structure and social behavior of gray wolves during hunting [22]. Green Anaconda Optimization (GAO) is inspired by the ability of male green anacondas to detect the position of females during the mating season and the hunting strategy of green anacondas [23]. Among the natural behaviors of living organisms in the wild, foraging, hunting, digging, migration, and chasing are much more prominent and have been employed in the design of algorithms such as: Honey Badger Algorithm (HBA) [24], African Vultures Optimization Algorithm (AVOA), Whale Optimization Algorithm (WOA) [25], Orca Predation Algorithm (OPA) [26], Reptile Search Algorithm (RSA) [27], Kookaburra Optimization Algorithm (KOA) [28], Mantis Search Algorithm (MSA) [29], Liver Cancer Algorithm (LCA) [30], Marine Predator Algorithm (MPA) [31], Tunicate Swarm Algorithm (TSA) [32], White Shark Optimizer (WSO) [33], and Golden Jackal Optimization (GJO) [34].

Evolutionary-based metaheuristic algorithms are designed with inspiration from genetic and biological sciences, concepts of natural selection, survival of the fittest, Darwin’s theory of evolution, and evolutionary operators. Genetic Algorithm (GA) [35] and Differential Evolution (DE) [36] are the most famous algorithms of this group, which are developed inspired by the reproduction process, genetic and biological concepts, and evolutionary-random operators of crossover, selection, and mutation. Artificial Immune Systems (AISs) are inspired by the mechanisms of the human body’s immune system against microbes and diseases [37]. Some other evolutionary-based metaheuristic algorithms are: Genetic programming (GP) [38], Cultural Algorithm (CA) [39], and Evolution Strategy (ES) [40].

Physics-based metaheuristic algorithms are designed with inspiration from the phenomena, forces, transformations, laws, and concepts of physics. Simulated Annealing (SA) is one of the most widely used algorithms of this group, which is inspired by the annealing process of metals, in which metals are first melted under heat, then slowly cooled with the aim of achieving an ideal crystal. Physical forces and Newton’s laws of motion have been the source of design in algorithms such as the Momentum Search Algorithm (MSA) [41] based on momentum force, the Gravitational Search Algorithm (GSA) based on gravitational attraction force [42], and the Spring Search Algorithm (SSA) [43] based on the elastic force of the spring and Hooke’s law. Cosmological concepts have been the origin of design in algorithms such as the Multi-Verse Optimizer (MVO) [44] and the Black Hole Algorithm (BHA) [45]. Some other physics-based metaheuristic algorithms are: Archimedes Optimization Algorithm (AOA) [46], Water Cycle Algorithm (WCA) [47], Artificial Chemical Process (ACP) [48], Chemotherapy Science Algorithm (CSA) [49], Nuclear Reaction Optimization (NRO) [50], Henry Gas Optimization (HGO) [51], Electro-Magnetism Optimization (EMO) [52], Lichtenberg Algorithm (LA) [53], Thermal Exchange Optimization (TEO) [54], and Equilibrium Optimizer (EO) [55].

Human-based metaheuristic algorithms are designed with inspiration from thoughts, choices, decisions, communication, interactions, and other human activities in individual and social life. Teaching-Learning-Based Optimization (TLBO) is one of the most famous human-based metaheuristic algorithms, which is introduced with the inspiration of educational communication in the classroom environment and the exchange of knowledge between teachers and students and students with each other [56]. The Mother Optimization Algorithm (MOA) is proposed based on the modeling of Eshrat’s care of her children [57]. Doctor and Patient Optimization (DPO) is introduced based on modeling the process of treating patients by doctors [58]. Sewing Training-Based Optimization (STBO) is proposed with the inspiration of teaching sewing skills by the instructor to students in sewing schools [59]. Ali Baba and the Forty Thieves (AFT) is presented based on modeling the strategies of forty thieves in the search for Ali Baba [60]. Some other human-based metaheuristic algorithms are: Election-Based Optimization Algorithm (EBOA) [61], Coronavirus Herd Immunity Optimizer (CHIO) [62], Group Teaching Optimization Algorithm (GTOA) [63], Ebola Optimization Search Algorithm (ESOA) [64], Driving Training-Based Optimization (DTBO) [5], Gaining Sharing Knowledge-Based Algorithm (GSK) [65], and War Strategy Optimization (WSO) [66].

Game-based metaheuristic algorithms are inspired by the rules governing individual and team games and the strategies of players, coaches, referees, and other influential people in these games. Darts Game Optimizer (DGO) is one of the most well-known game-based metaheuristic algorithms, whose design is inspired by the strategy and skill of players in throwing darts and collecting points [67]. Hide Object Game Optimizer (HOGO) is proposed based on the simulation of players’ strategies for finding the hidden object on the playing field [68]. The Orientation Search Algorithm (OSA) is designed based on modeling the players’ position changes on the playing field based on the referee’s commands [69]. Some other game-based metaheuristic algorithms are: Ring toss game-based optimization (RTGBO) [70], Football Game Based Optimization (FGBO) [71], Archery Algorithm (AA) [6], Golf Optimization Algorithm (GOA) [72], and Volleyball Premier League (VPL) [73].

Some other recently proposed metaheuristic algorithms are: Monarch Butterfly Optimization (MBO) [74], Slime Mould Algorithm (SMA) [75], Moth Search Algorithm (MSA) [76], Hunger Games Search (HGS) [77], Runge Kutta method (RUN) [78], Colony Predation Algorithm (CPA) [79], weighted mean of vectors (INFO) [80], Harris Hawks Optimization (HHO) [81], and Rime optimization algorithm (RIME) [82].

Based on the best knowledge obtained from the literature review, no metaheuristic algorithm inspired by the natural behavior of giant armadillos in nature has been designed so far. This is while the strategy of giant armadillos in attacking termite mounds and digging them is an intelligent process that has a special potential for designing a new optimizer. In order to address this research gap, a new bio-inspired metaheuristic algorithm is introduced in this paper based on the mathematical modeling of the strategy of giant armadillos in attacking and hunting in termite mounds, which is discussed in the next section.

## 3. Giant Armadillo Optimization

In this section, the source of inspiration in the design of the proposed Giant Armadillo Optimization (GAO) approach is stated, and then it is mathematically modeled in order to use it in optimization applications.

### 3.1. Inspiration for GAO

The giant armadillo (Priodontes maximus) is the largest living species of armadillo in danger of extinction and lives in South America, ranging as far south as northern Argentina [83]. Termites and ants are the main diet of giant armadillos. However, this animal also feeds on plants, larvae, worms, and larger creatures, such as snakes and spiders. In order to feed on termites, giant armadilloes attack termite mounds and then use their digging power to prey on and rip open termite mounds.

**Figure 1:**
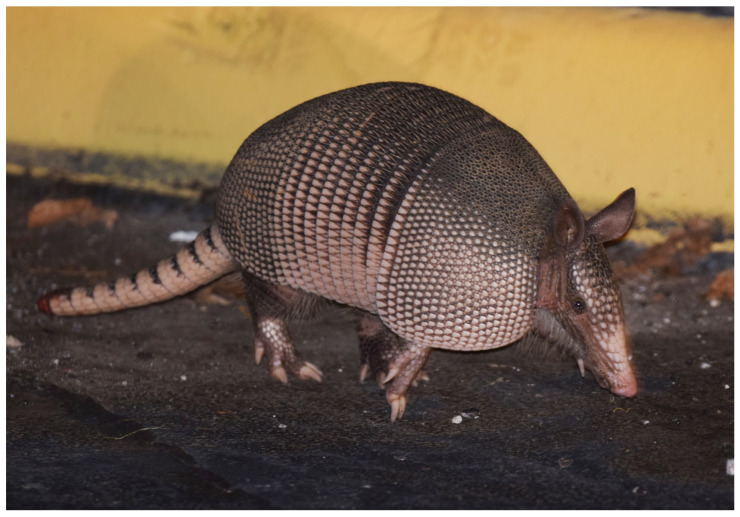
Giant armadillo taken from: free media Wikimedia Commons.

The giant armadillo has 3 or 4 hinged bands protecting the neck and another 11 to 13 hinged bands that protect the body [84]. Its body is dark brown with a lighter yellowish band along the sides, and its head is pale and yellowish-white. It also has very long front paws, up to 22 cm long. The tail is covered in small, rounded scales. The giant armadillo is almost entirely hairless. Giant armadillos weigh approximately 18.7–32.5 kg, although specimens weighing 54 kg and 80 kg have also been observed. Their length without including the tail is between 75 and 100 cm, and the length of their tail is about 50 cm [85]. An image of the giant armadillo is shown in Figure 1.

Among the natural behaviors of the giant armadillo, the strategy of this animal when it attacks termite mounds and then digs them with the aim of hunting and feeding on termites is much more prominent. Mathematical modeling of these two natural behaviors of giant armadillos during hunting, namely (i) attacking termite mounds and (ii) digging termite mounds in order to feed on them, has been employed in the design of the proposed GAO approach, which is discussed below.

Among the natural behaviors of giant armadillos, the hunting strategy of this animal is much more prominent. The giant armadillo hunting process has two stages: (i) moving towards termite mounds and (ii) digging in termite mounds in order to feed on termites. Mathematical modeling of these natural behaviors of the giant armadillo during hunting is employed in the design of the proposed GAO approach, which is discussed below.

### 3.2. Solution Process of the GAO

The proposed GAO approach is a biomimetics metaheuristic algorithm that mimics the natural behavior of the giant armadillo in the wild. Among the natural behaviors of the giant armadillo, the strategy of this animal in attacking termite mounds and then digging in them for feeding is employed in the GAO design. In this modeling, the wild life of the giant armadillo corresponds to the problem-solving space, and the position of each giant armadillo in the wild corresponds to the position of each GAO member in the problem-solving space as a candidate solution. The general solution process of the algorithm in GAO is explained in Algorithm 1.
**Algorithm 1:** Solution process of GAOStart.A certain number of giant armadillos are randomly initialized in the problem-solving space as a population of the algorithm, each representing a candidate solution for the problem.Based on the evaluation of each of the candidate solutions in the objective function and the comparison of the obtained values, the best GAO member is identified as the best candidate solution.In the first phase of the GAO, based on the modeling of the movement of the giant armadillo towards the termite mounds, the position of the GAO members in the problem-solving space and, as a result, the candidate solutions are updated.In the second phase of GAO, based on the modeling of the small displacements of the giant armadillo while digging in termite mounds, the position of GAO members in the problem-solving space and, as a result, candidate solutions are updated.The third and fourth steps are repeated for all GAO members.Based on the comparison of the new evaluated values for the objective function corresponding to the updated candidate solutions, the best candidate solution is identified, updated, and stored.The third to sixth steps are repeated until the last iteration of the algorithm.The best candidate solution obtained during the iterations of the algorithm is presented as the GAO solution for the given problem.End.

In the following, the solution process described for GAO is mathematically modeled in full.

### 3.3. Mathematical Modeling of GAO

In this subsection, the implementation steps of GAO are fully modeled. For this purpose, first, the initialization process of GAO has been explained and modeled. Then, the mathematical model of the process of updating candidate solutions in two phases of exploration and exploitation is presented.

#### 3.3.1. Algorithm Initialization

The proposed GAO approach is a population-based meta-heuristic algorithm that assumes that giant armadillos form its population. GAO is able to provide suitable solutions for optimization problems in an iterative process based on the search power of its members in the problem-solving space. Each GAO member, based on his position in the problem-solving space, determines the values for the decision variables of the problem. Therefore, each giant armadillo, as a member of the population, is a candidate solution to the problem that is modeled from a mathematical point of view using a vector. Giant armadillos together form the population of the algorithm, which can be modeled from a mathematical point of view using a matrix according to Equation (1). The primary position of the giant armadillos in the problem-solving space is randomly initialized at the beginning of the algorithm execution using Equation (2).

(1) $$X={\left[\begin{array}{c} X_{1}\\ \vdots \\ X_{i}\\ \vdots \\ X_{N} \end{array}\right]}_{N\times m}={\left[\begin{array}{ccccc} x_{1,1} & \cdots & x_{1,d} & \cdots & x_{1,m}\\ \vdots & \ddots & \vdots & ⋰ & \vdots \\ x_{i,1} & \cdots & x_{i,d} & \cdots & x_{i,m}\\ \vdots & ⋰ & \vdots & \ddots & \vdots \\ x_{N,1} & \cdots & x_{N,d} & \cdots & x_{N,m} \end{array}\right]}_{N\times m}$$

(2) $$x_{i,d}=lb_{d}+r\cdot \left(ub_{d}-lb_{d}\right)$$

Here, X is the GAO population matrix, $X_{i}$ is the ith GAO member (candidate solution), $x_{i,d}$ is its dth dimension in search space (decision variable), N is the number of giant armadillos, m is the number of decision variables, r is a random number in interval [0,1], $lb_{d}$, and $ub_{d}$ are the lower bound and upper bound of the dth. decision variable, respectively.

Since the position of each giant armadillo in the problem-solving space represents a candidate solution for the problem, a value for the objective function can be evaluated corresponding to each giant armadillo. According to this, the set of evaluated values for the objective function can be represented using Equation (3).

(3) $$F={\left[\begin{array}{c} F_{1}\\ \vdots \\ F_{i}\\ \vdots \\ F_{N} \end{array}\right]}_{N\times 1}={\left[\begin{array}{c} F\left(X_{1}\right)\\ \vdots \\ F\left(X_{i}\right)\\ \vdots \\ F\left(X_{N}\right) \end{array}\right]}_{N\times 1}$$

Here, F is the vector of the evaluated objective function, and $F_{i}$ is the evaluated objective function based on the ith GAO member.

The evaluated values for the objective function provide valuable information about the quality of the candidate solutions proposed by the population members. The best value obtained for the objective function corresponds to the best member (i.e., the best candidate solution), and the worst value obtained for the objective function corresponds to the worst member (i.e., the worst candidate solution). Since in each iteration, the position of the giant armadillos in the problem-solving space is updated, the best member should also be updated based on the comparison of the updated values for the objective function. At the end of the implementation of the algorithm, the position of the best member obtained during the iterations of the algorithm is presented as a solution to the problem.

In the design of the proposed GAO approach, the position of the population members in the problem-solving space is updated based on the modeling of the hunting strategy of giant armadillos in the wild. In this process, the giant armadillo first attacks the position of termite mounds, then digs in termite mounds to hunt and eat termites. According to this, in each iteration of GAO, the position of the population members is updated in two phases: (i) exploration, based on the simulation of the movement of giant armadillos towards termite mounds, and (ii) exploitation, based on the simulation of giant armadillos digging in termite mounds to feed on termites.

#### 3.3.2. Phase 1: Attack on Termite Mounds (Exploration Phase)

In the first phase of GAO, the position of the population members in the problem-solving space is updated based on the simulation of the attack of the giant armadillo towards the termite mounds during hunting. In the GAO design, it is inspired by the changing position of the giant armadillo while moving towards the termite mounds in order to update the position of the population members in the problem-solving space. Modeling this attack process leads to extensive changes in the position of the giant armadillo and, as a result, increases the exploration power of the algorithm in global search management.

In the GAO design, for each population member that represents a giant armadillo, the location of other population members that have a better objective function value is considered a termite mound. The set of candidate termite mounds for each member of the population is specified using Equation (4).

(4) $$TM_{i}=\left\{X_{k}:F_{k}<F_{i}\;\mathrm{and}\;k\neq i\right\},\;\mathrm{where}\;i=1,2,\;\ldots ,\;N\;\mathrm{and}\;k\in \left\{1,2,\;\ldots ,\;N\right\}$$

Here, $TM_{i}$ is the set of candidate termite mounds’ locations for the ith giant armadillo, $X_{k}$ is the population member with a better objective function value than the ith giant armadillo, and $F_{k}$ is its objective function value.

The giant armadillo randomly selects one of the candidate termite mounds and attacks it. Based on modeling the movement of giant armadilloes towards termite mounds, a new position is calculated for each member of the population using Equation (5). Then, this new position replaces the previous position of the corresponding member if it improves the value of the objective function according to Equation (6).

(5) $$x_{i,j}^{P1}=x_{i,j}+r_{i,j}\cdot \left(STM_{i,j}-I_{i,j}\cdot x_{i,j}\right),$$

(6) $$X_{i}=\{\begin{array}{c} X_{i}^{P1},\;\;F_{i}^{P1}\leq F_{i},\\ X_{i},\;\;else, \end{array}$$

Here, $STM_{i}$ is the selected termite mound for ith giant armadillo, $STM_{i,j}$ is its jth dimension, $X_{i}^{P1}$ is the new position calculated for the ith giant armadillo based on attacking phase of the proposed GAO, $x_{i,j}^{P1}$ is its jth dimension, $F_{i}^{P1}$ is its objective function value, $r_{i,j}$ are random numbers from the interval [0, 1], and $I_{i,j}$ are numbers which are randomly selected as 1 or 2.

#### 3.3.3. Phase 2: Digging in Termite Mounds (Exploitation Phase)

In the second phase of GAO, the position of population members in the problem-solving space is updated based on the simulation of giant armadillo digging in termite mounds to feed on termites. Modeling this giant armadillo digging process with the aim of hunting and eating termites leads to small changes in the position of the giant armadillo and, as a result, increases the exploitation power of the algorithm in local search management.

In the GAO design, based on modeling the skill of the giant armadillo to dig in termite mounds, a new position is calculated for each member of the population using Equation (7). Then, if the value of the objective function is improved, this new position replaces the previous position of the corresponding member according to Equation (8).

(7) $$x_{i,j}^{P2}=x_{i,j}+\left(1-2\;r_{i,j}\right)\cdot \frac{ub_{j}-lb_{j}}{t}\;\;$$

(8) $$X_{i}=\{\begin{array}{c} X_{i}^{P2},\;\;F_{i}^{P2}\leq F_{i}\\ X_{i},\;\;else \end{array}$$

Here, $X_{i}^{P2}$ is the new position calculated for the ith giant armadillo based on digging phase of the proposed GAO, $x_{i,j}^{P2}$ is its jth dimension, $F_{i}^{P2}$ is its objective function value, $r_{i,j}$ are random numbers from the interval [0, 1], and t is the iteration counter.

### 3.4. Repetition Process, Pseudocode, and Flowchart of GAO

**Figure 2:**
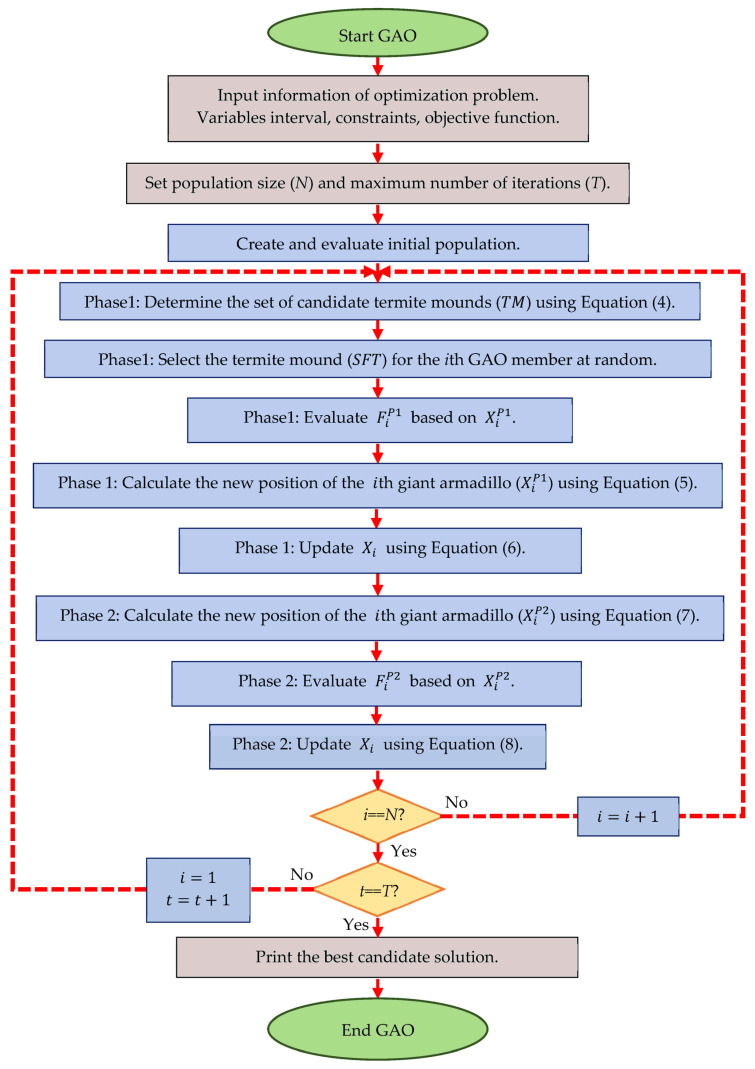
Flowchart of GAO.

After updating the position of all giant armadillos in the problem-solving space based on the attack and digging phases, the first iteration of GAO is completed. After that, the algorithm enters the next iteration, and the process of updating the position of giant armadillos in the problem-solving space continues until the last iteration of the algorithm using Equations (4)–(8). In each iteration, the position of the best GAO member is updated and stored as the best candidate solution. After the full implementation of GAO on the given problem, the best candidate solution recorded during the iterations of the algorithm is presented as the solution to the problem. The implementation steps of GAO are presented as a flowchart in Figure 2, and its pseudocode is presented in Algorithm 2. The complete set of codes is available at the following repository: https://uk.mathworks.com/matlabcentral/fileexchange/156329-giant-armadillo-optimization (accessed on 13 November 2023).
**Algorithm 2:** Pseudocode of GAOStart GAO.1.Input problem information: variables, objective function, and constraints.2.Set GAO population size (*N*) and iterations (*T*).3.Generate the initial population matrix at random using Equation (2). $x_{i,d}\leftarrow lb_{d}+r\cdot \left(ub_{d}-lb_{d}\right)$4.Evaluate the objective function.5.
For t=1 to *T*6.
For i=1 to N7.
Phase 1: Attack on termite mounds (exploration phase)8.

Determine the termite mounds set for the *i*th GAO member using Equation (4). $TM_{i}\leftarrow \left\{X_{k_{i}}:F_{k_{i}}<F_{i}\;\mathrm{and}\;k_{i}\neq i\right\}$9.

Select the termite mounds for the *i*th GAO member at random.10.

Calculate new position of *i*th GAO member using Equation (5). $x_{i,d}^{P1}\leftarrow x_{i,d}+r\cdot \left(STM_{i,d}-I\cdot x_{i,d}\right)$11.

Update *i*th GAO member using Equation (6). $X_{i}\leftarrow \{\begin{array}{c} X_{i}^{P1},\;\;F_{i}^{P1}<F_{i}\\ X_{i},\;\;else \end{array}$12.
Phase 2: Digging in termite mounds (exploitation phase)13.

Calculate new position of *i*th GAO member using Equation (7). $x_{i,d}^{P2}\leftarrow x_{i,d}+\left(1-2r\right)\cdot \frac{\left(ub_{d}-lb_{d}\right)}{t}$14.

Update *i*th GAO member using Equation (8). $X_{i}\leftarrow \{\begin{array}{c} X_{i}^{P2},\;\;F_{i}^{P2}<F_{i}\\ X_{i},\;\;else \end{array}$15.
end16.

Save the best candidate solution so far.17.
end 18.Output the best quasi-optimal solution obtained with the GAO.End GAO.

### 3.5. Computational Complexity of GAO

In this subsection, the computational complexity of the proposed GAO approach is evaluated. The preparation and initialization process of GAO has a computational complexity equal to *O(Nm)*, where *N* is the number of giant armadillos and *m* is the number of decision variables of the problem. In the GAO design, in each iteration, the position of each giant armadillo is updated in two phases of exploration and exploitation. Therefore, the GAO update process has a computational complexity equal to *O(2NmT)*, where *T* is the maximum number of iterations of the algorithm. According to this, the total computational complexity of the proposed GAO approach is equal to *O(Nm(1 + 2T))*.

### 3.6. Comparing GAO vs. PSO

In this subsection, the proposed GAO approach is compared with PSO. PSO is a well-known bio-inspired metaheuristic algorithm that has been used in many optimization applications by researchers. 

In terms of the main design idea, PSO is inspired by the collective movement of groups of birds or fish that are searching for food. On the other hand, GAO was inspired by the giant armadillo’s strategy of attacking termite mounds and digging to feed on them. So, the difference in the main design idea is evident. 

In PSO, the position of each member of the population is updated according to the position of the best member of the population and the previous best position of the corresponding member. On the other hand, the position of each member of the population in the problem-solving space is updated based on the position of a better member (from the point of view of comparing the value of the objective function) and also based on local search management near each member’s position. 

A very important point in GAOs performance is that it has avoided a heavy dependence of the population update process on the best members. These conditions lead to the improvement of GAOs performance in global search management, preventing premature convergence, and preventing the algorithm from getting stuck in local optima. Meanwhile, in the design of PSO, the update process relies heavily on the position of the best member, which leads to inappropriate rapid convergence and stops the entire population from adopting a similar solution. 

Another important point in the design of metaheuristic algorithms is the control parameters. Determining the values of control parameters is a challenging process, and for this reason, the design of parameter-less approaches is considered a major advantage. The mathematical model of PSO has three control parameters, the value of which has a significant impact on the performance of this algorithm. This is despite the fact that no control parameters are included in the design of GAO, and from this point of view, GAO is a parameter-less approach.

## 4. Simulation Studies and Results

In this section, GAOs performance in solving optimization problems is evaluated. For this purpose, the efficiency of GAO is tested in handling the CEC 2017 test suite for problem dimensions equal to 10, 30, 50, and 100.

### 4.1. Performance Comparison

In order to measure the effectiveness of GAO in solving optimization problems, the obtained results are compared with the performance of twelve famous metaheuristic algorithms: GA [35], PSO [18], GSA [42], TLBO [56], MVO [44], GWO [22], WOA [25], MPA [31], TSA [32], RSA [27], AVOA [86], and WSO [33]. From the numerous optimization algorithms designed so far, these twelve methods have been selected for comparison with GAO. The reason for choosing these twelve competitor algorithms is that GA and PSO are the best-known and most widely used optimization algorithms. GSA, TLBO, MVO, and GWO, introduced between 2009 and 2016, have been popular methods for researchers and have been widely cited. WOA, MPA, and TSA algorithms are among the most widely used techniques introduced from 2016 to 2020. RSA, AVOA, and WSO are recently developed optimizers that have quickly gained the attention of scientists and have been used in a variety of real-world applications. The control parameter values of metaheuristic algorithms are specified in Appendix A and Table A1. The results of simulation studies are presented using six statistical indicators: mean, best, worst, standard deviation (std), median, and rank. The values obtained for the mean index are used as a ranking criterion for metaheuristic algorithms in handling each of the benchmark functions.

### 4.2. Evaluation of the CEC 2017 Test Suite

In this subsection, the performance of GAO and competitor algorithms is benchmarked in handling the CEC 2017 test suite for problem dimensions equal to 10, 30, 50, and 100. The CEC 2017 test suite has 30 standard benchmark functions consisting of (i) three unimodal functions of C17-F1 to C17-F3, (ii) seven multimodal functions of C17-F4 to C17-F10, (iii) ten hybrid functions of C17-F11 to C17-F20, and (iv) ten composition functions of C17-F21 to C17-F30. The C17-F2 functional is excluded from simulation studies due to its unstable behavior. Full information and more details about the CEC 2017 test suite are available at [87].

**Figure 3:**
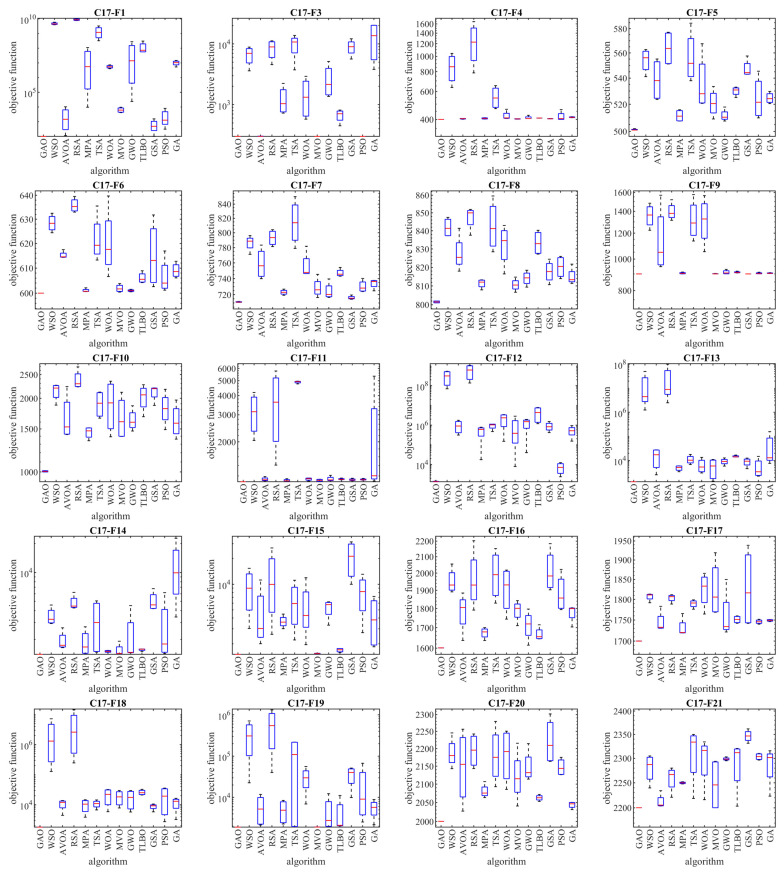

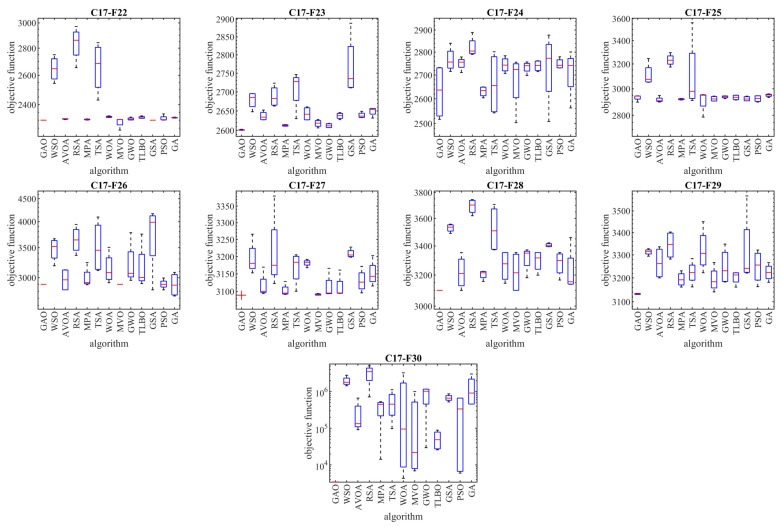
Boxplot diagrams of GAO and competitor algorithms’ performances on the CEC 2017 test suite (dimension = 10).

**Figure 4:**
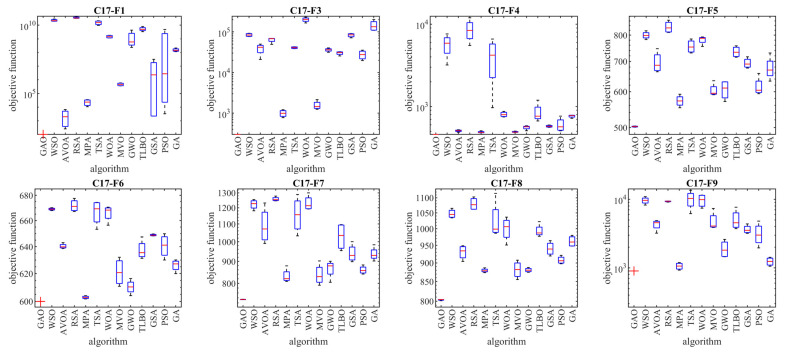

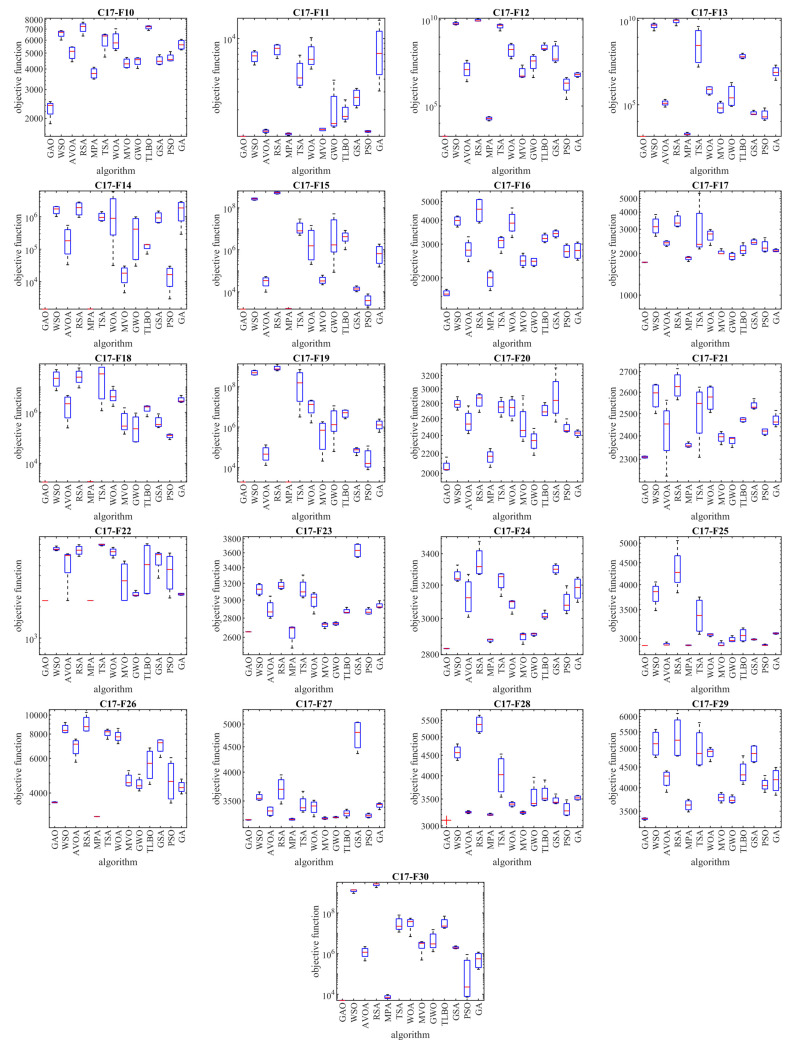
Boxplot diagrams of GAO and competitor algorithms’ performances on the CEC 2017 test suite (dimension = 30).

**Figure 5:**
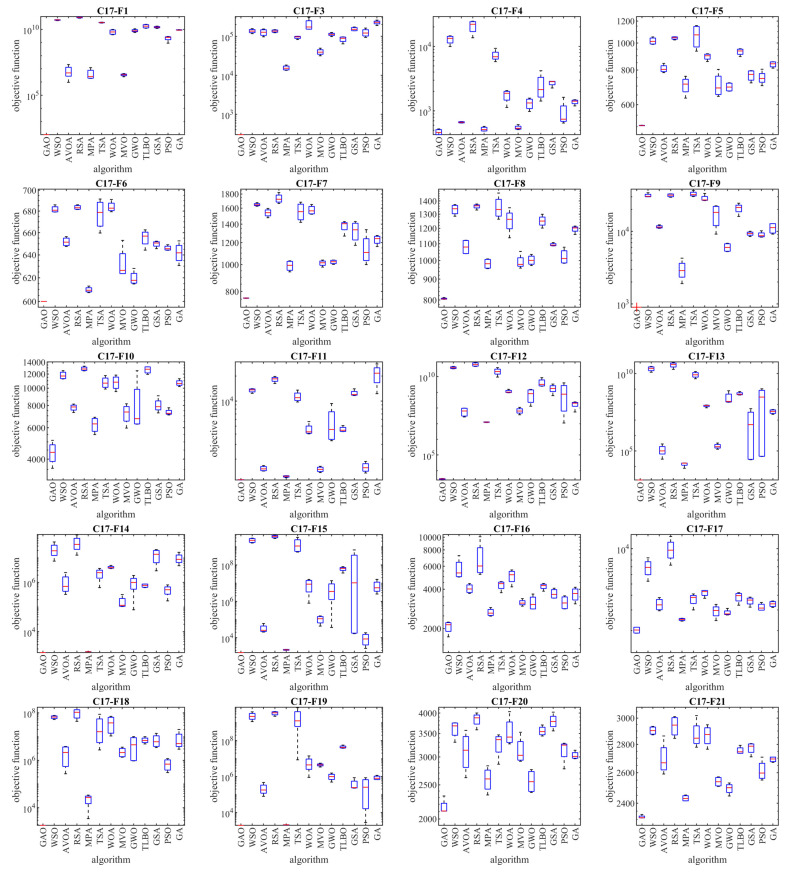

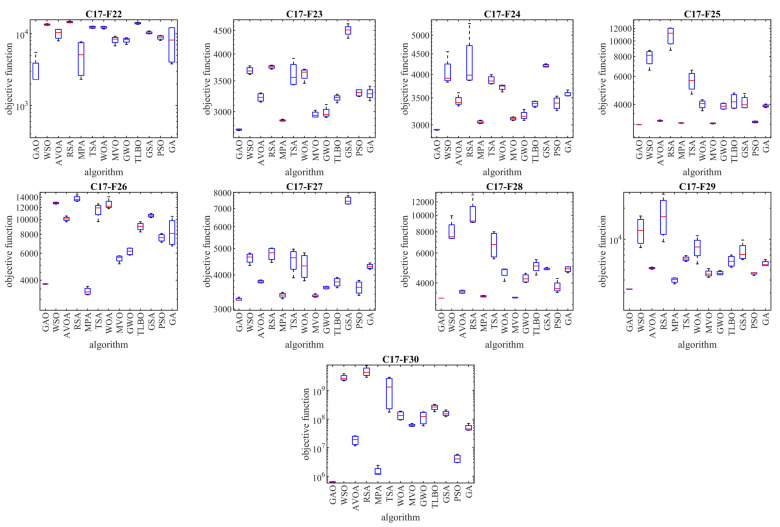
Boxplot diagrams of GAO and competitor algorithms’ performances on the CEC 2017 test suite (dimension = 50).

**Figure 6:**
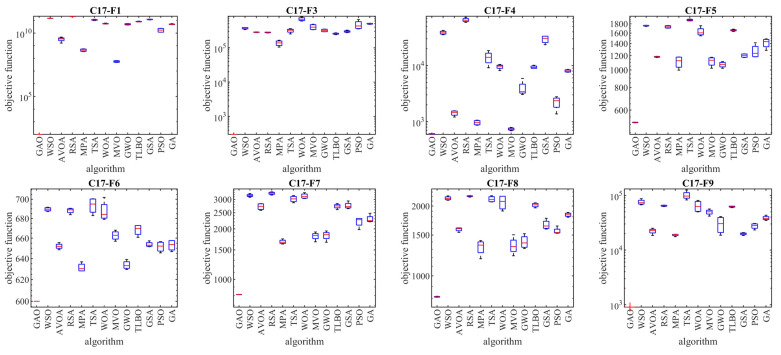

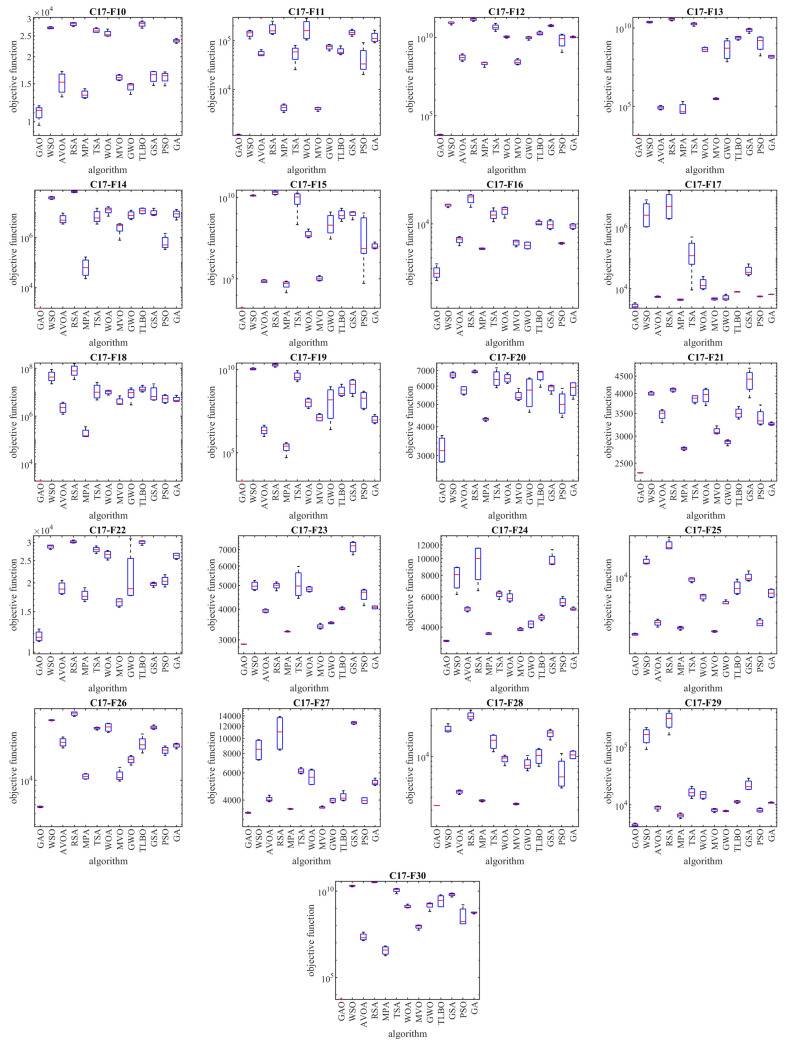
Boxplot diagrams of GAO and competitor algorithms’ performances on the CEC 2017 test suite (dimension = 100).

The implementation results of GAO and competitor algorithms on the CEC 2017 test suite are reported in Table 1, Table 2, Table 3 and Table 4. Boxplot diagrams obtained from the performance of metaheuristic algorithms are drawn in Figure 3, Figure 4, Figure 5 and Figure 6. 

Based on the analysis of the simulation results, the proposed GAO approach in handling the CEC 2017 test suite, for problem dimensions equal to 10 (*m* = 10), is the first best optimizer for functions C17-F1, C17-F3 to C17-F21, C17-F23, C17-F24, and C17-F27 to C17-F30 (i.e., 26 functions from 29 functions). Therefore, for problem dimensions equal to 10 (*m* = 10), GAO has been the first best optimizer in 26 out of 29 functions (i.e., 89.65% of test functions) and has provided superior performance compared to competing algorithms.

For problem dimensions equal to 30 (*m* = 30), the proposed GAO approach is the first best optimizer for functions C17-F1, C17-F3 to C17-F22, C17-F24, C17-F25, and C17-F27 to C17-F30. Therefore, for problem dimensions equal to 30 (*m* = 30), GAO has been the best optimizer in 27 out of 29 functions (i.e., 93.10% of test functions) and has provided superior performance compared to competing algorithms.

For problem dimensions equal to 50 (*m* = 50), the proposed GAO approach is the first best optimizer for functions C17-F1, C17-F3 to C17-F25, and C17-F27 to C17-F30. Therefore, for problem dimensions equal to 50 (*m* = 50), GAO has been the best optimizer in 28 out of 29 functions (i.e., 96.55% of test functions) and has provided superior performance compared to competing algorithms.

For problem dimensions equal to 100 (*m* = 100), the proposed GAO approach is the first best optimizer for functions C17-F1, C17-F3, and C17-F30. Therefore, for problem dimensions equal to 100 (*m* = 100), GAO has been the first best optimizer in 29 out of 29 functions (i.e., 100% of test functions) and has provided superior performance compared to competing algorithms.

The optimization results show that the proposed GAO approach has achieved good results for the benchmark functions, with high abilities in exploration, exploitation, and balance during the search process. What is clear from the simulation results is that GAO has provided superior performance by providing better results for most benchmark functions compared to competitor algorithms in dealing with the CEC 2017 test suite for problem dimensions equal to 10, 30, 50, and 100.

### 4.3. Statistical Analysis

In this subsection, using statistical analysis of the obtained results, it has been checked whether the superiority of the proposed GAO approach is significant from a statistical point of view or not. For this purpose, the Wilcoxon rank sum test [88] is employed, which is a non-parametric test and is used to determine the significant difference between the means of two data samples. In the Wilcoxon rank sum test, the presence or absence of a statistically significant difference is determined using an index called the *p*-value. The implementation results of the Wilcoxon rank sum test statistical analysis on the performance of GAO against each of the competitor algorithms are reported in Table 5. Based on the obtained results, in cases where the *p*-value is less than 0.05, GAO has a statistically significant superiority compared to the corresponding competitor algorithm. Statistical analysis shows that GAO has a significant statistical superiority in handling the CEC 2017 test suite for all four dimensions of the problem, equal to 10, 30, 50, and 100, in competition with all twelve compared algorithms.

## 5. GAO for Real-World Applications

In this section, the effectiveness of the proposed GAO approach in solving optimization problems in real-world applications is evaluated. For this purpose, twenty-two constrained optimization problems from the CEC 2011 test suite and four engineering design problems are selected.

### 5.1. Evaluation of the CEC 2011 Test Suite

In this subsection, the performance of GAO and competitor algorithms in handling the CEC 2011 test suite has been tested. The CEC 2011 test suite consists of twenty-two constrained optimization problems from real-world applications. A full description and more details about the CEC 2011 test suite are available at [89]. 

**Figure 7:**
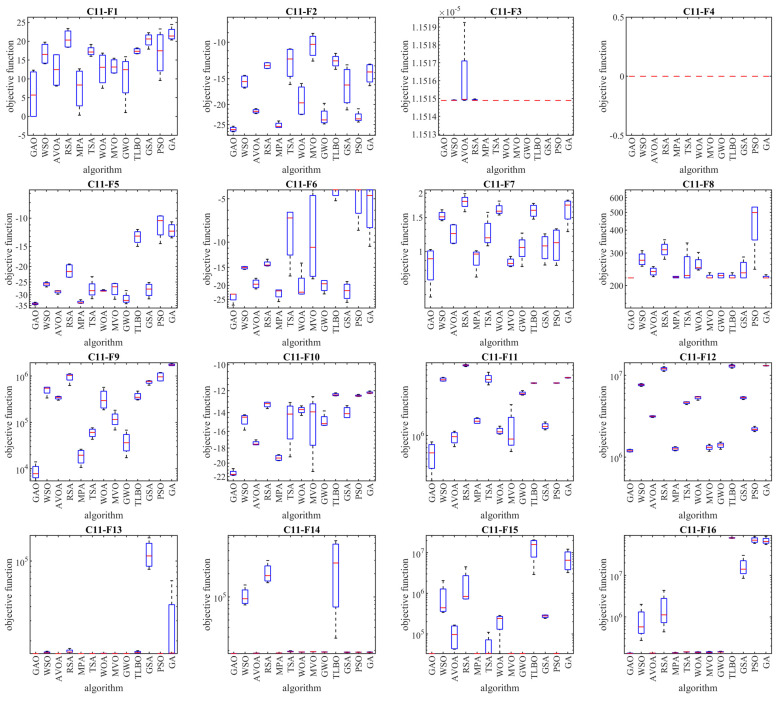

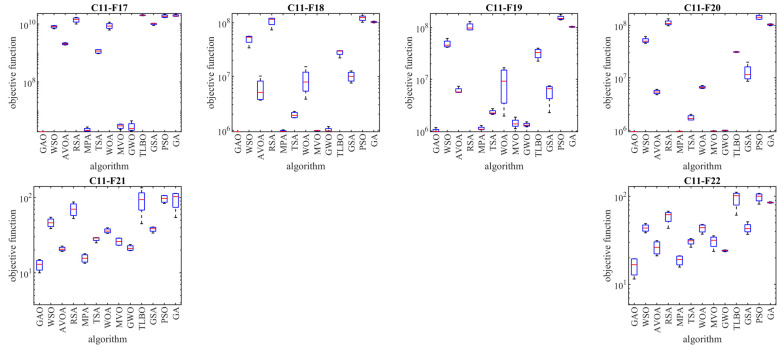
Boxplot diagrams of GAO and competitor algorithms’ performances on the CEC 2011 test suite.

The results of employing GAO and competitor algorithms to deal with the CEC 2011 test suite are reported in Table 6. The boxplot diagrams obtained from the performance of metaheuristic algorithms in this experiment are plotted in Figure 7. The optimization results show that the proposed GAO approach, with its high ability to explore, exploit, and balance them during the search process, has been able to provide suitable solutions for optimization problems. What is concluded from the comparison of the simulation results is that GAO has provided superior performance in handling the CEC 2011 test suite against competitor algorithms by providing better results for most of the benchmark functions and obtaining the rank of the first-best optimizer overall. Also, the results obtained from the Wilcoxon rank sum test indicate the statistically significant superiority of GAO compared to all twelve competitor algorithms in order to solve the CEC 2011 test suite.

### 5.2. Pressure Vessel Design Problem

**Figure 8:**
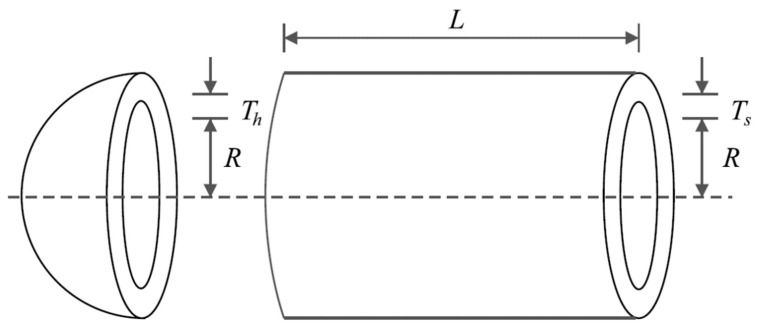
Schematic of pressure vessel design.

Pressure vessel design is a real-world engineering challenge with the aim of minimizing construction costs. The schematic of this design is shown in Figure 8, and its mathematical model is as follows [90]:

*Consider:* $X=\left[x_{1},\;x_{2},\;x_{3},\;x_{4}\right]=\left[T_{s},\;T_{h},\;R,\;L\right]$.

*Minimize:* $f\left(x\right)=0.6224x_{1}x_{3}x_{4}+1.778x_{2}x_{3}^{2}+3.1661x_{1}^{2}x_{4}+19.84x_{1}^{2}x_{3}.$

*Subject to:*

$$g_{1}\left(x\right)=-x_{1}+0.0193x_{3}\;\leq \;0,\;\;g_{2}\left(x\right)=-x_{2}+0.00954x_{3}\;\leq \;0,$$

$$g_{3}\left(x\right)=-\pi x_{3}^{2}x_{4}-\frac{4}{3}\pi x_{3}^{3}+1296,000\;\leq \;0,\;\;g_{4}\left(x\right)=x_{4}-240\;\leq \;0.$$

With

$$0\leq x_{1},x_{2}\leq 100\;\mathrm{and}\;10\leq x_{3},x_{4}\leq 200.$$

**Figure 9:**
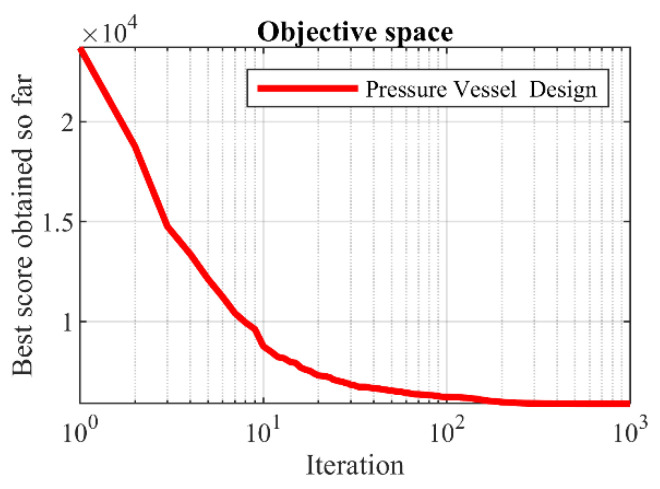
GAOs performance convergence curve on pressure vessel design.

The implementation results of the GAO and competitor algorithms on the Pressure vessel design problem are reported in Table 7 and Table 8. The convergence curve of GAO while achieving the optimal solution for pressure vessel design is drawn in Figure 9. Based on the optimization results, GAO has determined the optimal design for the pressure vessel with the values of the design variables equal to (0.7780271, 0.3845792, 40.312284, and 200) and the value of the objective function equal to (5882.8955). The simulation results show that GAO has provided superior performance in dealing with the pressure vessel design problem by providing better results compared to competitor algorithms.

### 5.3. Speed Reducer Design Problem

**Figure 10:**
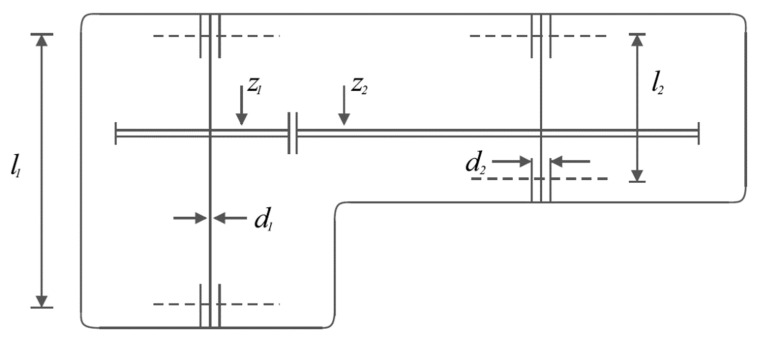
Schematic of speed reducer design.

Speed reducer design is a real-world engineering challenge with the aim of minimizing the weight of the speed reducer. The schematic of this design is shown in Figure 10, and its mathematical model is as follows [91,92]:

*Consider:* $X=\left[x_{1,}\;x_{2},\;x_{3},\;x_{4},\;x_{5}\;,x_{6}\;,x_{7}\right]=\left[b,\;m,\;p,\;l_{1},\;l_{2},\;d_{1},\;d_{2}\right]$.

*Minimize:* $f\left(x\right)=0.7854x_{1}x_{2}^{2}\left(3.3333x_{3}^{2}+14.9334x_{3}-43.0934\right)-1.508x_{1}\left(x_{6}^{2}+x_{7}^{2}\right)+7.4777\left(x_{6}^{3}+x_{7}^{3}\right)+0.7854\left(x_{4}x_{6}^{2}+x_{5}x_{7}^{2}\right)$.

*Subject to:* 

$$g_{1}\left(x\right)=\frac{27}{x_{1}x_{2}^{2}x_{3}}-1\;\leq \;0,\;\;\;g_{2}\left(x\right)=\frac{397.5}{x_{1}x_{2}^{2}x_{3}}-1\;\leq \;0,$$

$$g_{3}\left(x\right)=\frac{1.93x_{4}^{3}}{x_{2}x_{3}x_{6}^{4}}-1\;\leq \;0,\;\;\;g_{4}\left(x\right)=\frac{1.93x_{5}^{3}}{x_{2}x_{3}x_{7}^{4}}-1\;\leq \;0,$$

$$g_{5}\left(x\right)=\frac{1}{110x_{6}^{3}}\sqrt{{\left(\frac{745x_{4}}{x_{2}x_{3}}\right)}^{2}+16.9\times 10^{6}}-1\;\leq \;0,$$

$$g_{6}\left(x\right)=\frac{1}{85x_{7}^{3}}\sqrt{{\left(\frac{745x_{5}}{x_{2}x_{3}}\right)}^{2}+157.5\times 10^{6}}-1\;\leq \;0,$$

$$g_{7}\left(x\right)=\frac{x_{2}x_{3}}{40}-1\;\leq \;0,\;\;\;g_{8}\left(x\right)=\frac{5x_{2}}{x_{1}}-1\;\leq \;0,$$

$$g_{9}\left(x\right)=\frac{x_{1}}{12x_{2}}-1\;\leq \;0,\;\;\;g_{10}\left(x\right)=\frac{1.5x_{6}+1.9}{x_{4}}-1\;\leq \;0,$$

$$g_{11}\left(x\right)=\frac{1.1x_{7}+1.9}{x_{5}}-1\;\leq \;0.$$

With

$$2.6\leq x_{1}\leq 3.6,\;0.7\leq x_{2}\leq 0.8,\;17\leq x_{3}\leq 28,\;7.3\leq x_{4}\leq 8.3,\;7.8\leq x_{5}\;\leq 8.3,\;2.9\leq x_{6}\leq 3.9,\;\mathrm{and}\;5\leq x_{7}\leq 5.5.$$

**Figure 11:**
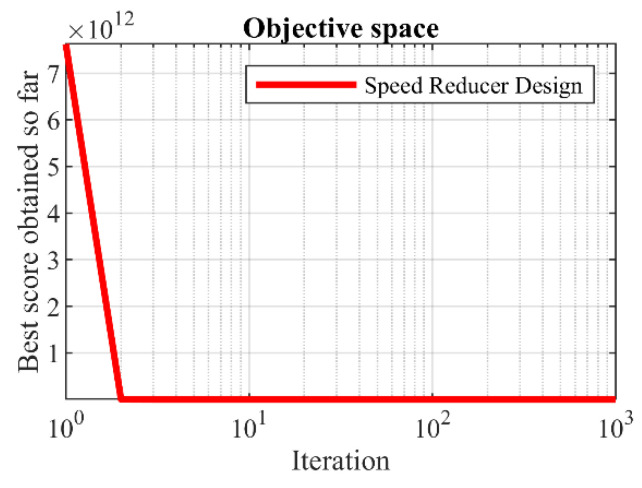
GAOs performance convergence curve on speed reducer design.

The results of employing GAO and competitor algorithms to solve the speed reducer design problem are reported in Table 9 and Table 10. The convergence curve of GAO towards the optimal solution for speed reducer design is drawn in Figure 11. Based on the optimization results, GAO has provided the optimal design for the speed reducer with the values of the design variables equal to (3.5, 0.7, 17, 7.3, 7.8, 3.3502147, and 5.2866832) and the value of the objective function equal to (2996.3482). Analysis of the simulation results indicates that GAO has provided superior performance by achieving better results in order to solve the problem of speed reducer design compared to competitor algorithms. 

### 5.4. Welded Beam Design

**Figure 12:**
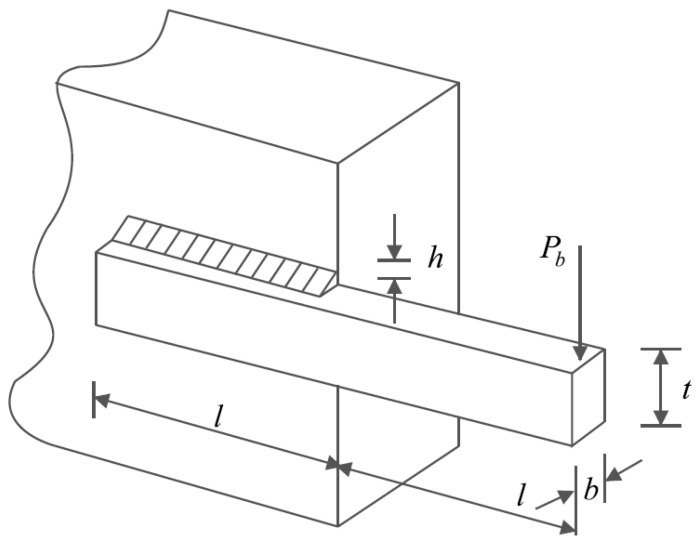
Schematic of welded beam design.

Welded beam design is a real-world engineering challenge with the aim of minimizing the fabrication cost of the welded beam. The schematic of this design is shown in Figure 12, and its mathematical model is as follows [25]:

*Consider:* $X=\left[x_{1},\;x_{2},\;x_{3},\;x_{4}\right]=\left[h,\;l,\;t,\;b\right]$.

*Minimize:* $f\left(x\right)=1.10471x_{1}^{2}x_{2}+0.04811x_{3}x_{4}\;\left(14.0+x_{2}\right)$.

*Subject to:*

$$g_{1}\left(x\right)=\tau \left(x\right)-13,600\;\leq \;0,\;\;g_{2}\left(x\right)=\sigma \left(x\right)-30,000\;\leq \;0,$$

$$g_{3}\left(x\right)=x_{1}-x_{4}\;\leq \;0,\;\;g_{4}\left(x\right)=0.10471x_{1}^{2}+0.04811x_{3}x_{4}\;\left(14+x_{2}\right)-5.0\;\leq \;0,$$

$$g_{5}\left(x\right)=0.125-x_{1}\;\leq \;0,\;\;g_{6}\left(x\right)=\delta \;\left(x\right)-0.25\;\leq \;0,$$

$$g_{7}\left(x\right)=6000-p_{c}\;\left(x\right)\;\leq \;0.$$

where

$$\tau \left(x\right)=\sqrt{{\left(\tau^{\prime }\right)}^{2}+\left(2\tau \tau^{\prime }\right)\frac{x_{2}}{2R}+{\left(\tau \prime \prime \right)}^{2}\;},\;\;\tau^{\prime }=\frac{6000}{\sqrt{2}x_{1}x_{2}},\;\;\tau \prime \prime =\frac{MR}{J},$$

$$M=6000\left(14+\frac{x_{2}}{2}\right),\;\;R=\sqrt{\frac{x_{2}^{2}}{4}+{\left(\frac{x_{1}+x_{3}}{2}\right)}^{2}},$$

$$J=2\left\{x_{1}x_{2}\sqrt{2}\left[\frac{x_{2}^{2}}{12}+{\left(\frac{x_{1}+x_{3}}{2}\right)}^{2}\right]\right\},\;\;\;\sigma \left(x\right)=\frac{504,000}{x_{4}x_{3}^{2}}\;$$

$$\delta \;\left(x\right)=\frac{65,856,000}{\left(30\cdot 10^{6}\right)x_{4}x_{3}^{3}},\;\;p_{c}\;\left(x\right)=\frac{4.013\left(30\cdot 10^{6}\right)\sqrt{\frac{x_{3}^{2}x_{4}^{6}}{36}}}{196}\left(1-\frac{x_{3}}{28}\sqrt{\frac{30\cdot 10^{6}}{4\left(12\cdot 10^{6}\right)}}\right).$$

With

$$0.1\leq x_{1},\;x_{4}\leq 2\;\;\;\mathrm{and}\;0.1\leq x_{2},\;x_{3}\leq 10.$$

**Figure 13:**
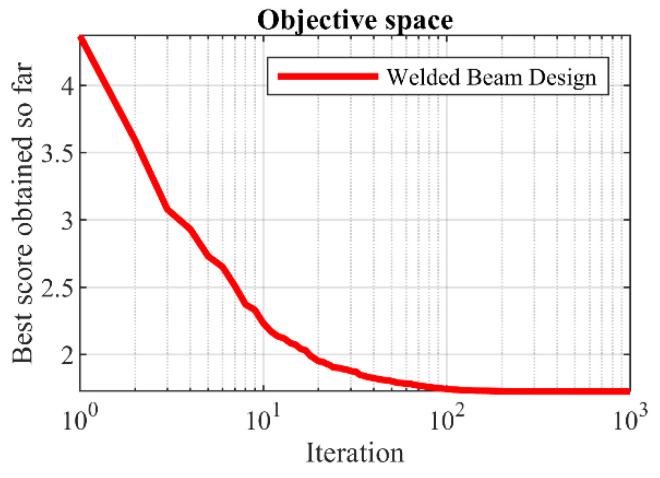
GAOs performance convergence curve on welded beam design.

The results of dealing with the problem of welded beam design using GAO and competitor algorithms are reported in Table 11 and Table 12. The convergence curve of GAO while achieving the optimal solution for welded beam design is drawn in Figure 13. Based on the optimization results, GAO has determined the optimal design for the welded beam with the values of the design variables equal to (0.2057296, 3.4704887, 9.0366239, and 0.2057296) and the value of the objective function equal to (1.7246798). What is evident from the simulation results is that GAO has provided superior performance by converging to better results in order to address the welded beam design problem compared to competitor algorithms.

### 5.5. Tension/Compression Spring Design 

**Figure 14:**
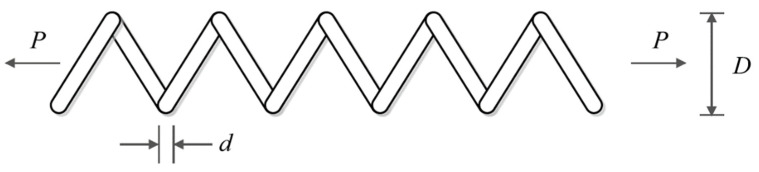
Schematic of tension/compression spring design.

Tension/compression spring design is a real-world engineering challenge with the aim of minimizing the weight of the tension/compression spring. The schematic of this design is shown in Figure 14, and its mathematical model is as follows [25]:

*Consider:* $X=\left[x_{1},\;x_{2},\;x_{3}\;\right]=\left[d,\;D,\;P\right].$

*Minimize:* $f\left(x\right)=\left(x_{3}+2\right)x_{2}x_{1}^{2}.$

*Subject to:*

$$g_{1}\left(x\right)=1-\frac{x_{2}^{3}x_{3}}{71,785x_{1}^{4}}\;\leq \;0,\;\;g_{2}\left(x\right)=\frac{4x_{2}^{2}-x_{1}x_{2}}{12,566\left(x_{2}x_{1}^{3}\right)}+\frac{1}{5108x_{1}^{2}}-1\;\leq \;0,$$

$$g_{3}\left(x\right)=1-\frac{140.45x_{1}}{x_{2}^{2}x_{3}}\;\leq \;0,\;\;g_{4}\left(x\right)=\frac{x_{1}+x_{2}}{1.5}-1\;\leq \;0.$$

With

$$0.05\leq x_{1}\leq 2,\;0.25\leq x_{2}\leq 1.3\;\;\;\;\mathrm{and}\;\;\;\;2\leq \;x_{3}\leq 15$$

**Figure 15:**
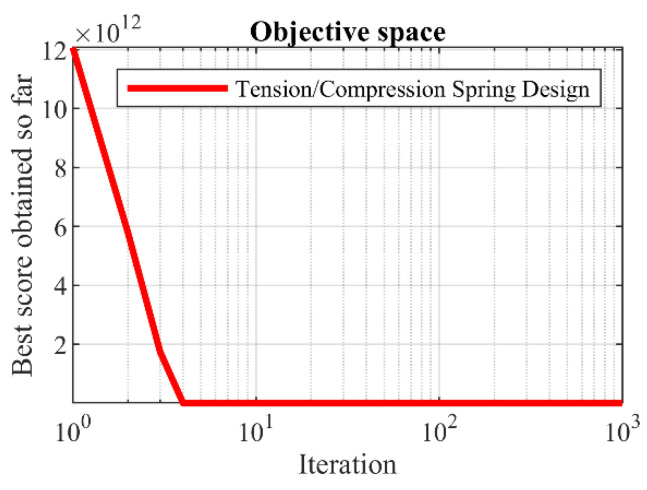
GAOs performance convergence curve on tension/compression spring.

The implementation results of GAO and competitor algorithms on the tension/compression spring design problem are reported in Table 13 and Table 14. The convergence curve of GAO towards the optimal solution for tension/compression spring design is drawn in Figure 15. Based on the optimization results, GAO has determined the optimal design for the tension/compression spring with the values of the design variables equal to (0.0516891, 0.3567177, and 11.288966) and the value of the objective function equal to (0.0126019). The simulation results show that GAO has provided superior performance by providing better results for solving the tension/compression spring design problem compared to competitor algorithms. 

## 6. Conclusions and Future Works

In this paper, a new bio-inspired metaheuristic algorithm called Giant Armadillo Optimization (GAO) was introduced, which imitates the behavior of giant armadilloes in nature. The fundamental inspiration for GAOs design is derived from the attack strategy of giant armadillos in moving towards prey positions and digging termite mounds. The GAO theory was stated, and its implementation steps were mathematically modeled in two phases: (i) exploration based on the simulation of the movement of giant armadillos towards termite mounds, and (ii) exploitation based on the simulation of the giant armadillo’s digging skills in order to prey on and rip open termite mounds. The efficiency of GAO was evaluated in handling the CEC 2017 test suite for problem dimensions equal to 10, 30, 50, and 100. The optimization results showed that GAO has a high ability for exploration, exploitation, and balancing them during the search process. The results obtained from GAO were compared with the performance of twelve well-known metaheuristic algorithms. The simulation results showed that GAO has provided superior performance by achieving better results for most of the benchmark functions in competition with competitor algorithms. Using the statistical analysis of the Wilcoxon rank sum test, it was confirmed that GAO has a significant statistical superiority over competitor algorithms. Implementation of GAO on the CEC 2011 test suite and four engineering design problems showed that the proposed approach has an effective ability to handle optimization tasks in real-world applications.

Introducing the proposed GAO approach raises several research tasks for further work.

- **Binary GAO.** The real version of GAO is introduced and fully designed in this paper. However, many optimization problems in science, such as feature selection, should be optimized using binary versions of metaheuristic algorithms. According to this, designing the binary version of the proposed GAO approach (BGAO) is one of the special potentials of this study.
- **Multi-objective GAO.** From the point of view of the number of objective functions, optimization problems are divided into single-objective and multi-objective categories. In many optimization problems, several objective functions must be considered simultaneously in order to achieve a suitable solution. Therefore, developing the multi-objective version of the proposed GAO approach (MOGAO) in order to handle multi-objective optimization problems is another research potential of this paper.
- **Hybrid GAO.** Combining two or more metaheuristic algorithms in order to benefit from the advantages of each algorithm and create an effective hybrid approach has always been of interest to researchers. Considering this, developing hybrid versions of the proposed GAO approach is another research proposal for future work.
- **Tackle new domains**. GAO employment to address real-world applications and optimization problems in various sciences such as renewable energy, chemical engineering, robotics, and image processing are among other research proposals for further work.

## Figures and Tables

**Table 1:** Optimization results of the CEC 2017 test suite (dimension = 10).

		GAO	WSO	AVOA	RSA	MPA	TSA	WOA	MVO	GWO	TLBO	GSA	PSO	GA
C17-F1	mean	100	4.67E+09	4,184,979	8.73E+09	34,330,739	1.49E+09	9,692,832	4,188,121	79,553,526	1.3E+08	4,182,333	4,184,382	14,308,636
	best	100	3.93E+09	3746.743	7.56E+09	10,903.05	3.19E+08	4,981,868	8834.837	25,081.34	56,192,560	1415.894	1625.786	6,732,111
	worst	100	5.85E+09	15,192,929	1.04E+10	1.25E+08	3.24E+09	19,205,398	15,201,956	2.89E+08	3.03E+08	15,192,873	15,193,484	26,519,809
	std	0	9.08E+08	8,022,380	1.4E+09	65,875,245	1.41E+09	7,002,724	8,026,144	1.53E+08	1.27E+08	8,023,882	8,022,667	9,534,136
	median	100	4.44E+09	771,620	8.48E+09	6,292,615	1.2E+09	7,292,030	770,846.8	14,580,526	80,111,109	767,520.9	771,208.3	11,991,311
	rank	1	12	4	13	8	11	6	5	9	10	2	3	7
C17-F3	mean	300	6653.076	432.8431	8416.704	1377.331	9744.62	1652.665	431.2721	2796.49	795.3626	8937.772	431.2255	12,795.02
	best	300	3819.149	358.2124	4752.444	777.91	3952.712	640.1711	358.2232	1407.456	504.4881	5625.365	358.2123	4024.146
	worst	300	8784.409	568.2645	11,138.08	2473.984	13,631.64	2947.044	564.9107	5337.754	1012.646	12,088.54	564.8045	20,125.74
	std	0	2369.321	104.7758	3175.356	851.6238	4471.714	1195.34	103.7782	1973.46	249.9022	2890.252	103.7329	9176.458
	median	300	7004.373	402.4478	8888.145	1128.715	10,697.06	1511.722	400.9773	2220.375	832.1585	9018.593	400.9426	13,515.1
	rank	1	9	4	10	6	12	7	3	8	5	11	2	13
C17-F4	mean	400	843.9185	404.8598	1213.807	406.5485	551.6274	422.2989	403.6485	410.833	408.6382	404.6903	418.1641	413.3818
	best	400	633.9161	401.4788	781.7224	402.3817	466.9688	406.857	401.7805	405.4965	408.3648	403.4626	400.3805	411.3341
	worst	400	1034.209	406.7134	1637.197	411.0784	649.5213	463.1789	405.3186	425.5967	409.1148	406.3282	461.3011	416.8981
	std	0	195.1214	2.525052	397.3324	4.668634	97.40747	29.75653	1.658473	10.72221	0.357177	1.561991	31.59144	2.777032
	median	400	853.7743	405.6236	1218.153	406.3669	545.0096	409.5799	403.7475	406.1195	408.5367	404.4852	405.4875	412.6476
	rank	1	12	4	13	5	11	10	2	7	6	3	9	8
C17-F5	mean	501.2464	554.7301	539.6017	564.4364	512.703	557.1398	536.9462	522.0381	512.825	530.977	548.0728	525.6662	525.7627
	best	500.9951	541.5438	525.3512	551.2702	508.2545	539.3227	521.9911	510.8276	508.4225	525.7325	543.4038	510.6874	522.1256
	worst	501.9917	562.7056	556.257	577.8185	517.7245	584.2697	567.3886	533.8411	519.724	534.6331	557.7146	546.6921	531.3457
	std	0.540776	10.53806	17.74322	16.11171	5.423907	21.90443	23.23182	10.3802	5.314383	4.281877	7.152264	18.18017	4.645014
	median	500.9993	557.3354	538.3993	564.3285	512.4164	552.4834	529.2026	521.7418	511.5768	531.7711	545.5865	522.6426	524.7898
	rank	1	11	9	13	2	12	8	4	3	7	10	5	6
C17-F6	mean	600	628.1172	615.1576	635.431	601.1785	621.6683	620.2268	602.0072	601.1205	606.0926	615.0587	606.5844	609.0379
	best	600	624.1437	614.3677	632.6039	600.7017	613.1533	606.6099	600.5106	600.6181	604.2107	602.6271	601.2733	606.0714
	worst	600	632.1329	617.3273	639.0601	602.3672	635.1389	639.2844	604.0274	601.5891	609.0813	631.4329	616.984	612.8624
	std	0	3.743769	1.575387	3.181093	0.864759	10.29375	14.95242	1.717729	0.458809	2.412521	14.53307	7.76462	3.27032
	median	600	628.0961	614.4676	635.0299	600.8225	619.1905	617.5064	601.7454	601.1374	605.5392	613.0873	604.0401	608.609
	rank	1	12	9	13	3	11	10	4	2	5	8	6	7
C17-F7	mean	711.1267	786.7696	759.9427	793.5837	724.4666	814.493	756.9305	729.8769	725.6592	748.2351	717.9475	731.4933	735.0744
	best	710.6726	772.6067	741.6925	782.6387	720.3138	780.2592	746.903	717.8787	717.7416	744.8348	715.8297	725.1484	725.9664
	worst	711.7995	796.765	783.5109	804.6758	728.8251	850.564	782.3197	747.11	741.377	755.3906	721.2986	741.6302	739.1596
	std	0.557384	11.05161	21.09388	11.22471	3.89965	33.21785	18.51036	13.41155	11.73144	5.310127	2.641472	8.015211	6.79059
	median	711.0174	788.8534	757.2838	793.5102	724.3638	813.5744	749.2497	727.2596	721.7591	746.3575	717.331	729.5974	737.5857
	rank	1	11	10	12	3	13	9	5	4	8	2	6	7
C17-F8	mean	801.4928	842.6739	828.5432	848.1225	812.5351	843.4306	833.0884	811.8088	815.2945	834.2569	818.779	821.2983	816.1124
	best	800.995	838.1575	818.6788	838.3745	808.7549	829.3668	817.8831	808.206	810.2093	828.4819	812.1894	815.3777	812.8734
	worst	801.9912	847.7998	842.4782	852.205	814.6617	859.7064	843.6564	815.4968	819.8403	840.725	825.0554	827.1608	822.4095
	std	0.625636	5.545568	10.87967	7.138078	2.962099	14.70256	12.09117	3.247739	4.406927	7.021296	6.025675	6.451529	4.653363
	median	801.4926	842.3692	826.5079	850.9554	813.3619	842.3245	835.4072	811.7662	815.5642	833.9103	818.9357	821.3274	814.5833
	rank	1	11	8	13	3	12	9	2	4	10	6	7	5
C17-F9	mean	900	1353.7	1150.517	1392.659	905.134	1317.56	1312.862	901.3205	910.9771	910.8839	900.6254	904.305	905.059
	best	900	1221.615	946.6395	1309.138	900.3235	1133.13	1051.687	900.1139	900.5366	907.8785	900.0394	900.8912	903.0891
	worst	900	1475.045	1561.802	1512.884	913.1785	1566.988	1556.076	903.3636	930.3434	917.5471	901.6052	910.8806	907.9139
	std	0	119.594	310.193	94.4051	6.297156	204.4421	230.9694	1.659842	15.1837	4.893863	0.767032	4.871949	2.216612
	median	900	1359.07	1046.813	1374.307	903.517	1285.061	1321.842	900.9022	906.5142	909.0551	900.4284	902.7241	904.6165
	rank	1	12	9	13	6	11	10	3	8	7	2	4	5
C17-F10	mean	1006.179	2181.096	1729.586	2417.519	1505.229	1948.592	1942.061	1732.136	1684.558	2068.376	2159.534	1874.018	1676.188
	best	1000.284	1921.987	1462.046	2268.381	1382.756	1720.911	1445.208	1441.456	1509.563	1717.81	1916.982	1528.099	1415.382
	worst	1012.668	2300.117	2273.835	2734.951	1578.549	2151.824	2401.429	2171.491	1922.565	2324.822	2258.83	2231.734	2024.674
	std	7.244311	192.7589	408.7834	235.1122	100.3704	255.5173	495.8664	379.7598	188.8268	281.6863	176.5855	314.8046	282.3401
	median	1005.882	2251.141	1591.231	2333.372	1529.806	1960.816	1960.804	1657.799	1653.051	2115.435	2231.162	1868.12	1632.348
	rank	1	12	5	13	2	9	8	6	4	10	11	7	3
C17-F11	mean	1100	3101.99	1144.84	3578.148	1126.427	4844.162	1146.945	1126.823	1150.648	1146.906	1136.85	1140.57	2203.963
	best	1100	2031.676	1121.626	1410.344	1112.898	4715.435	1118.117	1107.382	1120.125	1135.086	1123.853	1129.361	1119.906
	worst	1100	4144.434	1188.913	5717.17	1157.436	4912.302	1165.353	1143.543	1217.154	1163.618	1160.564	1162.792	5289.488
	std	0	1033.315	33.04664	2107.652	22.88817	95.80871	23.58632	18.64388	49.2607	13.17059	17.71381	16.44602	2239.144
	median	1100	3115.925	1134.41	3592.539	1117.687	4874.455	1152.156	1128.182	1132.656	1144.46	1131.492	1135.064	1203.23
	rank	1	11	6	12	2	13	8	3	9	7	4	5	10
C17-F12	mean	1352.959	3.04E+08	1,014,691	6.07E+08	55,6081.8	962,283.6	2,093,142	953,191.8	1,285,489	4,414,980	945,683.3	74,718.57	588,303.7
	best	1318.646	68,431,780	396,241.9	1.35E+08	19,486.36	466,272.3	220,440.9	80,269.96	41,489.41	1,236,043	498,216.7	11,672.08	240,713.7
	worst	1438.176	5.31E+08	1,719,649	1.06E+09	870,237	1,186,611	3,466,069	2,783,658	2,012,132	7,801,921	1,487,109	118,000.4	991,573.3
	std	62.35801	2.55E+08	689,369.2	5.1E+08	407,890	368,442.3	1,616,452	1,348,855	945,244.5	3,753,489	457,630	49,610.7	344,419.3
	median	1327.506	3.09E+08	971,435.4	6.16E+08	667,302	1098,125	2,343,029	474,419.4	1,544,168	4,310,979	898,703.8	84,600.92	560,463.9
	rank	1	12	8	13	3	7	10	6	9	11	5	2	4
C17-F13	mean	1305.324	14,793,370	16,446.49	29,577,769	5350.291	11,633.32	7195.222	6463.604	9535.39	15,064.64	9339.47	6371.288	47,520.14
	best	1303.114	1,233,731	3162.283	2,456,038	3671.563	7329.324	3624.201	2012.063	6068.856	14,058.19	4950.777	2866.249	8151.738
	worst	1308.508	49,102,055	27,494.33	98,191,200	6537.327	17,832.22	13,645.16	11,451.69	13,197.34	17,150.1	13,023.45	15,181.42	15,5719
	std	2.473462	24,946,024	13,820.39	49,889,389	1487.51	4917.991	4963.144	5371.09	3183.327	1533.341	3671.92	6442.862	78,543.6
	median	1304.837	4,418,847	17,564.68	8,831,920	5596.137	10,685.86	5755.762	6195.332	9437.683	14,525.13	9691.829	3718.742	13,104.94
	rank	1	12	10	13	2	8	5	4	7	9	6	3	11
C17-F14	mean	1400.746	3537.165	2000.121	4863.875	1929.741	3175.526	1567.073	1612.61	2279.672	1628.918	5052.126	2838.871	11,422.2
	best	1400	2917.026	1698.168	4293.595	1434.361	1481.885	1482.685	1426.054	1466.642	1506.182	4199.684	1432.2	3408.166
	worst	1400.995	4533.216	2636.156	6138.936	2874.521	5007.289	1705.952	2090.877	4648.74	1770.088	6879.61	6156.929	22,446.14
	std	0.541408	769.4616	466.7728	942.5869	735.1115	2005.623	104.9446	348.6518	1719.029	120.5646	1372.454	2439.604	8753.404
	median	1400.995	3349.208	1833.079	4511.485	1705.042	3106.466	1539.828	1466.754	1501.653	1619.702	4564.605	1883.177	9917.238
	rank	1	10	6	11	5	9	2	3	7	4	12	8	13
C17-F15	mean	1500.331	9291.205	5066.766	12,452.89	3928.049	6534.761	5859.352	1831.798	5511.379	1975.935	21,070.59	8252.547	4422.54
	best	1500.001	3298.851	2198.906	2860.359	3190.009	2503.361	2217.57	1728.182	3488.756	1843.486	10,151.05	2955.741	2241.563
	worst	1500.5	15,478.21	11,357.34	26,628.87	4826.524	11,218.82	12,194.46	1938.978	6425.035	2050.737	31,372.95	13,354.66	7407.664
	std	0.256213	5737.638	4611.449	11,314.8	738.847	4057.645	4744.95	94.03401	1499.643	101.8412	10,985.2	4717.115	2809.182
	median	1500.413	9193.879	3355.407	10,161.17	3847.831	6208.432	4512.689	1830.016	6065.863	2004.758	21,379.17	8349.895	4020.466
	rank	1	11	6	12	4	9	8	2	7	3	13	10	5
C17-F16	mean	1600.76	1956.927	1790.531	1968.643	1682.603	1995.231	1911.866	1796.276	1720.719	1676.29	2017.507	1888.754	1784.315
	best	1600.356	1899.128	1650.114	1802.593	1640.957	1830.958	1751.897	1713.956	1618.636	1655.686	1903.889	1801.282	1715.952
	worst	1601.12	2058.375	1885.918	2204.09	1712.608	2153.805	2017.206	1853.043	1807.866	1726.683	2188.933	2028.071	1812.82
	std	0.343807	77.00688	108.6182	184.7761	33.54393	159.3833	139.0817	64.0361	84.9012	37.02768	140.3106	112.9385	49.86364
	median	1600.781	1935.103	1813.047	1933.945	1688.423	1998.08	1939.18	1809.054	1728.188	1661.396	1988.604	1862.831	1804.244
	rank	1	10	6	11	3	12	9	7	4	2	13	8	5
C17-F17	mean	1700.099	1809.53	1748.189	1806.239	1735.059	1792.301	1826.501	1827.26	1763.306	1754.568	1830.699	1749.382	1752.501
	best	1700.02	1793.174	1732.49	1796.351	1721.495	1777.798	1766.075	1770.48	1723.924	1744.182	1743.916	1742.235	1748.199
	worst	1700.332	1819.041	1784.458	1812.531	1773.486	1800.078	1872.055	1924.708	1856.768	1765.263	1944.2	1755.838	1758.536
	std	0.168864	12.33483	26.48996	7.632948	27.89407	10.77603	49.38603	79.30684	68.11292	11.30402	110.1293	6.920241	4.861874
	median	1700.022	1812.953	1737.905	1808.038	1722.628	1795.665	1833.938	1806.927	1736.266	1754.414	1817.34	1749.728	1751.634
	rank	1	10	3	9	2	8	11	12	7	6	13	4	5
C17-F18	mean	1805.36	2,456,562	11,541.03	4,895,611	10,847.99	11,714.98	21,376.33	19,348.63	18,454.43	26,699.35	9699.424	20,146.85	12,363.66
	best	1800.003	128,322.8	5943.256	244,067.4	4107.461	8195.069	6640.072	8574.872	5967.329	21,142.43	6592.158	4256.342	4733.467
	worst	1820.451	7,116,657	15,404.74	14,209,754	16,196.96	14,526.12	31,980.65	30,964.52	30,634.13	32,797.95	11,966.24	36,094.26	17,883.82
	std	10.95197	3,522,742	4423.846	7,042,499	5983.7	2856.445	13,538.96	11,110.68	13,611.52	5737.733	2540.637	18,444.41	6095.706
	median	1800.492	1,290,633	12,408.07	2,564,312	11,543.77	12,069.36	23,442.29	18,927.56	18,608.14	26,428.51	10,119.65	20,118.4	13,418.68
	rank	1	12	4	13	3	5	10	8	7	11	2	9	6
C17-F19	mean	1900.445	333,554.7	6471.06	605,336	5517.557	108,525	30,605.59	2353.324	5333.283	4743.012	35,431.25	22,136.66	6019.454
	best	1900.039	22,357.55	2187.576	39,716.99	2308.741	2080.283	6943.315	1957.954	2082.111	2211.714	10,526.97	2618.255	2890.56
	worst	1901.559	701,682.7	12,365.65	1,299,174	9251.813	216,523.7	55,721.2	2809.084	12,850.83	11,045.82	50,727.43	67,203.92	9652.432
	std	0.810364	323,164.3	5110.522	618,250.1	3851.325	133,408.8	21,755.74	467.5256	5493.958	4585.206	19,767.59	33,107.07	3050.353
	median	1900.09	305,089.2	5665.506	541,226.6	5254.838	107,747.9	29,878.93	2323.129	3200.098	2857.258	40,235.29	9362.235	5767.412
	rank	1	12	7	13	5	11	9	2	4	3	10	8	6
C17-F20	mean	2000.312	2195.65	2157.506	2202.571	2090.307	2189.132	2188.459	2130.878	2156.952	2072.851	2228.919	2156.148	2054.136
	best	2000.312	2151.12	2041.552	2151.909	2071.162	2101.769	2099.1	2050.91	2121.121	2061.048	2171.882	2135.012	2041.352
	worst	2000.312	2250.869	2261.632	2253.811	2120.147	2284.313	2255.949	2221.207	2225.944	2081.388	2312.523	2181.17	2059.917
	std	0	44.83411	108.1238	53.54076	22.84341	83.61951	82.41672	75.96454	51.25916	10.0526	74.5843	23.99981	9.348874
	median	2000.312	2190.305	2163.42	2202.282	2084.959	2185.224	2199.393	2125.698	2140.371	2074.485	2215.636	2154.205	2057.638
	rank	1	11	8	12	4	10	9	5	7	3	13	6	2
C17-F21	mean	2200	2285.515	2218.687	2264.516	2255.984	2314.411	2301.249	2252.5	2304.203	2292.508	2351.495	2308.934	2291.201
	best	2200	2245.804	2210.67	2227.49	2253.548	2224.984	2222.545	2206.529	2300.289	2210.095	2336.176	2301.709	2229.95
	worst	2200	2310.048	2240.432	2285.317	2258.467	2354.854	2338.939	2299.234	2308.777	2325.466	2366.304	2315.505	2320.685
	std	0	31.96962	15.80852	27.93412	2.265614	66.04131	57.78516	57.40188	3.79845	60.19259	13.68159	7.42458	44.98868
	median	2200	2293.103	2211.824	2272.629	2255.961	2338.903	2321.756	2252.119	2303.872	2317.235	2351.751	2309.262	2307.086
	rank	1	6	2	5	4	12	9	3	10	8	13	11	7
C17-F22	mean	2300.073	2642.8	2308.325	2830.323	2304.903	2656.585	2321.07	2288.364	2307.996	2317.434	2300.603	2312.013	2316.02
	best	2300	2540.302	2304.87	2650.497	2300.924	2428.398	2317.164	2239.419	2301.202	2311.909	2300.113	2300.661	2313.04
	worst	2300.29	2745.079	2309.73	2962.713	2309.169	2835.901	2327.16	2305.128	2320.393	2328.049	2301.117	2339.816	2319.724
	std	0.157893	98.44184	2.533489	143.0457	3.781106	197.8878	4.924207	35.51645	9.483233	8.127414	0.457965	20.24666	3.028103
	median	2300	2642.909	2309.351	2854.041	2304.759	2681.021	2319.979	2304.455	2305.195	2314.89	2300.591	2303.787	2315.658
	rank	2	11	6	13	4	12	10	1	5	9	3	7	8
C17-F23	mean	2600.919	2678.961	2638.02	2688.432	2614.073	2708.104	2643.747	2619.19	2613.571	2638.43	2767.027	2639.931	2650.15
	best	2600.003	2649.146	2627.825	2663.205	2611.722	2631.522	2628.648	2608.07	2608.201	2629.366	2711.138	2633.477	2633.265
	worst	2602.87	2696.413	2653.633	2723.717	2616.706	2746.175	2660.892	2629.469	2619.663	2646.197	2886.071	2649.994	2657.13
	std	1.436922	24.18562	13.10548	30.6669	2.58295	56.43704	18.97113	10.1202	6.4156	8.229169	89.55711	8.02611	12.41382
	median	2600.403	2685.143	2635.312	2683.402	2613.933	2727.36	2642.724	2619.611	2613.21	2639.078	2735.45	2638.126	2655.102
	rank	1	10	5	11	3	12	8	4	2	6	13	7	9
C17-F24	mean	2630.488	2765.185	2748.659	2819.559	2630.65	2663.079	2742.631	2676.028	2732.435	2738.514	2731.304	2746.897	2710.324
	best	2516.677	2724.904	2719.254	2798.418	2614.759	2542.972	2716.154	2515.409	2707.696	2724.242	2519.335	2738.729	2553.544
	worst	2732.32	2827.028	2766.535	2875.219	2639.513	2789.346	2771.83	2744.133	2744.878	2750.265	2863.426	2767.252	2788.555
	std	126.7883	51.22319	23.88559	40.45843	12.12815	144.3216	25.01576	117.2769	18.93341	13.46482	161.0712	14.8092	115.5007
	median	2636.477	2754.404	2754.423	2802.3	2634.163	2660	2741.269	2722.285	2738.583	2739.775	2771.229	2740.804	2749.598
	rank	1	12	11	13	2	3	9	4	7	8	6	10	5
C17-F25	mean	2932.639	3107.152	2914.391	3227.178	2918.186	3103.915	2909.269	2921.806	2936.094	2931.645	2921.953	2922.869	2947.757
	best	2898.047	3046.034	2901.119	3168.947	2914.345	2908.676	2786.075	2903.552	2920.869	2915.763	2904.944	2900.751	2934.789
	worst	2945.793	3242.562	2945.862	3291.747	2923.751	3554.229	2952.81	2941.28	2943.151	2948.071	2940.397	2942.787	2957.685
	std	25.12878	100.3049	22.93345	55.32727	4.509969	330.2856	89.45057	22.78116	11.16775	19.53837	20.8472	25.00614	10.61883
	median	2943.359	3070.007	2905.292	3224.008	2917.324	2976.378	2949.096	2921.197	2940.178	2931.373	2921.235	2923.969	2949.278
	rank	8	12	2	13	3	11	1	4	9	7	5	6	10
C17-F26	mean	2900	3479.492	2982.128	3650.998	3009.527	3534.254	3157.14	2913.469	3228.037	3177.481	3741.746	2916.838	2910.943
	best	2900	3232.365	2823.293	3405.694	2892.266	3109.432	2970.468	2899.224	2958.692	2913.788	2823.292	2866.913	2733.158
	worst	2900	3657.094	3168.251	3931.414	3286.061	4083.718	3496.999	2947.122	3814.643	3787.575	4195.301	2993.158	3084.441
	std	4.04E−13	205.1181	200.8003	250.5073	201.5685	505.4077	257.8276	24.52868	429.3952	445.1069	680.2302	58.49097	181.6943
	median	2900	3514.255	2968.485	3633.443	2929.891	3471.932	3080.547	2903.764	3069.407	3004.281	3974.196	2903.641	2913.088
	rank	1	10	5	12	6	11	7	3	9	8	13	4	2
C17-F27	mean	3089.518	3195.072	3117.592	3213.317	3104.402	3168.87	3182.133	3093.149	3114.239	3113.362	3208.927	3131.437	3152.052
	best	3089.518	3152.328	3095.515	3122.317	3092.192	3101.651	3171.92	3090.086	3094.081	3095.582	3197.261	3096.371	3115.537
	worst	3089.518	3264.333	3173.552	3377.978	3132.966	3204.083	3190.778	3094.976	3169.983	3160.563	3226.007	3175.68	3202.016
	std	2.86E−13	52.47827	40.68596	122.7136	20.87492	51.55462	8.446094	2.494371	40.49244	34.32791	13.22445	36.38001	39.34622
	median	3089.518	3181.814	3100.652	3176.487	3096.226	3184.874	3182.916	3093.767	3096.446	3098.651	3206.22	3126.849	3145.328
	rank	1	11	6	13	3	9	10	2	5	4	12	7	8
C17-F28	mean	3100	3538.244	3231.255	3698.548	3216.142	3532.542	3274.874	3233.509	3324.896	3307.813	3415.932	3291.101	3240.069
	best	3100	3501.806	3114.813	3628.394	3165.576	3383.853	3162.006	3108.094	3189.461	3215.18	3407.045	3181.161	3146.602
	worst	3100	3566.356	3357.793	3752.19	3240.529	3713.207	3365.058	3364.619	3385.592	3364.826	3432.454	3357.978	3470.495
	std	0	29.96079	117.1453	60.32794	37.75453	188.0645	111.3176	152.3908	99.02326	75.82069	12.40021	88.80992	168.1178
	median	3100	3542.408	3226.208	3706.803	3229.231	3516.553	3286.217	3230.661	3362.265	3325.623	3412.115	3312.633	3171.588
	rank	1	12	3	13	2	11	6	4	9	8	10	7	5
C17-F29	mean	3132.241	3319.311	3271.941	3350.056	3201.785	3230.335	3327.388	3201.425	3255.274	3210.003	3324.795	3256.025	3231.194
	best	3130.076	3296.748	3203.589	3284.088	3165.3	3167.82	3231.425	3145.081	3188.318	3171.072	3232.237	3166.963	3187.085
	worst	3134.841	3338.025	3339.466	3409.497	3242.517	3286.216	3451.911	3275.176	3358.691	3234.574	3575.403	3325.321	3269.021
	std	2.701544	18.49857	76.50848	71.09305	36.97102	52.816	100.1385	59.0418	88.90214	30.80604	182.3029	77.94277	38.20256
	median	3132.023	3321.236	3272.354	3353.32	3199.661	3233.652	3313.107	3192.721	3237.043	3217.183	3245.769	3265.908	3234.335
	rank	1	10	9	13	3	5	12	2	7	4	11	8	6
C17-F30	mean	3418.734	1,986,627	302,228.1	3,202,386	405,175.5	57,6521.7	900,432	309,219.2	852,117	101,430.5	720,781.7	381,593.1	1,359,456
	best	3394.682	1,492,625	137,971.4	774,684	15,639.03	169,238	61,831.73	8355.747	30,785.93	27,099.55	580,958.5	8815.27	517,597.5
	worst	3442.907	2,767,382	716,581	4,982,029	597,963.4	1,116,490	3,277,457	1,055,327	1,226,511	145,285.1	859,319.6	730,649.2	2,986,435
	std	30.22288	599,846.9	301,967	1,923,438	287,816.6	438,153.6	1,725,459	543,107.4	613,998.1	57,560.13	126,804	430,738.3	1,265,501
	median	3418.673	1,843,250	177,180.1	3,526,415	503,549.7	510,179.4	131,219.7	86,597.28	1,075,585	116,668.6	721,424.3	393,453.9	966,895.8
	rank	1	12	3	13	6	7	10	4	9	2	8	5	11
Sum rank		37	319	178	351	107	287	240	117	189	191	240	184	199
Mean rank		1.275862	11	6.137931	12.10345	3.689655	9.896552	8.275862	4.034483	6.517241	6.586207	8.275862	6.344828	6.862069
Total rank		1	11	4	12	2	10	9	3	6	7	9	5	8

**Table 2:** Optimization results of the CEC 2017 test suite (dimension = 30).

		GAO	WSO	AVOA	RSA	MPA	TSA	WOA	MVO	GWO	TLBO	GSA	PSO	GA
C17-F1	mean	100	2.24E+10	5830.696	3.51E+10	26,029.03	1.53E+10	1.45E+09	462,149.6	1.42E+09	5.27E+09	8,970,393	1.2E+09	1.52E+08
	best	100	1.93E+10	1944.399	3.13E+10	11,980.07	9.61E+09	1.15E+09	358,035.1	2.35E+08	3.33E+09	4300.234	4659.294	1.14E+08
	worst	100	2.81E+10	8674.084	4.32E+10	39,572.94	2.08E+10	1.8E+09	588,039.2	4.29E+09	7.85E+09	31,306,915	4.79E+09	2.1E+08
	std	8.93E−15	4.44E+09	3268.739	5.96E+09	14,512.44	5.72E+09	3.66E+08	123,857.4	2.09E+09	2.06E+09	16,373,797	2.61E+09	45,358,470
	median	100	2.12E+10	6352.15	3.3E+10	26,281.55	1.54E+10	1.43E+09	451,262.1	5.87E+08	4.94E+09	2,285,178	2,732,577	1.43E+08
	rank	1	12	2	13	3	11	9	4	8	10	5	7	6
C17-F3	mean	300	83,231.63	38,295.6	62,966.84	1080.016	40,427.18	198,100.3	1654.42	35,704.8	29,742.72	81,960.49	27,361.07	142,883.2
	best	300	75,990.45	20,886.01	48,763.59	835.6308	38,295.97	163,903.7	1359.797	31,222.91	25,372.64	70,568.25	19,591.81	108,167.1
	worst	300	91,401.08	49,468.55	68,418.5	1327.762	42,603.54	227,591	2234.118	39,845.54	32,187.24	90,214.39	35,088.1	198,501.9
	std	0	8274.729	13,338.25	10,339.67	240.5065	2328.613	28,830.94	435.0825	3847.771	3343.804	9653.533	7695.205	46,670.58
	median	300	82,767.5	41,413.91	67,342.64	1078.334	40,404.6	200,453.3	1511.882	35,875.37	30,705.5	83,529.66	27,382.18	132,431.9
	rank	1	11	7	9	2	8	13	3	6	5	10	4	12
C17-F4	mean	458.5616	5575.746	510.5674	8459.244	492.1852	3951.847	802.8511	495.2865	559.4071	846.3066	578.9619	604.0431	764.5042
	best	458.5616	3162.415	490.1107	5447.077	481.9965	964.989	746.8999	487.3344	514.4662	669.0919	560.641	511.0539	719.023
	worst	458.5616	7521.568	525.685	11,795.17	513.3417	6521.647	870.971	509.3757	584.941	1190.549	598.5419	764.1292	785.5119
	std	0	1966.289	16.4318	2867.916	15.6659	2553.534	60.90524	10.60051	33.63155	254.441	17.40204	125.6863	33.99235
	median	458.5616	5809.5	513.237	8297.365	486.7013	4160.375	796.7667	492.218	569.1106	762.7929	578.3323	570.4947	776.7408
	rank	1	12	4	13	2	11	9	3	5	10	6	7	8
C17-F5	mean	502.4874	804.9869	702.7953	838.5171	581.1528	761.4417	786.479	612.1267	614.2579	741.2772	700.5764	623.3892	683.0927
	best	500.995	787.2262	670.5593	815.5432	559.181	736.3111	758.9541	602.1272	582.3474	719.2387	684.4747	605.1798	643.8764
	worst	503.9798	820.5739	755.7975	865.0304	603.5573	787.5596	801.0327	639.4914	638.7938	766.5766	722.3585	665.0013	737.5901
	std	1.397909	15.42534	41.61819	26.24631	20.23733	25.92504	20.39522	19.87255	30.24951	23.8542	17.75002	30.70363	42.73087
	median	502.4874	806.0737	692.4122	836.7474	580.9364	760.9481	792.9647	603.4442	617.9452	739.6468	697.7363	611.6878	675.4521
	rank	1	12	8	13	2	10	11	3	4	9	7	5	6
C17-F6	mean	600	668.4481	640.1894	671.1451	603.1735	666.0123	665.3591	621.1771	610.5213	637.3057	648.544	640.3844	626.1082
	best	600	667.3664	638.4326	666.4683	601.939	652.8759	656.1085	611.1992	604.3109	631.0881	647.7581	629.852	620.0473
	worst	600	669.7374	642.8848	676.7635	604.5364	673.7459	669.9162	631.8469	616.418	647.1292	649.5544	649.4784	630.0802
	std	7.14E−14	1.066492	2.110469	5.171545	1.228254	10.61633	6.893277	10.64413	5.433745	7.617645	0.830897	9.48126	4.784344
	median	600	668.3442	639.7201	670.6744	603.1092	668.7137	667.7058	620.8312	610.6782	635.5028	648.4318	641.1036	627.1526
	rank	1	12	7	13	2	11	10	4	3	6	9	8	5
C17-F7	mean	733.478	1228.995	1099.712	1263.799	843.951	1165.878	1236.662	850.2233	877.2289	1039.196	950.1445	870.9795	946.9537
	best	732.8186	1186.202	1007.089	1248.902	817.6154	1039.596	1201.701	801.3773	815.1492	970.212	916.1667	849.6304	909.8194
	worst	734.5199	1259.268	1234.888	1290.136	896.3808	1293.007	1302.601	911.4905	908.4492	1103.662	1008.406	892.354	1000.135
	std	0.820605	36.18977	110.598	19.99462	38.76962	121.0686	50.70099	51.45941	45.79175	77.02059	45.68643	19.11727	41.38543
	median	733.2867	1235.255	1078.436	1258.078	830.9039	1165.454	1221.173	844.0127	892.6586	1041.454	938.0026	870.9668	938.93
	rank	1	11	9	13	2	10	12	3	5	8	7	4	6
C17-F8	mean	803.3298	1054.375	939.0289	1086.282	888.6842	1032.224	1007.835	891.0273	889.8869	1001.196	949.1516	916.8257	970.2233
	best	801.2023	1042.296	914.1188	1068.901	882.1376	995.1895	958.6942	864.7594	883.8931	984.9519	929.263	906.7325	955.6456
	worst	804.1574	1071.703	957.41	1109.377	896.6531	1121.21	1044.586	915.7834	896.691	1030.481	971.32	930.3388	987.7654
	std	1.546288	15.01989	21.53442	22.05067	6.526089	65.10001	39.86739	24.5518	5.840703	21.82905	20.35337	11.17469	17.18677
	median	803.9798	1051.751	942.2934	1083.424	887.9731	1006.249	1014.029	891.7831	889.4817	994.6756	948.0116	915.1158	968.7411
	rank	1	12	6	13	2	11	10	4	3	9	7	5	8
C17-F9	mean	900	9515.324	4272.211	9225.632	1079.736	9962.829	9568.298	4810.704	1927.55	5082.281	3636.606	3182.606	1257.708
	best	900	8134.16	3187.446	9011.84	928.9729	6111.314	7340.205	3844.345	1477.303	3695.543	3195.715	1970.943	1094.368
	worst	900	10,808.32	4864.822	9356.939	1228.385	13,414.13	11,390.81	7265.409	2581.598	7613.125	4353.529	4753.266	1447.598
	std	7.14E−14	1212.907	814.2805	161.866	150.6155	3281.801	2223.351	1786.248	580.2782	1928.028	561.7954	1287.229	184.2518
	median	900	9559.408	4518.287	9266.875	1080.793	10,162.93	9771.089	4066.531	1825.65	4510.228	3498.591	3003.108	1244.433
	rank	1	11	7	10	2	13	12	8	4	9	6	5	3
C17-F10	mean	2293.267	6711.466	5199.584	7297.233	3948.269	6147.451	6093.07	4512.635	4631.659	7314.039	4682.42	4846.696	5790.657
	best	1851.756	6257.175	4542.675	6605.22	3601.53	4997.117	5353.014	4231.179	4212.362	6960.446	4431.502	4657.101	5397.511
	worst	2525.027	7003.829	5653.819	7857.574	4375.692	6681.464	7277.149	4817.147	4957.496	7486.531	5057.283	5215.581	6319.118
	std	326.8979	348.455	562.337	566.1807	388.8296	843.4916	936.7337	300.4517	340.3693	261.3508	301.6935	272.724	472.6668
	median	2398.142	6792.43	5300.922	7363.07	3907.926	6455.611	5871.057	4501.106	4678.389	7404.589	4620.448	4757.052	5723.001
	rank	1	11	7	12	2	10	9	3	4	13	5	6	8
C17-F11	mean	1102.987	6588.648	1243.679	7700.281	1168.172	4557.763	6855.893	1291.932	2045.681	1868.273	2647.571	1236.428	8014.356
	best	1100.995	5445.541	1190.234	6294.032	1121.752	3286.896	4977.714	1249.106	1356.107	1531.097	2080.833	1210.801	3047.23
	worst	1105.977	7524.423	1292.976	8649.705	1200.997	6797.836	10,068.8	1331.2	3882.607	2497.356	3226.279	1262.525	14,907.23
	std	2.342568	993.8017	46.63589	1174.213	37.26024	1718.502	2416.292	48.66841	1334.469	466.8468	586.8497	24.95497	5531.35
	median	1102.487	6692.315	1245.752	7928.694	1174.97	4073.161	6188.529	1293.712	1472.005	1722.319	2641.587	1236.193	7051.482
	rank	1	10	4	12	2	9	11	5	7	6	8	3	13
C17-F12	mean	1744.553	6.02E+09	17,863,386	9.34E+09	21,141.86	4.34E+09	2.12E+08	9,618,730	45,007,811	2.59E+08	1.71E+08	2,197,963	6,585,298
	best	1721.81	4.97E+09	2,515,715	8.33E+09	15,112.24	2.24E+09	54,247,868	4,467,693	4,371,684	1.65E+08	32,963,915	240,110	4,560,763
	worst	1764.937	7.64E+09	43,624,026	1.18E+10	26,966.2	5.68E+09	4.23E+08	23,269,434	94,380,138	4.49E+08	5.45E+08	4,367,235	8,619,582
	std	21.9323	1.24E+09	19,693,155	1.77E+09	5497.114	1.62E+09	1.85E+08	9,919,151	42,706,241	1.4E+08	2.72E+08	1,937,243	2,003,322
	median	1745.733	5.73E+09	12,656,901	8.64E+09	21,244.5	4.73E+09	1.85E+08	5,368,897	40,639,712	2.1E+08	52,257,988	2,092,253	6,580,423
	rank	1	12	6	13	2	11	9	5	7	10	8	3	4
C17-F13	mean	1315.791	4.89E+09	128,357.7	9.03E+09	1875.226	1.25E+09	774,665.8	78,141.05	646,635.7	75,500,364	31,494.84	27,975.88	10,201,641
	best	1314.587	2.38E+09	71,240.8	4.74E+09	1607.384	16,892,010	365,628.3	31,432.53	78,361.26	52,431,398	25,563.03	11,712.82	2,768,082
	worst	1318.646	6.85E+09	202,845.2	1.11E+10	2399.919	4.35E+09	1,145,245	156,691.7	2,006,033	1.11E+08	46,031.26	62,954.17	21,943,429
	std	2.107258	2.01E+09	59,484.26	3.16E+09	389.8455	2.27E+09	442,347.8	63,996.54	999,080.8	27,735,110	10,663.11	25,672.39	8,943,170
	median	1314.967	5.17E+09	119,672.5	1.01E+10	1746.801	3.23E+08	793,895	62,219.97	251,074.4	69,119,457	27,192.53	18,618.27	8,047,527
	rank	1	12	6	13	2	11	8	5	7	10	4	3	9
C17-F14	mean	1423.017	1,620,883	232,138.6	1,878,343	1439.96	1,004,611	1,901,641	17,597.69	456,074.8	119,821	978,295.3	16,252.17	1,716,822
	best	1422.014	999,618.1	32,641.8	944,177.7	1436.666	718,913.2	30,911.57	4476.323	29,593.77	69,721.47	634,875.7	2922.639	284,338.6
	worst	1423.993	2,051,782	537,168.9	2,796,941	1444.619	1,419,165	5,808,995	29,816.42	977,253.7	137,833.3	1,476,990	29,507.03	2,894,290
	std	0.87954	535,959.6	242,266.2	969,969.9	3.964006	349,745.9	2,887,685	11,878.97	523,579.8	36,363.11	431,297.5	12,638.36	1,310,027
	median	1423.03	1,716,065	179,371.8	1,886,126	1439.277	940,183.2	883,327.9	18,049.02	408,725.8	135,864.6	900,657.9	16,289.51	1,844,330
	rank	1	10	6	12	2	9	13	4	7	5	8	3	11
C17-F15	mean	1503.129	2.6E+08	32,238.74	5.11E+08	1615.842	12,285,162	4,311,392	36,795.69	13,526,091	4,387,752	13,965.85	4316.719	816,946.7
	best	1502.462	2.25E+08	9568.865	4.41E+08	1579.303	4,839,717	198,819.8	21,398.4	84,202.47	996,467.3	9982.633	1863.552	150,141
	worst	1504.265	2.88E+08	52,213.63	5.64E+08	1632.203	28,577,478	13,998,112	60,719.59	50,643,212	8,259,350	18,859.25	7827.492	1,830,094
	std	0.931104	33,963,783	19,599.84	65,714,951	26.71831	11,924,974	7,123,977	18,547.3	26,943,033	3,240,985	4038.215	2873.981	836,631.2
	median	1502.893	2.64E+08	33,586.24	5.19E+08	1625.931	7,861,726	1,524,317	32,532.39	1,688,475	4,147,596	13,510.76	3787.916	643,775.8
	rank	1	12	5	13	2	10	8	6	11	9	4	3	7
C17-F16	mean	1663.469	3976.13	2850.193	4538.035	2018.075	3081.824	3912.824	2497.509	2459.731	3246.375	3418.88	2790.369	2806.553
	best	1614.72	3677.136	2498.276	3857.04	1729.78	2684.641	3280.597	2281.547	2296.23	3046.042	3224.039	2566.73	2476.202
	worst	1744.118	4228.361	3313.751	5153.785	2265.618	3317.831	4670.899	2689.547	2594.452	3477.372	3554.178	3046.454	3136.355
	std	67.44425	260.6856	370.1947	729.5955	262.3233	301.4003	627.3598	194.6654	161.2045	207.3804	160.5896	243.2084	346.6029
	median	1647.519	3999.511	2794.373	4570.657	2038.452	3162.412	3849.901	2509.47	2474.121	3231.043	3448.651	2774.146	2806.828
	rank	1	12	7	13	2	8	11	4	3	9	10	5	6
C17-F17	mean	1728.099	3184.02	2385.059	3444.454	1861.554	3068.849	2705.694	2048.793	1922.204	2146.088	2427.883	2266.784	2113.355
	best	1718.761	2670.362	2263.79	3120.014	1753.291	2170.082	2296.654	1992.226	1798.016	1954.46	2328.457	2067.577	2068.723
	worst	1733.659	3809.326	2471.165	4025.836	1921.879	5414.867	2971.642	2181.755	2057.325	2387.679	2561.329	2612.166	2177.383
	std	7.30039	526.9921	100.2765	449.8941	80.94562	1704.675	316.43	97.00013	131.8202	199.0191	120.9462	266.3502	53.95218
	median	1729.987	3128.196	2402.64	3315.983	1885.523	2345.223	2777.24	2010.594	1916.738	2121.107	2410.873	2193.697	2103.656
	rank	1	12	8	13	2	11	10	4	3	6	9	7	5
C17-F18	mean	1825.696	24,287,534	2,263,992	27,925,543	1895.059	31,053,715	5,043,241	547,132.2	358,761.7	1,423,873	440,293.8	117,519	3,115,606
	best	1822.524	6,996,614	241,331.5	9,028,538	1873.011	1,138,972	1,699,708	137,877.9	67,292.89	661,159.2	246,961.1	83,697.64	2,432,402
	worst	1828.42	47,167,452	4,516,915	54,862,462	1907.868	58,848,115	10,408,932	1,480,690	921,379.6	1,790,003	857,003.1	139,389	4,566,779
	std	2.940513	19,326,659	2,180,757	21,151,997	17.03125	34,876,427	4,073,042	681,637.6	437,442.1	564,848.5	306,279.1	26,498.85	1,064,963
	median	1825.92	21,493,035	2,148,860	23,905,585	1899.679	32,113,887	4,032,162	284,980.2	223,187.3	1,622,165	328,605.4	123,494.6	2,731,622
	rank	1	11	8	12	2	13	10	6	4	7	5	3	9
C17-F19	mean	1910.989	4.96E+08	58,126.47	8.37E+08	1923.509	2.52E+08	12,243,666	803,041	3,446,841	4,915,042	70,150.44	38,301.8	1,385,589
	best	1908.84	3.71E+08	12,610.39	6.04E+08	1920.961	3,125,025	1,593,445	20,530.54	60,786.57	2,551,343	38,175.1	7765.051	547,648.4
	worst	1913.095	6.46E+08	129,139.1	1.27E+09	1928.282	6.97E+08	21,141,224	1,805,173	11,114,255	6,986,559	94,307.98	114,161.1	2,461,289
	std	2.10261	1.5E+08	55,242.46	3.2E+08	3.557655	3.49E+08	9,701,357	945,184	5,600,908	2,374,247	25,431.89	55,229.08	878,355.7
	median	1911.01	4.84E+08	45,378.18	7.37E+08	1922.396	1.53E+08	13,119,997	693,230.5	1,306,161	5,061,133	74,059.34	15,640.51	1,266,709
	rank	1	12	4	13	2	11	10	6	8	9	5	3	7
C17-F20	mean	2065.787	2796.248	2569.721	2842.618	2174.505	2754.131	2743.498	2543.502	2345.481	2709.577	2891.383	2493.68	2431.832
	best	2029.521	2717.077	2435.499	2688.781	2060.52	2632.945	2579.927	2330.185	2181.405	2644.37	2572.029	2447.954	2375.804
	worst	2161.126	2880.76	2758.187	2923.577	2263.092	2870.725	2902.416	2905.285	2492.129	2815.418	3316.121	2610.098	2471.322
	std	69.26656	72.88532	151.0366	116.0351	92.04375	106.7938	148.3052	272.6655	138.6492	86.75523	340.1559	84.99863	44.58253
	median	2036.25	2793.577	2542.599	2879.058	2187.204	2756.427	2745.823	2469.269	2354.195	2689.26	2838.691	2458.335	2440.101
	rank	1	11	7	12	2	10	9	6	3	8	13	5	4
C17-F21	mean	2308.456	2586.112	2429.733	2635.523	2365.407	2510.732	2575.654	2398.876	2385.776	2476.648	2541.079	2424.19	2474.167
	best	2304.034	2503.828	2239.133	2566.699	2355.832	2313.975	2511.033	2367.311	2357.335	2464.956	2524.504	2406.825	2444.909
	worst	2312.987	2639.649	2565.011	2717.666	2381.228	2626.13	2631.429	2423.678	2398.434	2485.83	2571.965	2437.464	2519.797
	std	4.852783	70.34927	149.177	71.48777	12.14978	150.3418	65.13148	25.54739	21.14649	11.60673	22.98087	15.86086	34.85109
	median	2308.402	2600.486	2457.394	2628.864	2362.283	2551.411	2580.077	2402.257	2393.667	2477.902	2533.924	2426.235	2465.981
	rank	1	12	6	13	2	9	11	4	3	8	10	5	7
C17-F22	mean	2300	7197.735	5291.965	6988.536	2302.872	7878.421	6699.701	3725.871	2648.451	5216.932	5770.482	4527.268	2646.874
	best	2300	6905.286	2302.895	6100.795	2301.873	7679.199	5875.195	2305.955	2536.943	2665.425	3766.123	2436.13	2582.523
	worst	2300	7653.951	6446.791	7880.646	2304.558	7972.315	7435.194	5489.079	2877.381	8045.799	6651.321	6550.838	2696.642
	std	0	348.1898	2172.161	832.4763	1.310487	149.9847	705.559	1809.434	169.3872	3188.216	1463.762	2059.341	61.57958
	median	2300	7115.851	6209.087	6986.352	2302.528	7931.085	6744.208	3554.226	2589.739	5078.252	6332.241	4561.053	2654.166
	rank	1	12	8	11	2	13	10	5	4	7	9	6	3
C17-F23	mean	2655.081	3119.093	2889.696	3166.646	2646.19	3123.314	2993.881	2725.509	2737.411	2869.741	3618.178	2866.813	2931.68
	best	2653.745	3052.69	2774.059	3125.186	2474.16	3028.342	2848.34	2694.373	2710.109	2830.903	3532.397	2816.976	2885.392
	worst	2657.377	3197.28	3047.49	3213.919	2711.793	3301.686	3087.77	2740.845	2761.013	2920.587	3703.143	2919.204	2994.691
	std	1.79918	69.32291	126.5441	41.69167	125.1344	132.9871	111.362	23.13598	24.97449	41.08474	98.87545	47.85622	49.70222
	median	2654.6	3113.202	2868.617	3163.74	2699.404	3081.615	3019.707	2733.409	2739.261	2863.737	3618.585	2865.536	2923.318
	rank	2	10	7	12	1	11	9	3	4	6	13	5	8
C17-F24	mean	2831.409	3257.436	3132.252	3344.225	2882.957	3227.736	3085.012	2902.245	2915.402	3020.71	3299.939	3097.741	3180.377
	best	2829.992	3222.637	3012.473	3264.802	2867.506	3133.14	3030.05	2861.253	2905.338	2998.261	3265.898	3029.904	3098.613
	worst	2832.366	3326.504	3265.163	3479.85	2889.738	3273.031	3108.048	2920.182	2922.286	3053.136	3333.143	3197.142	3247.25
	std	1.246718	50.93461	120.9993	107.6055	11.36076	70.91674	40.20797	29.98429	8.273703	25.17521	32.17397	77.64043	75.42338
	median	2831.64	3240.301	3125.686	3316.123	2887.292	3252.387	3100.975	2913.773	2916.991	3015.721	3300.357	3081.959	3187.823
	rank	1	11	8	13	2	10	6	3	4	5	12	7	9
C17-F25	mean	2886.698	3800.088	2906.312	4342.239	2891.222	3392.322	3056.359	2907.023	2979.636	3050.164	2981.487	2894.406	3078.72
	best	2886.691	3473.964	2893.165	3818.698	2884.561	3063.681	3023.9	2886.114	2945.826	2945.791	2970.645	2887.224	3063.826
	worst	2886.707	4043.769	2939.912	5039.77	2897.338	3732.374	3073.082	2961.847	3041.478	3168.502	2993.113	2910.1	3089.885
	std	0.008278	258.9364	24.42658	553.4353	6.284407	355.6964	25.12833	39.88302	48.18031	116.3059	10.12724	11.48927	12.25239
	median	2886.698	3841.309	2896.085	4255.243	2891.495	3386.617	3064.226	2890.065	2965.62	3043.182	2981.095	2890.151	3080.585
	rank	1	12	4	13	2	11	9	5	6	8	7	3	10
C17-F26	mean	3578.65	8348.562	6747.9	8857.441	2959.894	7963.37	7654.154	4548.82	4356.689	5532.353	6873.735	4601.941	4208.71
	best	3559.841	7978.641	5633.769	8131.148	2958.088	7389.984	7017.352	4248.451	4014.143	4334.846	5962.246	3474.557	3875.117
	worst	3607.686	9012.359	7403.174	10,142.89	2962.551	8325.199	8394.322	5096.284	4887.213	6669.677	7348.178	5942.959	4617.04
	std	24.78775	524.3091	846.9994	1027.892	2.329347	436.4335	615.4601	428.6104	405.4909	1164.857	703.2545	1254.391	338.6951
	median	3573.536	8201.625	6977.328	8577.864	2959.47	8069.148	7602.471	4425.273	4262.7	5562.445	7092.259	4495.123	4171.342
	rank	2	12	8	13	1	11	10	5	4	7	9	6	3
C17-F27	mean	3207.018	3557.859	3336.614	3692.551	3214.516	3439.143	3399.203	3229.081	3245.065	3304.138	4738.265	3270.064	3427.096
	best	3200.749	3504.742	3259.845	3447.597	3200.701	3325.41	3252.487	3213.263	3239.732	3239.46	4345.466	3234.937	3360.528
	worst	3210.656	3647.653	3401.068	3944.515	3234.537	3654.379	3507.364	3250.664	3256.4	3367.534	5025.606	3307.487	3464.856
	std	5.058229	69.40237	81.40933	231.9974	16.86964	160.4216	118.7568	17.15178	8.322647	57.63497	362.1397	33.46625	49.7926
	median	3208.335	3539.521	3342.771	3689.047	3211.413	3388.391	3418.481	3226.198	3242.063	3304.78	4790.994	3268.916	3441.501
	rank	1	11	7	12	2	10	8	3	4	6	13	5	9
C17-F28	mean	3100	4571.047	3257.514	5360.196	3212.502	4031.57	3407.388	3249.529	3545.039	3607.884	3479.289	3312.468	3532.676
	best	3100	4364.764	3228.668	5089.878	3196.105	3546.402	3354.006	3217.768	3371.526	3476.923	3416.993	3195.173	3485.352
	worst	3100	4796.882	3289.077	5645.616	3242.413	4524.153	3454.018	3276.914	3966.099	3904.816	3607.994	3495.259	3585.531
	std	2.86E−13	201.1901	26.93721	288.6574	22.52248	493.922	48.42336	26.91766	307.589	218.2223	94.7696	151.3715	51.32503
	median	3100	4561.271	3256.155	5352.645	3205.744	4027.863	3410.765	3251.717	3421.266	3524.899	3446.085	3279.72	3529.911
	rank	1	12	4	13	2	11	6	3	9	10	7	5	8
C17-F29	mean	3353.75	5165.165	4238.483	5356.041	3653.907	5027.779	4894.057	3814.832	3769.346	4394.082	4873.204	4097.12	4200.257
	best	3325.385	4781.032	3925.131	4815.572	3502.249	4549.384	4640.711	3697.589	3678.852	4101.821	4617.358	3940.75	3860.919
	worst	3370.797	5597.427	4432.861	6103.185	3791.683	5814.268	5060.487	3902.269	3867.5	4822.173	5105.334	4318.126	4492.071
	std	21.42746	430.9386	247.3722	699.807	139.048	639.3948	194.8489	97.52882	87.40834	336.9599	272.1672	172.6724	311.0435
	median	3359.41	5141.101	4297.97	5252.703	3660.848	4873.732	4937.515	3829.735	3765.517	4326.167	4885.062	4064.802	4224.02
	rank	1	12	7	13	2	11	10	4	3	8	9	5	6
C17-F30	mean	5007.854	1.23E+09	1,226,561	2.43E+09	7628.457	33,024,519	33,699,582	2,659,152	5,482,512	32,533,736	1,945,470	235,225.1	604,275.2
	best	4955.449	9.06E+08	433,022.5	1.74E+09	6346.969	11,291,100	6,721,212	478,549.6	1,223,756	17,415,442	1,698,234	7391.654	167,926.1
	worst	5086.396	1.35E+09	2,171,246	2.68E+09	10,132.67	77,162,996	54,000,073	3,806,634	14,803,056	68,241,237	2,340,871	887,651.6	1,155,344
	std	64.18196	2.36E+08	790,932.4	4.97E+08	1930.5	32,540,981	21,448,199	1,615,422	6,825,432	26,053,037	301,100	473,587.2	523,179.4
	median	4994.785	1.33E+09	1,150,987	2.64E+09	7017.094	21,821,990	37,038,521	3,175,713	2,951,619	22,239,133	1,871,388	22,928.54	546,915.6
	rank	1	12	5	13	2	10	11	7	8	9	6	3	4
Sum rank		31	334	182	361	57	305	284	128	151	232	231	139	204
Mean rank		1.068966	11.51724	6.275862	12.44828	1.965517	10.51724	9.793103	4.413793	5.206897	8	7.965517	4.793103	7.034483
Total rank		1	12	6	13	2	11	10	3	5	9	8	4	7

**Table 3:** Optimization results of the CEC 2017 test suite (dimension = 50).

		GAO	WSO	AVOA	RSA	MPA	TSA	WOA	MVO	GWO	TLBO	GSA	PSO	GA
C17-F1	mean	100	5.09E+10	8,540,548	7.98E+10	5,463,321	3.24E+10	6.56E+09	4,128,914	7.97E+09	1.77E+10	1.46E+10	2.16E+09	8.86E+09
	best	100	4.55E+10	1,215,225	6.98E+10	2,108,552	2.99E+10	3.87E+09	2,756,506	5.74E+09	1.2E+10	1.16E+10	8.85E+08	8.44E+09
	worst	100	5.45E+10	21,279,391	8.72E+10	13,852,712	3.49E+10	9.82E+09	5,284,968	1.09E+10	2.39E+10	1.75E+10	2.88E+09	9.54E+09
	std	0	4.35E+09	9,547,461	8.27E+09	6,130,610	2.27E+09	3.06E+09	1,169,138	2.34E+09	6.24E+09	2.59E+09	9.53E+08	5.65E+08
	median	100	5.19E+10	5,833,788	8.11E+10	2,946,011	3.25E+10	6.28E+09	4,237,091	7.61E+09	1.74E+10	1.46E+10	2.44E+09	8.73E+09
	rank	1	12	4	13	3	11	6	2	7	10	9	5	8
C17-F3	mean	300	137,010.9	126,882.3	136,511.5	17,391.76	95,002.13	201,272.3	41,494.88	112,747.5	85,794.71	153,610.6	125,333.9	226,348.9
	best	300	117,906.7	97,696.29	124,125.1	15,025.04	83,816.75	152,134.7	33,432.18	99,401.38	65,216.63	139,017.6	94,437.17	188,829.6
	worst	300	157,634.1	153,730.6	148,328	20,524.62	101,529.7	306,297.4	51,471.59	126,680.5	97,304.28	172,990.6	162,490.2	259,863.3
	std	0	18,198.32	274,92.83	11,628.69	2686.645	8787.683	78,962.98	8212.567	12,134.77	16,021.99	18,038.98	32,119.68	31,660.35
	median	300	136,251.3	128,051.1	136,796.5	17,008.7	97,331.05	173,328.5	40,537.89	112,454.1	90,328.97	151,217	122,204	228,351.4
	rank	1	10	8	9	2	5	12	3	6	4	11	7	13
C17-F4	mean	470.3679	12,640.05	670.9189	20,297.76	529.2201	7151.718	1728.284	556.4852	1299.368	2455.851	2684.969	941.9422	1375.405
	best	428.5127	9836.365	654.6081	13,423.73	493.8869	5740.654	1128.951	520.5781	981.7953	1417.507	2250.684	654.6616	1194.41
	worst	525.7252	14,388.37	689.3209	24,235.9	581.4109	9217.082	2051.556	618.0226	1569.432	4151.915	2849.68	1617.781	1483.827
	std	53.9489	2214.027	18.01175	5368.95	44.6304	1598.366	449.3558	46.63019	289.3042	1307.92	316.5375	492.8826	138.0845
	median	463.6168	13,167.74	669.8733	21,765.71	520.7913	6824.568	1866.314	543.6699	1323.123	2126.992	2819.756	747.663	1411.692
	rank	1	12	4	13	2	11	8	3	6	9	10	5	7
C17-F5	mean	504.7261	1037.655	832.0331	1062.51	728.5695	1077.7	915.4502	730.7567	719.4511	951.9034	788.0505	773.5328	860.9533
	best	503.9798	1009.534	796.5431	1053.971	649.5934	956.2157	883.3966	658.6571	686.5838	906.6592	733.7376	717.5713	825.7998
	worst	505.9698	1062.384	874.7422	1070.73	790.4663	1164.931	937.424	834.3487	751.0828	977.7863	821.9891	830.6777	884.0127
	std	1.036717	27.61624	36.05762	7.545912	64.31447	111.7325	28.62198	84.28709	34.43552	34.26955	45.11241	50.26869	28.8839
	median	504.4773	1039.35	828.4235	1062.669	737.1091	1094.826	920.4901	715.0105	720.069	961.584	798.2378	772.9411	867.0004
	rank	1	11	7	12	3	13	9	4	2	10	6	5	8
C17-F6	mean	600	681.9603	652.3174	683.7161	610.9482	677.4992	684.2625	633.2341	620.654	655.7374	650.412	646.7268	642.4574
	best	600	679.4617	648.6703	681.4595	608.2624	660.2885	679.3851	624.4301	615.8844	644.814	645.9264	644.6453	631.948
	worst	600	686.6001	656.7956	686.2413	614.4981	692.0708	691.2724	653.6353	628.6895	663.5099	653.3149	649.7848	652.834
	std	0	3.618989	4.092498	2.496933	2.906614	15.27414	5.509322	15.03081	6.167687	8.616449	3.484165	2.621147	9.547808
	median	600	680.8897	651.9018	683.5817	610.5162	678.8188	683.1963	627.4354	619.021	657.3128	651.2033	646.2385	642.5239
	rank	1	11	8	12	2	10	13	4	3	9	7	6	5
C17-F7	mean	756.7298	1667.515	1560.123	1752.153	1019.514	1574.269	1595.787	1040.812	1050.837	1401.142	1343.727	1164.307	1255.661
	best	754.7543	1651.905	1504.114	1689.052	964.5631	1449.444	1546.801	1005.244	1024.421	1289.8	1193.977	1033.213	1194.715
	worst	758.3522	1687.845	1622.18	1844.419	1066.022	1694.582	1666.003	1072.362	1072.624	1446.958	1459.1	1355.126	1292.502
	std	1.69049	16.27905	54.23613	74.30205	53.48986	127.8245	59.26941	29.9404	24.75606	81.06107	127.8115	152.3713	47.12675
	median	756.9065	1665.154	1557.099	1737.571	1023.735	1576.525	1585.172	1042.82	1053.152	1433.904	1360.916	1134.445	1267.713
	rank	1	12	9	13	2	10	11	3	4	8	7	5	6
C17-F8	mean	805.721	1351.115	1100.231	1374.561	1003.671	1365.881	1270.348	1013.356	1023.541	1268.178	1113.789	1042.706	1213.494
	best	802.9849	1306.313	1057.214	1351.374	973.8483	1277.833	1160.6	982.3324	990.5358	1217.312	1103.608	1002.871	1180.728
	worst	810.9445	1385.419	1143.508	1389.617	1034.27	1477.047	1366.566	1070.762	1059.941	1318.566	1129.003	1102.533	1230.719
	std	3.891615	40.62085	53.30554	17.89982	34.19462	92.61518	92.03362	42.74163	34.1913	45.15074	12.91415	49.50142	24.50797
	median	804.4773	1356.363	1100.101	1378.627	1003.282	1354.322	1277.113	1000.165	1021.844	1268.417	1111.273	1032.71	1221.264
	rank	1	11	6	13	2	12	10	3	4	9	7	5	8
C17-F9	mean	900	31,051.1	11,675.13	31,214.4	3238.679	32,551.61	28,392.1	17,082.88	6244.482	20,791.08	9460.756	9138.502	11,261.84
	best	900	29,810.85	11,146.57	29,323.19	2032.095	29,890.05	26,453.7	9511.032	5336.41	15,961.04	8656.557	8548.842	9310.568
	worst	900	34,048.73	12,380.93	32,909.38	4682.586	36,436.44	33,315.56	22,458.82	7025.394	24,361.63	10,359.38	10,329.21	13,039.73
	std	1.01E−13	2195.966	559.63	1783.168	1191.598	3056.232	3579.538	6649.355	980.5268	3819.075	778.1417	888.7583	2162.608
	median	900	30,172.41	11,586.52	31,312.5	3120.018	31,939.98	26,899.57	18,180.84	6308.061	21,420.81	9413.544	8837.979	11,348.54
	rank	1	11	7	12	2	13	10	8	3	9	5	4	6
C17-F10	mean	4347.157	11,960.59	7997.318	12,995.76	6477.477	10,922.78	10,929.36	7432.631	8285.742	12,815.7	8228.953	7542.419	10,862.63
	best	3555.132	11,511.48	7500.439	12,777.04	5611.471	10,052.08	9913.249	6365.31	6430.52	12,259.66	7464.218	7304.678	10,276.37
	worst	5099.795	12,681.4	8505.039	13,415.44	7088.816	11,917.98	11,991.42	8254.136	12,660.49	13,271.59	9251.954	7955.953	11,437.09
	std	701.6898	588.0922	446.6302	320.7599	774.7588	852.148	980.4482	881.348	3204.405	539.0572	824.0267	309.7522	536.1431
	median	4366.851	11,824.73	7991.897	12,895.29	6604.81	10,860.52	10,906.38	7555.538	7025.978	12,865.77	8099.819	7454.522	10,868.54
	rank	1	11	5	13	2	9	10	3	7	12	6	4	8
C17-F11	mean	1128.435	13,276.15	1549.54	18,035.48	1251.306	11,182.39	4518.476	1518.213	5397.474	4531.994	12,260.42	1605.813	20,608.14
	best	1121.25	12,245.45	1447.083	16,062.88	1204.288	9640.254	4005.969	1391.868	3312.855	4260.571	11,504.42	1377.528	12,119.73
	worst	1133.132	13,924.57	1673.984	19,534.31	1281.377	13,383.07	5612.842	1642.615	9241.98	5027.755	13,871.78	1877.551	27,569.61
	std	5.923599	809.4648	113.8746	1578.695	37.32774	1755.825	805.482	119.8359	2981.794	385.6477	1182.421	233.5491	6954.273
	median	1129.678	13,467.3	1538.547	18,272.37	1259.779	10,853.12	4227.547	1519.185	4517.532	4419.825	11,832.75	1584.087	21,371.61
	rank	1	11	4	12	2	9	6	3	8	7	10	5	13
C17-F12	mean	2905.102	3.72E+10	64,228,735	6.06E+10	13,971,168	2.2E+10	1.13E+09	69,199,513	8.18E+08	4.31E+09	1.85E+09	1.37E+09	1.76E+08
	best	2527.376	3.12E+10	28,088,258	4.42E+10	13,160,600	9.3E+09	9.31E+08	38,018,157	1.3E+08	2.43E+09	6.11E+08	12,579,081	56,622,701
	worst	3168.37	4.46E+10	98,280,368	8.32E+10	14,626,284	3.71E+10	1.53E+09	1.09E+08	1.52E+09	8.47E+09	3.33E+09	3.95E+09	2.43E+08
	std	297.8769	6.56E+09	40,914,989	1.95E+10	74,4618.4	1.25E+10	3.02E+08	32,549,765	7.54E+08	3.08E+09	1.22E+09	2E+09	89,011,221
	median	2962.331	3.64E+10	65,273,157	5.76E+10	14,048,895	2.09E+10	1.02E+09	64,796,411	8.12E+08	3.16E+09	1.74E+09	7.55E+08	2.02E+08
	rank	1	12	3	13	2	11	7	4	6	10	9	8	5
C17-F13	mean	1340.1	2.1E+10	12,8969.3	3.67E+10	15,885.29	8.59E+09	80,889,215	20,7549.2	3.04E+08	4.99E+08	15,793,658	4.07E+08	35,383,568
	best	1333.781	1.21E+10	31,511.27	1.86E+10	8423.568	4.57E+09	60,813,819	13,0518.8	1.38E+08	4.06E+08	28,858.85	45,666.3	23,065,689
	worst	1343.015	2.86E+10	28,0915.2	5.28E+10	18,693.2	1.34E+10	91,852,073	32,1861.4	7.65E+08	6.82E+08	53,232,163	1.03E+09	47,290,081
	std	4.660414	7.89E+09	11,6012.5	1.56E+10	5419.363	4.06E+09	14,937,750	88,542.93	3.35E+08	1.35E+08	27,629,074	5.45E+08	11,773,479
	median	1341.801	2.16E+10	10,1725.4	3.78E+10	18,212.2	8.22E+09	85,445,485	188,908.3	1.57E+08	4.54E+08	4,956,805	3E+08	35,589,250
	rank	1	12	3	13	2	11	7	4	8	10	5	9	6
C17-F14	mean	1429.458	22,138,175	1,043,123	41,274,749	1558.988	2,291,762	4,066,361	162,996.9	982,315.2	738,324.2	12,918,985	489,682	9,561,064
	best	1425.995	7,231,304	323,256	12,659,192	1546.115	605,547.5	3,600,215	103,335.7	76,691.77	608,829.9	2,929,237	176,046.7	4,704,918
	worst	1431.939	43,338,397	2,484,230	83,568,278	1582.843	3,634,801	4,832,202	316,175.9	1,895,369	851,820.3	21,211,653	784,154.5	16,455,299
	std	2.852761	16,583,070	1,069,026	32,825,935	18.30144	1,366,949	578,994.2	111,516.1	808,058.7	137,939	9,020,287	270,853.1	5,399,179
	median	1429.95	18,991,500	682,503.8	34,435,763	1553.497	2,463,349	3,916,514	116,238.1	978,600	746,323.4	13,767,524	499,263.5	8,542,020
	rank	1	12	7	13	2	8	9	3	6	5	11	4	10
C17-F15	mean	1530.66	2.22E+09	32,718.17	3.57E+09	2239.736	1.45E+09	8,462,655	103,834.4	5,074,514	60,186,667	1.68E+08	9569.48	7,314,943
	best	1526.359	1.57E+09	20,328.87	2.79E+09	2110.365	5E+08	780,284.2	43,186.67	36,399.24	35,292,025	16,631.35	2695.893	2,485,932
	worst	1532.953	2.91E+09	59,974.55	4.23E+09	2382.754	3.16E+09	15,801,114	154,709.4	13,365,072	78,344,085	6.53E+08	18,491.01	15,875,307
	std	3.193106	6.85E+08	20,003.62	6.95E+08	156.9426	1.35E+09	7,185,508	53,956.69	6,329,484	19,594,013	3.52E+08	7647.803	6,444,348
	median	1531.664	2.2E+09	25,284.63	3.63E+09	2232.913	1.08E+09	8,634,612	108,720.8	3,448,292	63,555,279	10,185,651	8545.507	5,449,267
	rank	1	12	4	13	2	11	8	5	6	9	10	3	7
C17-F16	mean	2062.891	5745.424	4099.726	6855.564	2733.524	4345.037	5070.653	3223.862	3221.386	4261.553	3759.801	3235.29	3724.023
	best	1728.6	5021.146	3797.152	5232.864	2579.866	3842.738	4253.427	3028.869	2863.636	3913.829	3472.365	2862.784	3178.337
	worst	2242.663	7271.321	4460.874	10,097.6	2996.441	4587.664	5643.426	3415.328	3726.251	4499.411	4087.984	3654.38	4158.479
	std	253.4793	1148.025	329.1293	2425.116	213.3552	379.1183	662.8411	172.0789	440.5687	269.1825	338.718	424.9433	463.8989
	median	2140.15	5344.614	4070.44	6045.895	2678.896	4474.873	5192.879	3225.626	3147.828	4316.486	3739.427	3211.998	3779.638
	rank	1	12	8	13	2	10	11	4	3	9	7	5	6
C17-F17	mean	2021.151	6861.854	3396.275	9790.292	2543.397	3736.742	4228.425	2980.016	2892.828	3900.141	3619.725	3219.341	3418.688
	best	1900.43	5295.665	3005.885	7230.627	2472.901	3056.519	3816.979	2498.974	2763.483	3344.926	3228.971	3028.007	3217.262
	worst	2138.267	8331.744	3851.936	12,612.77	2598.685	4136.552	4427.228	3401.68	3134.984	4230.555	3885.98	3511.96	3621.124
	std	146.0805	1361.803	434.8743	2412.363	58.14989	512.1874	310.0241	406.0587	180.4802	427.0499	308.8662	250.608	205.6284
	median	2022.954	6910.003	3363.64	9658.884	2551.001	3876.95	4334.746	3009.705	2836.423	4012.542	3681.974	3168.699	3418.184
	rank	1	12	6	13	2	9	11	4	3	10	8	5	7
C17-F18	mean	1830.62	64,614,732	2,061,097	95,851,415	25,555.65	29,921,140	38,563,414	2,256,925	4,888,694	7,002,910	7,180,913	706,632.7	8,087,329
	best	1822.239	51,707,525	270,404.9	43,098,156	3688.893	2,689,736	10,443,467	1,328,303	936,181	4,814,516	3,394,229	300,329.9	2,900,345
	worst	1841.673	76,192,919	3,774,095	1.33E+08	38,192.73	85,479,161	69,803,917	3,512,025	9,749,185	9,733,756	13,417,427	1,157,206	19,439,061
	std	8.863799	11,510,224	1,931,071	48,091,456	16,404.48	41,406,915	31,950,414	1,136,560	5,005,928	2,264,668	4,972,069	427,457.7	8,313,863
	median	1829.285	65,279,243	2,099,944	1.04E+08	30,170.48	15,757,831	37,003,137	2,093,686	4,434,706	6,731,684	5,955,997	684,497.7	5,004,954
	rank	1	12	4	13	2	10	11	5	6	7	8	3	9
C17-F19	mean	1925.185	2.32E+09	221,975.9	3.28E+09	2077.524	2.28E+09	5,839,914	4,374,448	992,916.8	43,270,616	386,089.3	336,264.1	846,771.8
	best	1924.437	1.11E+09	78,106.34	2.21E+09	2017.947	8,346,421	878,605.2	3,329,863	486,155.2	36,735,230	222,137.6	2767.461	662,438.1
	worst	1926.121	3.88E+09	457,483.6	4.05E+09	2106.959	6.67E+09	13,763,901	5,424,967	1,526,502	54,948,013	845,719.2	839,444.5	1,146,958
	std	0.861219	1.27E+09	179,180.7	8.92E+08	44.25295	3.24E+09	6,026,000	930,913.9	473,371.6	8,823,346	333,598.3	434,170.1	248,843.6
	median	1925.091	2.15E+09	176,156.8	3.42E+09	2092.596	1.23E+09	4,358,575	4,371,482	979,505	40,699,610	238,250.2	251,422.2	788,845.3
	rank	1	12	3	13	2	11	9	8	7	10	5	4	6
C17-F20	mean	2160.172	3643.744	3162.642	3872.417	2645.336	3307.283	3576.753	3174.558	2614.794	3598.47	3825.639	3182.392	3078.958
	best	2104.423	3321.011	2663.899	3597.129	2368.73	2883.838	3286.472	2964.002	2407.186	3523.56	3591.53	2866.252	2986.774
	worst	2323.891	3788.589	3609.976	4028.18	2920.336	3500.099	4068.862	3607.955	2803.756	3711.266	4098.01	3324.36	3179.235
	std	118.7931	238.5395	433.907	208.6822	253.4857	309.8258	374.6389	327.1354	227.4299	94.65403	226.473	231.1378	85.67285
	median	2106.186	3732.688	3188.346	3932.179	2646.14	3422.597	3475.84	3063.137	2624.118	3579.528	3806.507	3269.477	3074.912
	rank	1	11	5	13	3	8	9	6	2	10	12	7	4
C17-F21	mean	2314.895	2911.296	2708.199	2944.32	2445.682	2881.393	2873.592	2552.141	2507.304	2765.108	2782.422	2625.187	2703.313
	best	2309.045	2881.915	2601.485	2852.003	2426.012	2791.667	2773.116	2523.981	2458.18	2741.518	2723.144	2561.21	2680.796
	worst	2329.683	2939.209	2867.073	3021.404	2469.568	3024.514	2957.342	2586.126	2545.779	2806.139	2818.91	2714.661	2722.612
	std	10.75977	33.25097	123.952	86.19918	24.23715	109.0393	85.20166	35.23339	40.96419	32.60945	46.08534	72.90841	20.6077
	median	2310.426	2912.031	2682.12	2951.936	2443.575	2854.696	2881.956	2549.229	2512.629	2756.387	2793.816	2612.438	2704.922
	rank	1	12	7	13	2	11	10	4	3	8	9	5	6
C17-F22	mean	3095.169	13,541.5	10,253.37	14,628.25	5296.695	12,479.83	12,417.09	8414.581	8307.775	14,155.83	10,502.69	9065.003	8273.435
	best	2300	13,306.65	8551.137	14,094.07	2319.709	12,172.5	12,192.35	6411.778	7766.883	13,363.28	10,038.76	7990.454	3782.2
	worst	5480.678	13,892.82	12,094	15,153.48	8384.778	12,641.71	12,871.72	9834	8789.711	14,962.82	10,913.82	9810.043	12,642.36
	std	1730.769	289.6574	1783.239	479.8504	3573.342	238.4903	337.2136	1579.056	458.2178	922.9868	390.3125	845.8556	4946.644
	median	2300	13,483.25	10,184.17	14,632.73	5241.148	12,552.56	12,302.15	8706.272	8337.253	14,148.61	10,529.09	9229.756	8334.592
	rank	1	11	7	13	2	10	9	5	4	12	8	6	3
C17-F23	mean	2743.354	3689.579	3233.464	3755.06	2887.099	3623.224	3625.411	2972.584	2999.287	3224.747	4495.979	3307.134	3294.604
	best	2729.988	3621.225	3159.5	3712.118	2874.042	3441.415	3467.184	2936.881	2929.037	3150.466	4326.657	3249.029	3181.817
	worst	2752.657	3772.607	3306.203	3790.727	2906.781	3914.242	3711.376	3036.371	3118.56	3283.576	4642.809	3356.671	3417.095
	std	10.90099	71.67639	74.93054	36.15864	15.31114	244.6359	119.6447	50.82305	89.74451	59.99883	141.3101	61.34768	104.9761
	median	2745.387	3682.242	3234.076	3758.696	2883.787	3568.618	3661.542	2958.542	2974.775	3232.472	4507.225	3311.417	3289.752
	rank	1	11	6	12	2	9	10	3	4	5	13	8	7
C17-F24	mean	2919.043	4054.247	3450.701	4292.706	3063.289	3876.423	3724.834	3123.737	3178.789	3394.555	4202.741	3407.358	3581.48
	best	2909.046	3834.477	3350.833	3871.663	3033.954	3797.469	3624.721	3095.062	3097.68	3323.599	4168.799	3264.631	3542.81
	worst	2924.412	4553.465	3616.246	5332.137	3100.834	3995.323	3772.58	3150.743	3292.593	3450.283	4250.117	3540.786	3671.707
	std	7.426653	365.0322	125.0123	761.4861	32.75587	98.97493	74.03394	27.38206	89.38906	62.56123	39.22231	133.3472	65.79617
	median	2921.358	3914.522	3417.862	3983.512	3059.185	3856.449	3751.017	3124.572	3162.442	3402.168	4196.024	3412.007	3555.702
	rank	1	11	7	13	2	10	9	3	4	5	12	6	8
C17-F25	mean	2983.145	7840.95	3161.682	10,719.34	3066.596	5602.111	4003.228	3055.294	3899.38	4192.377	4109.234	3112.703	3912.044
	best	2980.235	6532.25	3139.605	8691.316	3046.332	4634.032	3650.751	3027.752	3729.328	3770.211	3810.836	3074.445	3816.771
	worst	2991.831	8671.091	3199.227	11,966.67	3084.832	6525.356	4267.227	3072.492	4076.001	4706.468	4679.679	3157.627	4018.564
	std	6.301777	1031.155	28.22414	1674.205	17.3182	884.9832	285.4902	21.50476	197.6538	513.2887	445.8802	45.2687	90.35902
	median	2980.257	8080.23	3153.949	11109.69	3067.611	5624.529	4047.468	3060.466	3896.096	4146.414	3973.21	3109.37	3906.421
	rank	1	12	5	13	3	11	8	2	6	10	9	4	7
C17-F26	mean	3776.432	12,636.24	9947.181	13,487.2	3334.791	11,373.32	12,403.24	5464.064	6090.526	8864.466	10,440.88	7481.348	8229.115
	best	3748.807	12,413.2	9479.973	12,926.99	3135.514	9523.972	11,634.58	5015.351	5727.379	8146.697	10,144.46	7017.238	6617.009
	worst	3793.643	12,836.52	10,373.29	14,293.02	3620.179	12,504.6	13,871.88	5676.619	6416.408	9551.372	10,765.37	7950.947	10,360.36
	std	21.16788	200.8083	397.9168	640.6481	239.2718	1402.329	1086.993	333.8182	371.3207	636.637	278.0636	453.9675	1936.675
	median	3781.639	12,647.63	9967.729	13,364.39	3291.736	11,732.36	12,053.25	5582.144	6109.158	8879.897	10,426.85	7478.604	7969.545
	rank	2	12	8	13	1	10	11	3	4	7	9	5	6
C17-F27	mean	3251.26	4604.683	3785.005	4768.312	3381.636	4527.175	4311.872	3363.123	3603.31	3768.097	7448.439	3608.047	4298.472
	best	3227.701	4328.522	3742.878	4442.58	3274.957	3896.836	3813.752	3327.505	3563.181	3613.084	7240.195	3379.099	4201.797
	worst	3313.631	4793.514	3853.479	5001.772	3480.831	4957.193	4822.33	3411.334	3658.614	3915.971	7759.333	3820.856	4436.656
	std	45.39257	226.2235	57.51471	285.3473	91.87787	504.5393	511.8991	40.28773	49.36585	149.5813	273.5143	213.321	110.8682
	median	3231.854	4648.348	3771.831	4814.447	3385.378	4627.336	4305.702	3356.826	3595.723	3771.666	7397.113	3616.117	4277.718
	rank	1	11	7	12	3	10	9	2	4	6	13	5	8
C17-F28	mean	3258.849	7995.78	3559.18	10,110.21	3350.874	6721.897	4620.526	3293.53	4258.861	4989.789	4826.547	3800.23	4809.503
	best	3258.849	7261.377	3483.448	8998.327	3314.656	5525.71	4089.321	3277.285	4020.132	4448.421	4770.251	3521.168	4592.939
	worst	3258.849	9864.723	3639.697	13,054.31	3395.35	7951.765	4827.562	3306.233	4558.861	5468.299	4925.914	4249.367	4968.115
	std	0	1366.648	84.93991	2139.942	43.07498	1334.759	386.6102	14.34867	271.1234	455.7595	75.25557	342.051	196.7871
	median	3258.849	7428.51	3556.788	9194.103	3346.745	6705.057	4782.609	3295.302	4228.226	5021.218	4805.012	3715.193	4838.479
	rank	1	12	4	13	3	11	7	2	6	10	9	5	8
C17-F29	mean	3263.038	12,318.61	5299.609	17,388.78	4082.162	6507.888	8359.279	4724.924	4757.362	6192.417	7611.6	4727.779	5858.844
	best	3247.132	8329.778	5194.722	9469.678	3730.764	6141.641	5766.427	4358.921	4577.561	5367.093	6357.457	4485.418	5558.764
	worst	3278.787	16,671.54	5379.045	27,143.86	4323.353	6956.538	10,777.95	5273.739	5037.961	7042.906	9841.389	4832.262	6413.197
	std	18.99818	4176.796	84.21284	8564.455	291.6212	366.2191	2246.896	423.0455	231.1973	860.2741	1716.019	177.199	432.7455
	median	3263.116	12,136.57	5312.334	16,470.79	4137.266	6466.687	8446.367	4633.518	4706.962	6179.835	7123.777	4796.718	5731.708
	rank	1	12	6	13	2	9	11	3	5	8	10	4	7
C17-F30	mean	623,575.2	2.8E+09	18,892,529	4.69E+09	1,630,658	1.42E+09	1.36E+08	60,436,983	1.19E+08	2.57E+08	1.58E+08	4,325,487	50,145,531
	best	582,411.6	2.16E+09	11,594,191	2.88E+09	1,237,598	1.74E+08	91,745,090	54,760,529	57,828,333	1.79E+08	1.21E+08	3,159,284	40,452,446
	worst	655,637.4	3.8E+09	25,767,629	7.36E+09	2,647,612	2.87E+09	1.87E+08	69,458,205	1.77E+08	3.25E+08	2.07E+08	5,901,616	70,259,757
	std	35,550.35	7.78E+08	7,624,147	2.1E+09	741,170.3	1.51E+09	52,122,607	6,967,947	65,532,625	66,719,715	39,154,562	1,485,469	15,004,001
	median	628,125.9	2.62E+09	19,104,147	4.26E+09	1,318,710	1.31E+09	1.32E+08	58,764,598	1.21E+08	2.62E+08	1.52E+08	4,120,524	44,934,960
	rank	1	12	4	13	2	11	8	6	7	10	9	3	5
Sum rank		30	335	166	367	63	294	269	112	144	248	254	150	207
Mean rank		1.034483	11.55172	5.724138	12.65517	2.172414	10.13793	9.275862	3.862069	4.965517	8.551724	8.758621	5.172414	7.137931
Total rank		1	12	6	13	2	11	10	3	4	8	9	5	7

**Table 4:** Optimization results of CEC 2017 test suite (dimension = 100).

		GAO	WSO	AVOA	RSA	MPA	TSA	WOA	MVO	GWO	TLBO	GSA	PSO	GA
C17-F1	mean	100	1.42E+11	3.33E+09	1.99E+11	5.05E+08	1.08E+11	5.36E+10	1.18E+08	4.88E+10	7.79E+10	1.16E+11	1.72E+10	4.79E+10
	best	100	1.39E+11	1.64E+09	1.96E+11	3.82E+08	9.48E+10	5.06E+10	93,364,159	4.23E+10	7.41E+10	1.07E+11	1.16E+10	4.54E+10
	worst	100	1.46E+11	4.78E+09	2.01E+11	6.38E+08	1.2E+11	6E+10	1.43E+08	5.52E+10	8.58E+10	1.24E+11	2.33E+10	5.42E+10
	std	1.26E−14	3.17E+09	1.4E+09	2.5E+09	1.34E+08	1.15E+10	4.68E+09	22,474,625	6.67E+09	5.86E+09	8.07E+09	7.02E+09	4.56E+09
	median	100	1.42E+11	3.45E+09	2E+11	5E+08	1.08E+11	5.19E+10	1.17E+08	4.89E+10	7.58E+10	1.17E+11	1.69E+10	4.61E+10
	rank	1	12	4	13	3	10	8	2	7	9	11	5	6
C17-F3	mean	300	383,842.5	297,065.6	293,794.9	153,298.3	328,723.6	691,639.7	416,132.3	332,672.7	271,228.7	311,511.4	479,906.4	510,979.9
	best	300	347,055.6	291,126.5	287,981.8	117,347.5	262,753.2	603,296.5	350,527.3	302,866	254,325.9	293,641.2	363,707.2	486,401.5
	worst	300	404,518.9	307,223.7	297,843.6	185,492.3	374,459.6	801,936.8	493,241.5	361,288.5	281,529.8	337,941.2	667,849.7	530,908
	std	0	28,746.65	7991.764	4536.701	32,202.67	51,409.18	93,079.01	77,508.77	34,488.75	12,808.7	20,829.9	152,845.3	20,545.72
	median	300	391,897.7	294,956.2	294,677.1	155,176.7	338,840.9	680,662.7	410,380.2	333,268.2	274,529.5	307,231.6	444,034.3	513,305
	rank	1	9	5	4	2	7	13	10	8	3	6	11	12
C17-F4	mean	602.1722	38,110.01	1463.253	64,164.55	1006.509	13,783.02	9464.142	786.2998	3945.925	9294.052	29,213.19	2245.803	7984.771
	best	592.0676	35,091.62	1264.191	58,182.9	897.1236	9057.49	8090.444	730.426	3070.574	8855.585	23,271.15	1426.042	7539.968
	worst	612.2769	41,757.16	1585.01	71,464.69	1120.178	18,294.93	10,365.01	824.3422	5875.795	10,052.86	33,033.64	2784.574	8489.108
	std	12.6933	3120.87	163.7911	5990.231	117.2572	4153.933	1053.821	43.67809	1411.052	614.74	5126.89	636.9998	473.8265
	median	602.1722	37,795.63	1501.906	63,505.3	1004.366	13,889.83	9700.557	795.2156	3418.665	9133.882	30,273.98	2386.298	7955.004
	rank	1	12	4	13	3	10	9	2	6	8	11	5	7
C17-F5	mean	512.9345	1822.958	1254.804	1797.302	1180.149	1951.64	1694.074	1188.948	1144.611	1723.369	1273.26	1338.401	1479.648
	best	510.9445	1810.032	1247.779	1762.886	1058.321	1925.842	1597.583	1106.621	1106.334	1709.908	1229.786	1249.81	1365.867
	worst	514.9244	1842.261	1264.602	1836.005	1262.3	1971.671	1817.129	1243.386	1180.804	1733.231	1309.72	1468.787	1563.629
	std	1.976192	15.07849	7.699721	33.55469	107.0527	21.56576	100.3657	71.93899	33.26408	12.23907	38.09065	109.6983	94.87956
	median	512.9345	1819.769	1253.418	1795.158	1199.987	1954.523	1680.793	1202.892	1145.653	1725.168	1276.766	1317.504	1494.549
	rank	1	12	5	11	3	13	9	4	2	10	6	7	8
C17-F6	mean	600	692.577	655.5795	691.1357	635.2408	696.3684	690.4931	666.174	637.6212	671.6821	657.3416	655.2134	656.5741
	best	600	689.8974	651.7584	686.6009	631.618	685.6778	681.9086	660.1273	634.2029	665.0953	654.737	648.7785	651.1896
	worst	600	695.4305	659.2454	693.6932	641.3751	704.1858	704.4683	670.9605	642.4555	676.195	660.4212	659.6686	661.1833
	std	0	2.546137	3.456439	3.386495	5.031692	9.392332	10.89493	5.209416	4.091298	5.732751	2.561423	5.86286	5.595732
	median	600	692.49	655.6572	692.1244	633.9852	697.8051	687.7977	666.804	636.9131	672.7191	657.1041	656.2032	656.9618
	rank	1	12	5	11	2	13	10	8	3	9	7	4	6
C17-F7	mean	811.392	3249.903	2809.103	3346.473	1780.479	3104.965	3225.886	1917.393	1930.261	2822.842	2843.951	2306.959	2388.427
	best	810.0205	3177.749	2668.95	3271.134	1724.798	2964.814	3121.438	1791.09	1765.371	2702.685	2727.712	2093.38	2301.084
	worst	813.1726	3328.526	2917.615	3420.581	1857.776	3239.49	3366.132	2013.472	2047.707	2918.788	3034.433	2407.096	2565.297
	std	1.589565	67.33718	133.3426	68.67265	62.52161	135.5128	122.8352	100.7463	129.6834	97.17736	144.9155	160.257	131.1636
	median	811.1874	3246.669	2824.924	3347.089	1769.67	3107.778	3207.986	1932.506	1953.982	2834.946	2806.83	2363.681	2343.663
	rank	1	12	7	13	2	10	11	3	4	8	9	5	6
C17-F8	mean	812.437	2212.881	1647.373	2258.275	1393.836	2194.03	2127.672	1413.475	1464.418	2073.614	1720.513	1621.273	1890.868
	best	808.9546	2175.495	1607.877	2244.776	1231.543	2149.448	1940.752	1286.824	1383.515	2020.033	1632.323	1580.032	1828.262
	worst	816.9143	2263.473	1682.993	2272.856	1494.677	2267.22	2267.929	1577.554	1581.495	2124.601	1829.583	1712.871	1934.221
	std	3.697116	47.32375	36.88267	13.67073	125.6233	59.80833	175.3943	131.2531	104.9716	46.47028	92.56546	66.9778	50.5737
	median	811.9395	2206.278	1649.31	2257.735	1424.562	2179.726	2151.004	1394.762	1446.331	2074.911	1710.072	1596.095	1900.494
	rank	1	12	6	13	2	11	10	3	4	9	7	5	8
C17-F9	mean	900	76,816.82	24,403.01	66,222.08	21,063.39	101,805.1	65,821.32	51,366.63	32,197.6	63,893.94	21,998.26	29,657.46	40,458.63
	best	900	68,900.42	20,792.7	63,921.65	19,605.36	83,970.72	51,834.98	43,765.53	20,725.61	61,124.6	20,701.12	25,525.09	36,964.91
	worst	900	88,390.72	27,225.2	68,008.19	21,723.38	126,398.6	82,335.26	58,117.91	42,909.41	65,321.41	23,120.05	32,788.17	45,299.31
	std	1.01E−13	9140.389	2905.49	1940.678	1065.91	19,383.02	16,524.1	6435.124	11,756.31	2089.674	1106.024	3542.998	3839.177
	median	900	74,988.06	24,797.07	66,479.23	21,462.41	98,425.45	64,557.51	51,791.54	32,577.68	64,564.88	22,085.94	30,158.28	39,785.15
	rank	1	12	4	11	2	13	10	8	6	9	3	5	7
C17-F10	mean	11,023.04	27,349.26	15,441.01	28,451.98	13,705.22	26,592.86	25,727.09	16,289.98	14,793.94	28,460.01	16,484.36	16,357.73	23,872.49
	best	9625.608	27,025.57	13,346.64	27,628.2	13,035.67	26,097.57	25,018.54	15,674.02	13,811.72	27,371.81	14,979.87	14,952.56	23,395.55
	worst	11,858.81	27,711.42	17,262.38	28,960.59	14,519.33	27,297.11	27,009.25	16,804.07	15,206.42	29,371.22	17,274.56	17,391.97	24,443.19
	std	1054.018	322.3919	1881.722	649.5138	681.7138	609.0236	998.3499	535.963	720.7513	899.0758	1173.583	1109.584	473.1087
	median	11,303.87	27,330.04	15,577.5	28,609.56	13,632.93	26,488.39	25,440.28	16,340.91	15,078.81	28,548.5	16,841.5	16,543.19	23,825.61
	rank	1	11	4	12	2	10	9	5	3	13	7	6	8
C17-F11	mean	1162.329	138,171.3	54,218.15	173,245.6	4617.217	55,259.59	174,853	4448.069	73,503.38	60,611.45	145,056.9	44,126.05	117,068.8
	best	1139.568	107,408.8	48,816.74	132,721.4	3646.106	25,475.78	101,876.7	3991.368	61,195.93	51,182.33	121,014.7	20,433.81	89,442.4
	worst	1220.662	160,596.2	64,555.37	246,485.9	5510.858	78,913.32	281,535.5	4786.813	82,633.13	77,155.18	169,243.8	89,549.74	161,296.6
	std	42.46,663	24,880.2	7905.572	55,743.48	873.2478	24,092.58	90,902.06	361.1768	10,009.55	12,382.24	21,675.86	33,640.04	34,264.38
	median	1144.542	142,340.1	51,750.24	156,887.5	4655.952	58,324.63	157,999.9	4507.047	75,092.23	57,054.15	144,984.6	33,260.32	108,768.1
	rank	1	10	5	12	3	6	13	2	8	7	11	4	9
C17-F12	mean	5974.805	8.83E+10	5.81E+08	1.44E+11	2.48E+08	4.76E+10	1.11E+10	3.08E+08	9.6E+09	1.84E+10	5.59E+10	8.47E+09	1.04E+10
	best	5383.905	6.27E+10	3.09E+08	1.07E+11	1.39E+08	2.44E+10	8.99E+09	2.11E+08	6.66E+09	1.44E+10	4.85E+10	1.13E+09	9.44E+09
	worst	6570.199	9.84E+10	9.16E+08	1.67E+11	2.98E+08	7.89E+10	1.26E+10	4.73E+08	1.14E+10	2.53E+10	6.58E+10	1.61E+10	1.22E+10
	std	537.9317	1.86E+10	2.83E+08	2.96E+10	80,251,697	2.48E+10	1.67E+09	1.29E+08	2.23E+09	5.4E+09	7.83E+09	7.41E+09	1.38E+09
	median	5972.559	9.6E+10	5.49E+08	1.5E+11	2.79E+08	4.35E+10	1.13E+10	2.75E+08	1.02E+10	1.69E+10	5.47E+10	8.34E+09	9.87E+09
	rank	1	12	4	13	2	10	8	3	6	9	11	5	7
C17-F13	mean	1407.28	2.33E+10	93,518.05	3.57E+10	92,388.84	1.79E+10	4.38E+08	307,656.4	7.93E+08	2.36E+09	7.31E+09	1.48E+09	1.46E+08
	best	1371.145	2.03E+10	62,963.2	2.76E+10	39,601.01	1.27E+10	3.11E+08	268,601.7	68,370,498	1.63E+09	4.49E+09	1.63E+08	1.15E+08
	worst	1439.935	2.58E+10	119,547	4.05E+10	229,396.8	2.14E+10	5.92E+08	373,502.2	2.1E+09	2.85E+09	9.38E+09	2.67E+09	1.76E+08
	std	37.80433	3.15E+09	25,701.38	6.46E+09	99,954.78	4.01E+09	1.57E+08	50,861.93	1.02E+09	6.06E+08	2.23E+09	1.34E+09	34,572,729
	median	1409.02	2.35E+10	95,780.99	3.74E+10	50,278.77	1.87E+10	4.24E+08	294,260.8	5.03E+08	2.47E+09	7.68E+09	1.54E+09	1.47E+08
	rank	1	12	3	13	2	11	6	4	7	9	10	8	5
C17-F14	mean	1467.509	38,082,540	5,605,598	66,800,415	86,803.32	7,468,562	12,208,820	2,554,157	8,074,153	11,671,525	9,650,213	693,949.6	8,816,297
	best	1458.803	32,883,853	3,415,353	60,921,097	24,818.95	3,392,874	7,027,213	779,198.2	5,111,901	8,699,694	7,452,511	330,058.5	4,938,542
	worst	1472.733	43,502,874	9,299,515	73,125,403	184,373.4	14,562,847	16,677,476	3,504,230	12,091,525	14,923,989	14,459,922	1,441,316	12,994,340
	std	6.576739	5,070,725	2,823,155	6,372,141	77,728.84	5,353,785	4,317,050	1,323,088	3,325,705	3,539,240	3,527,329	550,201.6	3,648,073
	median	1469.25	37,971,717	4,853,762	66,577,581	69,010.46	5,959,264	12,565,295	2,966,600	7,546,593	11,531,208	8,344,210	502,212.2	8,666,153
	rank	1	12	5	13	2	6	11	4	7	10	9	3	8
C17-F15	mean	1609.893	1.29E+10	77,286.65	1.97E+10	53,518.19	1.01E+10	58,778,711	112,631.2	4.2E+08	9.99E+08	1.04E+09	2.8E+08	10,645,222
	best	1551.154	1.19E+10	66,065.03	1.41E+10	15,481.08	2.1E+08	32,741,200	74,503.83	27,578,628	3.34E+08	4.17E+08	59,310.67	6,867,523
	worst	1652.294	1.45E+10	90,717.93	2.46E+10	81,241.61	1.89E+10	1.13E+08	162,708.2	1.26E+09	2.13E+09	1.33E+09	1.1E+09	18,137,162
	std	48.04352	1.22E+09	13,326.2	5.67E+09	30,201.72	8.84E+09	39,831,191	41,080.69	6.2E+08	8.58E+08	4.6E+08	5.98E+08	5,569,986
	median	1618.063	1.26E+10	76,181.82	2.01E+10	58,675.04	1.06E+10	44,721,842	106,656.4	1.97E+08	7.65E+08	1.21E+09	7,243,656	8,788,101
	rank	1	12	3	13	2	11	6	4	8	9	10	7	5
C17-F16	mean	2711.795	16,653.33	6752.877	19,761.53	5402.929	12,984.33	14,386.34	6302.297	5887.338	10,410.19	10,042.59	6208.393	9611.617
	best	2171.69	15,547.12	5757.308	15,679.72	5307.987	10,829.43	11,844.45	5656.217	5400.708	9952.753	8810.787	5969.755	8736.575
	worst	3397.326	17,141.74	7390.032	22,016.02	5537.679	15,421.12	15,871.81	6741.128	6461.293	11,325.02	11,504.13	6395.272	10,309.06
	std	554.7769	808.8727	770.3875	3124.823	107.4954	2051.445	1956.809	519.8196	601.7643	695.8643	1314.398	191.9232	776.5046
	median	2639.081	16,962.24	6932.084	20,675.18	5383.024	12,843.39	14,914.56	6405.921	5843.676	10,181.5	9927.721	6234.271	9700.415
	rank	1	12	6	13	2	10	11	5	3	9	8	4	7
C17-F17	mean	2716.564	3,542,232	5563.724	6,967,945	4559.569	184,224.2	14,958.36	4828.586	5278.937	7997.77	39,614.15	5776.221	6664.501
	best	2275.021	1,038,583	5357.851	1,889,136	4342.933	9201.727	9460.883	4476.871	4357.063	7878.394	26,201.78	5529.464	6522.225
	worst	3429.127	8,058,357	5938.059	16,032,637	4772.105	488,362.9	24,891.01	5113.016	6672.656	8154.271	64,020.8	5959.869	6842.104
	std	559.6669	3,599,997	285.066	7,238,974	230.5594	227,763.7	7565.855	354.7562	1118.696	137.2394	18,218.35	204.2189	146.0004
	median	2581.054	2,535,993	5479.492	4,975,004	4561.62	119,666.1	12,740.77	4862.229	5043.015	7979.208	34,117.01	5807.776	6646.838
	rank	1	12	5	13	2	11	9	3	4	8	10	6	7
C17-F18	mean	1903.746	49,047,583	2,391,305	86,532,758	221,894	12,535,993	10,102,031	4,147,266	9,226,887	13,629,241	9,895,380	5,430,289	5,095,315
	best	1881.15	22,229,584	1,194,868	33,597,693	154,720.5	4,704,257	7,515,295	3,100,094	2,945,821	10,042,328	4,568,763	3,356,167	4,082,969
	worst	1919.921	88,697,678	3,786,728	1.58E+08	399,841.5	25,610,542	11,954,144	6,942,446	14,885,570	19,248,180	21,987,153	7,805,783	7,356,158
	std	21.08244	30,890,671	1,275,149	57,152,959	129,457.6	10,248,095	2,197,656	2,034,492	5,343,871	4,298,217	8,932,913	2,242,128	1,673,052
	median	1906.955	42,631,536	2,291,812	77,153,607	166,507	9,914,588	10,469,342	3,273,261	9,538,078	12,613,229	6,512,802	5,279,603	4,471,066
	rank	1	12	3	13	2	10	9	4	7	11	8	6	5
C17-F19	mean	1972.839	1.07E+10	2,450,387	1.88E+10	267,860	4.24E+09	1.13E+08	14,008,315	3.03E+08	5.62E+08	1.33E+09	2.26E+08	10,782,637
	best	1967.139	9.42E+09	980,922.2	1.37E+10	56,413.89	1.88E+09	44,711,356	8,206,646	2,441,625	2.44E+08	2.39E+08	37,668,367	5,494,722
	worst	1977.869	1.26E+10	4,488,740	2.34E+10	453,682.7	8.42E+09	1.9E+08	22,221,007	9.11E+08	1.29E+09	2.5E+09	4.9E+08	19,497,003
	std	4.935585	1.55E+09	1,615,719	4.34E+09	179,384.6	3.15E+09	73,083,332	7,532,410	4.61E+08	5.36E+08	1.23E+09	2.38E+08	6,754,581
	median	1973.174	1.04E+10	2,165,944	1.9E+10	280,671.7	3.33E+09	1.08E+08	12,802,803	1.49E+08	3.56E+08	1.28E+09	1.89E+08	9,069,411
	rank	1	12	3	13	2	11	6	5	8	9	10	7	4
C17-F20	mean	3192.04	6799.892	5863.746	7013.392	4445.793	6582.15	6592.755	5556.399	5777.929	6765.252	5985.866	5185.77	5944.842
	best	2806.762	6627.06	5556.257	6904.666	4389.302	6043.63	6217.504	5281.353	4719.943	6071.329	5603.856	4568.552	5417.463
	worst	3662.121	6958.325	6114.165	7084.407	4506.979	7242.068	6913.179	6021.008	6553.599	7070.192	6214.763	5924.889	6361.49
	std	477.9749	150.8324	284.3249	83.28669	55.23384	559.0573	333.4967	349.4198	984.7558	507.6215	295.3101	631.4043	486.8695
	median	3149.639	6807.092	5892.281	7032.248	4443.445	6521.452	6620.168	5461.617	5919.088	6959.744	6062.424	5124.82	6000.208
	rank	1	12	6	13	2	9	10	4	5	11	8	3	7
C17-F21	mean	2342.155	4030.891	3510.961	4134.871	2811.333	3892.34	3978.765	3150.553	2931.257	3545.469	4389.644	3439.937	3302.062
	best	2338.689	3985.966	3336.056	4069.352	2768.268	3771.866	3719.434	3096.12	2863.353	3408.401	3921.956	3284.861	3273.431
	worst	2346.015	4094.767	3627.267	4177.762	2844.976	3978.86	4179.226	3262.762	2976.534	3695.242	4761.858	3740.055	3339.036
	std	3.664912	54.72408	136.7255	52.09297	35.38474	110.7836	222.9256	83.3817	52.53036	132.5194	381.2425	225.2292	30.25229
	median	2341.959	4021.415	3540.261	4146.186	2816.044	3909.317	4008.2	3121.666	2942.57	3539.117	4437.381	3367.415	3297.891
	rank	1	11	7	12	2	9	10	4	3	8	13	6	5
C17-F22	mean	11,739	29,370.52	19,711.12	30,768.17	18,401.05	28,523.67	27,146.66	17,214.54	22,298.55	30,664.87	20,471.56	21,114.04	26,861.63
	best	11,119.08	28,683.13	18,482.81	30,313.72	17,099.57	27,540.69	25,876.73	16,180.59	18,097.38	29,834.73	19,786.44	19,847.43	26,175.5
	worst	12,601.6	29,715.62	21,309.93	31,235.73	20,018.39	29,310.11	28,022.86	17,998.21	31,748.26	31,044.72	20,799.05	22,659.88	27,485.86
	std	710.0872	506.5825	1319.333	474.8296	1339.001	797.8782	1024.07	902.6408	6997.36	620.0927	504.5512	1302.337	667.1921
	median	11,617.67	29,541.65	19,525.86	30,761.61	18,243.11	28,621.94	27,343.52	17,339.67	19,674.28	30,890.02	20,650.37	20,974.44	26,892.59
	rank	1	11	4	13	3	10	9	2	7	12	5	6	8
C17-F23	mean	2877.697	5006.271	3970.619	5008.105	3281.804	5108.575	4848.228	3440.536	3553.965	4056.188	7171.538	4611.657	4100.543
	best	2872.107	4790.632	3900.775	4778.495	3266.443	4459.054	4729.584	3361.269	3525.424	4009.727	6660.73	4165.789	4042.702
	worst	2884.013	5261.75	4047.115	5186.927	3312.206	5991.468	4970.577	3545.696	3592.285	4122.332	7535.123	4845.715	4155.234
	std	5.674312	228.9553	74.83691	184.2031	22.41464	745.7664	125.832	85.12362	32.16584	52.00293	429.2467	334.1677	64.93272
	median	2877.334	4986.35	3967.293	5033.499	3274.283	4991.889	4846.375	3427.59	3549.076	4046.346	7245.149	4717.562	4102.117
	rank	1	10	5	11	2	12	9	3	4	6	13	8	7
C17-F24	mean	3327.407	7801.681	5114.695	9482.038	3703.964	6220.646	5972.339	3919.074	4196.735	4586.681	9749.777	5615.49	5117.215
	best	3295.518	6191.278	4921.353	6514.465	3656.964	5795.549	5613.836	3854.929	3986.73	4394.007	9185.954	5302.044	5033.637
	worst	3357.991	8900.02	5273.69	11,456.62	3768.186	6501.711	6529.455	4016.905	4376.127	4779.769	11,221.45	6027.595	5258.358
	std	32.22323	1409.723	167.7738	2604.444	57.64469	327.8437	435.2487	79.57128	220.1476	172.536	1068.987	354.1205	108.0711
	median	3328.059	8057.712	5131.868	9978.536	3695.354	6292.662	5873.032	3902.232	4212.042	4586.474	9295.854	5566.161	5088.433
	rank	1	11	6	12	2	10	9	3	4	5	13	8	7
C17-F25	mean	3185.232	13,529.95	4057.362	18,688.73	3670.92	9451.007	6751.414	3434.623	6009.092	8117.186	9926.001	4058.267	7249.185
	best	3137.371	12,891.38	3736.27	17,378.7	3498.856	8870.306	6222.759	3357.947	5886.475	7042.655	9174.159	3815.299	6642.467
	worst	3261.571	15,038.01	4340.986	21,648.26	3792.464	9824.884	7059.572	3496.658	6333.98	9539.192	11,233.8	4427.694	7851.922
	std	65.17161	1103.36	271.7265	2191	134.2336	469.9241	415.3623	62.94,321	236.5814	1237.829	986.7667	318.7455	685.3193
	median	3170.992	13,095.21	4076.096	17,863.99	3696.18	9554.419	6861.663	3441.944	5907.958	7943.448	9648.021	3995.038	7251.174
	rank	1	12	4	13	3	10	7	2	6	9	11	5	8
C17-F26	mean	5757.621	35,167.72	22,517.65	40,224.61	11,453.1	29,897.3	30,431.15	11,630.83	15,907.27	21,863.98	30,347.8	19,176.1	21,128.3
	best	5645.905	34,635.44	20,138.23	37,982.62	10,749.82	28,911.34	27,353.64	10,406.38	14,350.84	18,216.51	29,245.56	17,272.12	19,652.66
	worst	5844.642	35,653.47	24,926.9	41,621.02	12,187.39	30,511.54	33,052.29	13,730.92	17,185.39	26,505.71	31,963.87	20,940.26	22,125.46
	std	91.29453	453.0954	2255.433	1857.913	771.9566	759.8127	3004.529	1575.844	1323.715	3737.738	1264.799	1698.289	1169.621
	median	5769.969	35,190.99	22,502.73	40,647.39	11,437.59	30,083.17	30,659.34	11,193	16,046.42	21,366.86	30,090.88	19,246.01	21,367.54
	rank	1	12	8	13	2	9	11	3	4	7	10	5	6
C17-F27	mean	3309.493	8472.462	4065.701	10,998.58	3528.702	6150.218	5640.525	3605.339	3996.688	4207.603	12,507.41	3990.172	5196.33
	best	3278.01	7203.499	3921.095	8352.904	3490.261	5886.739	5039.378	3571.895	3854.157	3968.798	12,213.88	3815.176	4971.8
	worst	3344.5	9747.033	4311.362	13,743.24	3561.724	6472.084	6305.127	3689.085	4107.092	4605.08	12,737.12	4166.644	5535.875
	std	30.85754	1501.857	184.5849	3159.148	32.00686	274.7479	745.8619	61.13918	136.8499	308.7915	258.9872	207.5626	262.3963
	median	3307.732	8469.659	4015.173	10,949.09	3531.412	6121.026	5608.799	3580.188	4012.751	4128.266	12,539.32	3989.434	5138.822
	rank	1	11	6	12	2	10	9	3	5	7	13	4	8
C17-F28	mean	3322.242	18,389.38	4558.61	24,650.68	3759.304	13,947.47	9399.017	3492.414	8456.179	10,080.85	16,594.61	7067.786	10,348.93
	best	3318.742	17,167.17	4302.082	22,119.53	3638.049	11,064.52	8088.89	3423.227	7249.838	7994.173	14,371.5	4945.502	9470.138
	worst	3327.816	20,674.35	4735.228	27,803.32	3845.863	16,143.37	10,248.76	3564.13	10,195.47	11,924.7	18,271.02	10,653.51	11,327.38
	std	4.767125	1741.216	202.5309	2590.902	95.07424	2644.481	1002.041	62.67904	1354.176	1990.043	1774.385	2826.363	1074.312
	median	3321.205	17,858.01	4598.564	24,339.94	3776.653	14,290.99	9629.211	3491.149	8189.702	10,202.27	16,867.96	6336.069	10,299.1
	rank	1	12	4	13	3	10	7	2	6	8	11	5	9
C17-F29	mean	4450.696	157,317.8	9139.852	298,621.5	6805.141	16,662.4	15,041.71	8338.592	8016.546	11,529.03	22,196.27	8307.764	11,023.77
	best	4169.151	90,006.73	8041.684	160,652.4	6002.795	13,000.42	12,596.71	7548.871	7769.158	10,697.55	18,567.74	7738.827	10,779.1
	worst	4829.521	214,340.3	9695.661	414,215.6	7560.201	20,865.25	17,104.72	8887.513	8277.739	12,142.5	28,742.04	8977.924	11,517.96
	std	307.1569	57,574.73	814.4624	117,534.7	693.9889	3584.829	2382.752	617.3333	242.3973	662.0945	5174.887	661.4283	366.7167
	median	4402.056	162,462.1	9411.032	309,809	6828.783	16,391.97	15,232.7	8458.992	8009.643	11,638.04	20,737.64	8257.153	10,899.02
	rank	1	12	6	13	2	10	9	5	3	8	11	4	7
C17-F30	mean	5407.166	1.97E+10	24,130,069	3.21E+10	4,546,185	1.14E+10	1.28E+09	88,083,440	1.56E+09	3.22E+09	6.25E+09	5.15E+08	5.67E+08
	best	5337.48	1.73E+10	13,795,207	3E+10	2,025,882	6.93E+09	1.05E+09	54,762,921	6.42E+08	1.21E+09	4.46E+09	1.26E+08	4.73E+08
	worst	5557.155	2.14E+10	41,706,015	3.47E+10	7,423,374	1.41E+10	1.73E+09	1.08E+08	2.04E+09	5.97E+09	7.57E+09	1.59E+09	6.07E+08
	std	110.0477	1.89E+09	13,513,044	2.21E+09	2,713,659	3.42E+09	3.35E+08	25,767,589	6.85E+08	2.6E+09	1.43E+09	7.84E+08	68,320,415
	median	5367.014	2.01E+10	20,509,527	3.18E+10	4,367,742	1.23E+10	1.17E+09	94,878,086	1.78E+09	2.85E+09	6.49E+09	1.7E+08	5.93E+08
	rank	1	12	3	13	2	11	7	4	8	9	10	5	6
Sum rank		29	336	140	355	65	293	265	114	156	249	272	162	203
Mean rank		1	11.58621	4.827586	12.24138	2.241379	10.10345	9.137931	3.931034	5.37931	8.586207	9.37931	5.586207	7
Total rank		1	12	4	13	2	11	9	3	5	8	10	6	7

**Table 5:** Wilcoxon rank sum test results.

Compared Algorithm	Objective Function Type			
	CEC 2017			
	D = 10	D = 30	D = 50	D = 100
GAO vs. WSO	2.58E−34	2.58E−34	2.58E−34	2.58E−34
GAO vs. AVOA	3.03E−25	4.23E−21	2.58E−34	2.58E−34
GAO vs. RSA	2.58E−34	2.58E−34	2.58E−34	2.58E−34
GAO vs. MPA	1.61E−29	5.26E−16	8.68E−31	2.58E−34
GAO vs. TSA	7.63E−34	2.58E−34	2.58E−34	2.58E−34
GAO vs. WOA	7.63E−34	2.58E−34	2.58E−34	2.58E−34
GAO vs. MVO	6.08E−26	7.11E−21	2.58E−34	2.58E−34
GAO vs. GWO	8.19E−32	2.58E−34	2.58E−34	2.58E−34
GAO vs. TLBO	2.97E−32	2.58E−34	2.58E−34	2.58E−34
GAO vs. GSA	1.29E−27	6.21E−21	2.58E−34	2.58E−34
GAO vs. PSO	6.24E−28	1.89E−21	2.58E−34	2.58E−34
GAO vs. GA	5.17E−28	2.58E−34	2.58E−34	2.58E−34

**Table 6:** Optimization results of the CEC 2011 test suite.

		GAO	WSO	AVOA	RSA	MPA	TSA	WOA	MVO	GWO	TLBO	GSA	PSO	GA
C11-F1	mean	5.920103	16.74641	12.47935	20.60699	7.613273	17.40635	12.74259	13.42229	10.58714	17.43517	20.35526	16.99013	21.88339
	best	2E−10	13.86501	8.478707	18.18457	0.380987	16.50584	7.451926	11.38898	1.050147	16.64263	17.71226	10.14258	20.69375
	worst	12.30606	19.6453	16.37007	23.21547	12.69538	19.05236	16.77422	15.48484	16.38551	18.69533	22.1194	23.16349	24.33594
	std	7.476538	3.058894	4.906722	2.611096	6.161367	1.264455	4.734219	2.406333	7.278318	1.063321	2.051999	6.277694	1.854985
	median	5.687176	16.73766	12.53432	20.51396	8.688362	17.03359	13.37211	13.40768	12.45645	17.20137	20.79468	17.32722	21.25194
	rank	1	7	4	12	2	9	5	6	3	10	11	8	13
C11-F2	mean	−26.3179	−15.5172	−21.4594	−13.0022	−25.0711	−12.7516	−19.2977	−10.5326	−22.8812	−12.4007	−16.5475	−22.9249	−14.2156
	best	−27.0676	−16.7338	−22.0088	−13.416	−25.7089	−16.1048	−22.3643	−12.4163	−24.773	−13.5327	−21.127	−24.2021	−16.3132
	worst	−25.4328	−14.5198	−20.8937	−12.6713	−23.7335	−10.8719	−15.8013	−9.00571	−19.5197	−11.4832	−12.7537	−20.8714	−12.7783
	std	0.767703	1.191398	0.561161	0.413033	1.005704	2.689933	3.636791	1.601866	2.551853	0.95683	4.12006	1.566969	1.883805
	median	−26.3856	−15.4077	−21.4675	−12.9608	−25.421	−12.0149	−19.5127	−10.3543	−23.616	−12.2934	−16.1546	−23.313	−13.8855
	rank	1	8	5	10	2	11	6	13	4	12	7	3	9
C11-F4	mean	1.15E−05	1.15E−05	1.15E−05	1.15E−05	1.15E−05	1.15E−05	1.15E−05	1.15E−05	1.15E−05	1.15E−05	1.15E−05	1.15E−05	1.15E−05
	best	1.15E−05	1.15E−05	1.15E−05	1.15E−05	1.15E−05	1.15E−05	1.15E−05	1.15E−05	1.15E−05	1.15E−05	1.15E−05	1.15E−05	1.15E−05
	worst	1.15E−05	1.15E−05	1.15E−05	1.15E−05	1.15E−05	1.15E−05	1.15E−05	1.15E−05	1.15E−05	1.15E−05	1.15E−05	1.15E−05	1.15E−05
	std	2.08E−19	2.03E−11	2.33E−09	4.57E−11	1.3E−15	2.17E−14	1.58E−16	9.12E−13	3.57E−15	7.19E−14	1.58E−16	1.58E−16	1.58E−16
	median	1.15E−05	1.15E−05	1.15E−05	1.15E−05	1.15E−05	1.15E−05	1.15E−05	1.15E−05	1.15E−05	1.15E−05	1.15E−05	1.15E−05	1.15E−05
	rank	1	11	13	12	6	8	4	10	7	9	3	2	5
C11-F4	mean	0	0	0	0	0	0	0	0	0	0	0	0	0
	best	0	0	0	0	0	0	0	0	0	0	0	0	0
	worst	0	0	0	0	0	0	0	0	0	0	0	0	0
	std	0	0	0	0	0	0	0	0	0	0	0	0	0
	median	0	0	0	0	0	0	0	0	0	0	0	0	0
	rank	1	1	1	1	1	1	1	1	1	1	1	1	1
C11-F5	mean	−34.1274	−25.7765	−28.7022	−21.4775	−33.271	−27.837	−28.2791	−27.7131	−31.7667	−13.3239	−28.0292	−11.4033	−12.1662
	best	−34.7494	−26.8331	−29.7227	−23.4213	−33.8557	−31.7971	−28.4609	−31.7451	−34.1148	−15.2637	−31.5688	−14.3978	−13.2792
	worst	−33.3862	−24.7915	−28.1294	−19.226	−31.9367	−23.1816	−27.9722	−25.5891	−28.0378	−11.9458	−25.2806	−9.92524	−10.7534
	std	0.612958	0.950695	0.77194	2.38908	0.977388	3.870035	0.250642	3.142491	2.849728	1.541204	3.011529	2.295861	1.2422
	median	−34.1871	−25.7407	−28.4783	−21.6314	−33.6458	−28.1847	−28.3416	−26.7591	−32.4571	−13.043	−27.6336	−10.6451	−12.3162
	rank	1	9	4	10	2	7	5	8	3	11	6	13	12
C11-F6	mean	−24.1119	−15.0062	−19.4362	−14.1244	−22.6084	−9.25983	−20.2537	−11.008	−19.9677	−4.61236	−21.9655	−5.38056	−6.18492
	best	−27.4298	−15.3693	−21.0438	−14.6552	−25.7439	−17.0765	−22.8753	−17.9544	−22.7828	−5.25001	−25.9902	−8.32281	−10.6604
	worst	−23.0059	−14.6561	−17.7919	−13.074	−21.3201	−6.21322	−13.9021	−4.37018	−18.359	−4.37018	−18.7012	−4.37018	−4.37018
	std	2.415463	0.362766	1.575989	0.780164	2.313354	5.723676	4.671589	7.853513	2.296056	0.466472	3.484428	2.142492	3.286726
	median	−23.0059	−14.9997	−19.4545	−14.3841	−21.6849	−6.87477	−22.1187	−10.8537	−19.3646	−4.41462	−21.5854	−4.41462	−4.85453
	rank	1	7	6	8	2	10	4	9	5	13	3	12	11
C11-F7	mean	0.860699	1.525865	1.242141	1.802371	0.929865	1.257862	1.647066	0.887071	1.051349	1.625096	1.061967	1.100668	1.644276
	best	0.582266	1.451887	1.112942	1.598764	0.757869	1.085156	1.552518	0.840735	0.831043	1.466068	0.86985	0.853582	1.279595
	worst	1.025027	1.625584	1.376489	1.968587	1.011582	1.588703	1.802521	0.960573	1.258512	1.753067	1.246812	1.322056	1.83421
	std	0.219737	0.080974	0.155593	0.166559	0.127917	0.245827	0.117904	0.061752	0.190899	0.139933	0.185054	0.257758	0.274052
	median	0.91775	1.512994	1.239567	1.821066	0.975005	1.178795	1.616613	0.873488	1.057921	1.640626	1.065602	1.113517	1.731649
	rank	1	9	7	13	3	8	12	2	4	10	5	6	11
C11-F8	mean	220	277.756	238.4052	313.4897	222.463	253.2859	261.0367	223.905	226.789	223.905	243.6012	440.9517	222.5031
	best	220	254.6314	223.8045	277.5593	220	220	242.3511	220	220	220	220	245.4346	220
	worst	220	308.7868	253.606	352.6647	224.926	339.5656	302.0734	234.4201	233.5781	235.0201	285.4904	530.1433	229.4123
	std	0	25.96257	14.00974	33.66547	3.105763	63.2005	30.12558	7.661323	8.560688	8.097811	33.93451	146.9772	5.039474
	median	220	273.8029	238.1052	311.8675	222.463	226.789	249.8612	220.6	226.789	220.3	234.4572	494.1144	220.3
	rank	1	10	6	11	2	8	9	4	5	4	7	12	3
C11-F9	mean	8789.286	495,628.6	337,304.8	942,375.5	20,289.66	61,028.87	334,059.8	120,463.1	40,488.79	364,116.3	731,137.6	960,487.9	1,721,875
	best	5457.674	332,586	299,628.8	616,714.2	11,047.41	45,559.44	184,837.1	68,871.61	17,607.44	301,247	626,497.6	770,332.1	1,650,717
	worst	14,042.29	570,171.3	361,787	1,106,235	28,881.93	76,423.83	563,855.9	181,648.1	70,024.01	466,965.3	785,786.6	1,176,387	1,822,159
	std	4040.59	121,883.4	29,840.72	241,894.9	8616.991	14,638	188,476.8	50,889.21	24,188.06	79,408.16	77,704.14	237,012.5	91,820.42
	median	7828.591	539,878.5	343,901.7	1,023,277	20,614.66	61,066.11	293,773.2	115,666.3	37,161.85	344,126.5	756,133.1	947,616.3	1,707,313
	rank	1	9	7	11	2	4	6	5	3	8	10	12	13
C11-F10	mean	−21.4889	−14.5416	−17.1501	−13.0457	−19.0113	−14.9125	−13.5714	−15.1881	−14.6597	−12.1691	−13.8193	−12.2573	−11.996
	best	−21.8299	−15.5735	−17.3545	−13.4342	−19.4012	−18.8263	−14.1193	−20.9515	−15.1354	−12.2888	−14.3188	−12.3375	−12.0839
	worst	−20.7878	−14.0389	−16.7705	−12.8424	−18.6173	−12.8716	−13.1807	−12.2848	−13.5969	−12.0396	−13.0951	−12.1735	−11.8831
	std	0.518028	0.764494	0.297295	0.297232	0.43758	2.932237	0.430196	4.264594	0.7905	0.126608	0.657859	0.080422	0.094638
	median	−21.669	−14.277	−17.2377	−12.9532	−19.0134	−13.976	−13.4928	−13.7581	−14.9532	−12.1739	−13.9316	−12.2591	−12.0085
	rank	1	7	3	10	2	5	9	4	6	12	8	11	13
C11-F11	mean	571,712.3	5,328,958	1,075,534	8,031,460	1,666,015	5,454,829	1,273,865	1,355,976	3,588,035	4,805,107	1,446,911	4,814,899	5,612,143
	best	260,837.9	5,076,905	873,531.3	7,760,848	1,551,469	4,560,614	1,162,788	748,605.8	3,403,937	4,770,608	1,304,077	4,785,378	5,565,110
	worst	828,560.9	5,661,658	1,250,006	8,214,168	1,800,123	6,560,319	1,431,883	2,601,629	3,895,613	4,841,240	1,610,216	4,842,787	5,662,076
	std	271,080	298,039	180,691.1	209,754.4	130,844.8	901,463.8	125,636.6	918,912.5	233,276	35,734.2	137,640.7	34,263.64	45,115.52
	median	598,725.2	5,288,634	1,089,299	8,075,412	1,656,234	5,349,191	1,250,395	1,036,835	3,526,294	4,804,290	1,436,677	4,815,717	5,610,694
	rank	1	10	2	13	6	11	3	4	7	8	5	9	12
C11-F12	mean	1,199,805	7,565,001	3,131,984	11,847,407	1,275,035	4,593,958	5,288,069	1,321,780	1,407,168	12,813,481	5,265,826	2,193,624	12,955,713
	best	1,155,937	7,249,457	3,037,655	11,005,819	1,199,042	4,365,138	4,921,550	1,191,166	1,253,032	12,079,057	5,013,496	2,052,641	12,839,240
	worst	1,249,353	7,846,572	3,201,406	12,588,956	1,354,436	4,711,793	5,463,430	1,445,492	1,539,003	13,381,134	5,440,440	2,374,365	13,075,086
	std	48,993.46	269,902.4	77,934.33	709,107.4	74,270.29	176,865.1	274,490.1	114,079	129,661.2	596,428.5	201,014.3	145,668.3	105,930.4
	median	1,196,965	7,581,987	3,144,437	11,897,426	1,273,331	4,649,451	5,383,648	1,325,231	1,418,318	12,896,866	5,304,684	2,173,745	12,954,263
	rank	1	10	6	11	2	7	9	3	4	12	8	5	13
C11-F13	mean	15,444.2	15,812.19	15,449.88	16,212.57	15,463.3	15,487.2	15,527.25	15,502.86	15,496.86	15,879.01	115,295.8	15,487.77	28,322.83
	best	15,444.19	15,647.84	15,448.97	15,844.33	15,460.98	15,478.15	15,489.1	15,485.46	15,490.45	15,608.37	83,844.39	15,472.25	15,460.73
	worst	15,444.21	16,210.14	15,450.58	17,127.49	15,467.32	15,498.33	15,579.17	15,536.74	15,507.94	16,377.02	157,954.9	15,520.56	66,597.52
	std	0.009445	292.5405	0.736869	671.5627	3.070858	10.66753	45.92712	26.08555	8.466667	379.7534	36,436.54	24.13568	27,864.62
	median	15,444.2	15,695.38	15,449.99	15,939.23	15,462.46	15,486.17	15,520.37	15,494.63	15,494.52	15,765.32	109,691.9	15,479.13	15,616.53
	rank	1	9	2	11	3	4	8	7	6	10	13	5	12
C11-F14	mean	18,295.35	101,653.8	18,531.86	204,818.7	18,610.08	19,427.05	19,156.72	19,328.03	19,162.86	277,104.2	19,039.05	19,067.85	19,056.56
	best	18,241.58	77,844.33	18,433.29	151,402.6	18,525.09	19,197.39	19,010.34	19,228.27	19,023.04	28,919.66	18,780.81	18,920.4	18,802.38
	worst	18,388.08	141,318.6	18,627.71	294,218.2	18,686.26	19,915.98	19,266.53	19,403.53	19,330.36	532,867	19,214.64	19,206.21	19,324.98
	std	74.38679	31,002.63	100.4132	69,857.14	75.5763	359.6829	130.2265	82.13068	148.4293	264,198.4	207.9666	127.972	233.6991
	median	18,275.87	93,726.14	18,533.22	186,827.1	18,614.48	19,297.4	19,175	19,340.15	19,149.02	273,315.1	19,080.37	19,072.4	19,049.45
	rank	1	11	2	12	3	10	7	9	8	13	4	6	5
C11-F15	mean	32,883.58	807,662.7	99,246.56	1,698,853	32,948.86	52,073.78	197,302.8	33,083.1	33,063.51	13,654,593	269,150.9	33,249.55	7,029,271
	best	32,782.17	335,111.3	42,006.57	712,650.2	32,870.55	33,044.86	32,994.05	32,999.45	33,025.79	2,864,102	238,633.6	33,238.56	3,201,535
	worst	32,956.46	2,025,189	163,300.4	4,428,557	33,019.16	108,933.8	280,775.5	33,136.06	33,129.28	20,360,257	290,038.3	33,267.85	12,044,118
	std	79.94256	889,583.9	71,193.56	1,990,353	66.4763	41,394.96	122,156.8	65.47578	51.66162	8,687,568	26,115.39	14.21884	4,427,529
	median	32,897.86	435,175.2	95,839.63	827,102.2	32,952.87	33,158.25	237,720.8	33,098.45	33,049.48	15,697,006	273,965.9	33,245.9	6,435,715
	rank	1	10	7	11	2	6	8	4	3	13	9	5	12
C11-F16	mean	133,550	852,515.9	135,494.4	1,741,395	137,689.9	144,300.8	141,678.2	141,363.5	144,961.1	78,713,310	16,589,469	70,453,795	67,648,001
	best	131,374.2	271,555.2	133,906.1	435,162.3	135,606.9	141,665.4	136,481.6	133,721.9	142,697.5	76,704,271	8,433,558	58,281,367	54,676,847
	worst	136,310.8	1,992,858	136,234.8	4,304,701	141,382.2	145,887.3	146,823.4	148,906.5	150,289.6	80,979,120	30,001,092	84,187,611	86,522,477
	std	2485.329	845,493.3	1168.748	1,900,510	2815.212	2202.366	4704.048	6908.098	3912.739	1,956,382	10,183,919	12,193,902	14,772,336
	median	133,257.5	572,825.3	135,918.4	1,112,859	136,885.2	144,825.3	141,703.9	141,412.9	143,428.7	78,584,925	13,961,613	69,673,101	64,696,339
	rank	1	8	2	9	3	6	5	4	7	13	10	12	11
C11-F17	mean	1,926,615	7.93E+09	2.05E+09	1.37E+10	2,293,861	1.13E+09	8.58E+09	3,020,765	2,938,888	1.98E+10	9.93E+09	1.84E+10	1.94E+10
	best	1,916,953	6.76E+09	1.86E+09	9.87E+09	1,957,675	9.36E+08	6.12E+09	2,291,610	2,029,200	1.9E+10	8.73E+09	1.63E+10	1.81E+10
	worst	1,942,685	8.8E+09	2.24E+09	1.68E+10	2,915,059	1.3E+09	1.14E+10	3,551,652	4,662,207	2.06E+10	1.05E+10	2.13E+10	2.19E+10
	std	12,470.83	9.84E+08	1.83E+08	3.25E+09	468,908.4	2.03E+08	2.43E+09	620,050.3	1,295,060	7.28E+08	8.83E+08	2.49E+09	1.87E+09
	median	1,923,412	8.09E+09	2.05E+09	1.41E+10	2,151,356	1.15E+09	8.4E+09	3,119,900	2,532,073	1.97E+10	1.02E+10	1.81E+10	1.87E+10
	rank	1	7	6	10	2	5	8	4	3	13	9	11	12
C11-F18	mean	942,057.5	48,788,595	5,915,000	1.05E+08	971,984.9	1,926,661	8,592,280	986,474.2	1,024,549	27,556,574	9,963,062	1.2E+08	1.02E+08
	best	938,416.2	33,587,280	3,598,584	72,496,984	949,866.2	1,696,441	3,757,481	972,178.6	965,175.1	21,861,781	7,461,469	1E+08	97,794,840
	worst	944,706.9	55,479,310	10,072,337	1.2E+08	1,030,674	2,227,417	15,006,887	993,724.5	1,181,666	29,806,868	12,546,238	1.33E+08	1.05E+08
	std	2882.138	11,196,197	3,292,143	24,174,063	42,861.31	277,004.9	5,187,569	10,717.17	114,623.8	4,163,281	2,480,176	15,806,321	3,328,144
	median	942,553.5	53,043,894	4,994,539	1.14E+08	953,699.6	1,891,392	7,802,376	989,996.7	975,677.3	29,278,824	9,922,270	1.23E+08	1.02E+08
	rank	1	10	6	12	2	5	7	3	4	9	8	13	11
C11-F19	mean	1,025,341	48,045,429	6,024,330	1.03E+08	1,138,731	2,312,239	9,177,497	1,438,696	1,337,888	31,640,166	5,679,788	1.53E+08	1.02E+08
	best	967,927.7	41,007,057	5,529,035	88,789,545	1,068,540	2,091,831	1,949,236	1,124,620	1,214,014	22,188,480	2,262,615	1.39E+08	99,335,591
	worst	1,167,142	61,046,693	7,266,048	1.29E+08	1,293,909	2,700,255	16,514,231	1,862,140	1,516,560	39,430,521	7,413,240	1.77E+08	1.05E+08
	std	103,555.5	9,868,988	909,126.9	20,540,250	114,017.5	291,364.9	7,492,785	336,266.6	139,372.1	8,153,343	2,551,795	18,022,451	2,504,883
	median	983,146.6	45,063,983	5,651,119	96,595,837	1,096,237	2,228,435	9,123,261	1,384,012	1,310,489	32,470,832	6,521,648	1.48E+08	1.02E+08
	rank	1	10	7	12	2	5	8	4	3	9	6	13	11
C11-F20	mean	941,250.4	51,056,364	5,326,862	1.11E+08	960,500.6	1,727,330	6,556,181	971,537.3	994,311.4	30,716,418	12,744,216	1.41E+08	1.02E+08
	best	936,143.2	44,933,180	4,709,950	97,107,724	957,168.3	1,564,565	6,183,561	962,742.4	975,262.1	30,044,795	8,502,443	1.29E+08	97,262,940
	worst	946,866.6	60,440,462	5,986,883	1.32E+08	962,608	1,997,607	7,053,710	981,674.9	1,009,090	31,442,402	19,667,237	1.53E+08	1.06E+08
	std	5208.733	7,215,894	578,753.3	16,195,092	2558.588	224,547	406,329.4	9206.801	15,914.84	634,789.9	5,328,269	14,753,402	3,998,704
	median	940,995.9	49,425,907	5,305,307	1.07E+08	961,113	1,673,574	6,493,726	970,866	996,446.5	30,689,238	11,403,593	1.41E+08	1.03E+08
	rank	1	10	6	12	2	5	7	3	4	9	8	13	11
C11-F21	mean	12.71443	46.34686	21.01723	69.56401	15.96249	28.26275	36.20316	26.2373	21.66735	91.01918	37.89568	95.47795	92.70667
	best	9.974206	38.73143	19.57825	52.49434	13.78985	25.13662	33.54273	23.43905	19.92905	44.78771	33.83682	82.90208	54.02514
	worst	14.97499	54.54122	22.89874	86.63233	18.25234	29.53126	39.5914	29.18722	24.00863	133.0501	40.61476	105.7638	112.5417
	std	2.506594	7.425814	1.558182	16.45118	2.263924	2.29002	2.912469	3.413229	2.014941	39.49753	3.257039	12.46174	29.75296
	median	12.95425	46.0574	20.79597	69.5647	15.90388	29.19156	35.83926	26.16147	21.36585	93.11946	38.56558	96.62294	102.1299
	rank	1	9	3	10	2	6	7	5	4	11	8	13	12
C11-F22	mean	5.920103	16.74641	12.47935	20.60699	7.613273	17.40635	12.74259	13.42229	10.58714	17.43517	20.35526	16.99013	21.88339
	best	2E−10	13.86501	8.478707	18.18457	0.380987	16.50584	7.451926	11.38898	1.050147	16.64263	17.71226	10.14258	20.69375
	worst	12.30606	19.6453	16.37007	23.21547	12.69538	19.05236	16.77422	15.48484	16.38551	18.69533	22.1194	23.16349	24.33594
	std	7.476538	3.058894	4.906722	2.611096	6.161367	1.264455	4.734219	2.406333	7.278318	1.063321	2.051999	6.277694	1.854985
	median	5.687176	16.73766	12.53432	20.51396	8.688362	17.03359	13.37211	13.40768	12.45645	17.20137	20.79468	17.32722	21.25194
	rank	1	7	4	12	2	9	5	6	3	10	11	8	13
Sum rank		22	191	109	231	55	146	145	118	97	222	157	198	224
Mean rank		1	8.681818	4.954545	10.5	2.5	6.636364	6.590909	5.363636	4.409091	10.09091	7.136364	9	10.18182
Total rank		1	2	12	4	13	3	11	9	6	7	10	5	8
Wilcoxon: *p-*value			1.58E−15	9.00E−15	1.58E−15	6.54E−15	3.37E−15	1.58E−15	3.68E−12	6.54E−15	4.94E−15	7.85E−15	2.34E−15	4.94E−15

**Table 7:** Performance of optimization algorithms on pressure vessel design problem.

Algorithm	Optimum Variables				Optimum Cost
	*T_s_*	*T_h_*	*R*	*L*	
GAO	0.7780271	0.3845792	40.312284	200	5882.8955
WSO	0.7780269	0.3845797	40.312282	200	5882.9013
AVOA	0.7780308	0.384581	40.312476	199.99732	5882.9077
RSA	1.1950157	0.64038	60.549321	48.031984	7759.8234
MPA	0.7780271	0.3845792	40.312284	200	5882.9013
TSA	0.7794994	0.385819	40.386517	200	5909.3749
WOA	0.911517	0.4510723	46.230782	133.83941	6270.8621
MVO	0.8344267	0.4164052	43.217775	163.90679	6003.8497
GWO	0.7784599	0.3858127	40.320627	199.96442	5890.2105
TLBO	1.5622593	0.4813024	47.695987	124.64823	10,807.366
GSA	1.1300127	1.1576349	44.110061	190.7876	11,984.417
PSO	1.55006	0.6231249	63.139483	49.78495	9998.6395
GA	1.406417	0.7832762	58.253368	73.964478	10,920.286

**Table 8:** Statistical results of optimization algorithms on pressure vessel design problem.

Algorithm	Mean	Best	Worst	Std	Median	Rank
GAO	5882.8955	5882.8955	5882.8955	1.87E−12	5882.8955	1
WSO	5891.226	5882.9013	5965.0365	22.218932	5882.9017	3
AVOA	6219.5386	5882.9077	7046.3206	352.35848	6047.6955	5
RSA	12,409.586	7759.8234	19,991.769	3127.065	11,403.338	9
MPA	5882.9013	5882.9013	5882.9013	3.68E−06	5882.9013	2
TSA	6271.132	5909.3749	6948.3792	333.1584	6143.6153	6
WOA	7998.6372	6270.8621	12,805.388	1681.8974	7579.6333	8
MVO	6518.1019	6003.8497	7050.4059	320.31898	6572.19	7
GWO	6012.3675	5890.2105	6670.9945	239.38549	5898.5494	4
TLBO	28,273.334	10,807.366	60,311.64	13,795.65	24,975.491	12
GSA	20,643.589	11,984.417	32,105.445	6711.6675	19,830.394	10
PSO	29,687.575	9998.6395	50,712.307	12,915.318	32,709.339	13
GA	25,427.766	10,920.286	45,530.922	10,828.815	22,551.255	11

**Table 9:** Performance of optimization algorithms on speed reducer design problem.

Algorithm	Optimum Variables							Optimum Cost
	b	*M*	*p*	*l*_1_	*l*_2_	*d*_1_	*d*_2_	
GAO	3.5	0.7	17	7.3	7.8	3.3502147	5.2866832	2996.3482
WSO	3.5000004	0.7	17	7.3000087	7.8000004	3.3502148	5.2866833	2996.3483
AVOA	3.5	0.7	17	7.3000007	7.8	3.3502147	5.2866832	2996.3482
RSA	3.5812009	0.7	17	8.1120092	8.2060046	3.3550151	5.4598984	3160.6387
MPA	3.5	0.7	17	7.3	7.8	3.3502147	5.2866832	2996.3482
TSA	3.5113634	0.7	17	7.3	8.2060046	3.3505018	5.2897957	3011.7899
WOA	3.5770618	0.7	17	7.3	7.9844181	3.3602552	5.2867471	3033.2634
MVO	3.5019838	0.7	17	7.3	8.0370241	3.3672881	5.2868582	3006.8197
GWO	3.5005649	0.7	17	7.3045311	7.8	3.3623131	5.2885569	3000.8991
TLBO	3.549421	0.7035216	25.214085	8.0060043	8.1041201	3.6261576	5.3330891	4999.6584
GSA	3.5201835	0.7024256	17.325213	7.7585847	7.8789461	3.4018063	5.374124	3149.0914
PSO	3.5072099	0.7000634	17.965269	7.3872523	7.8599345	3.566267	5.3372001	3266.1003
GA	3.5687302	0.7049031	17.716975	7.6899131	7.8491985	3.65975	5.3392351	3305.1452

**Table 10:** Statistical results of optimization algorithms on speed reducer design problem.

Algorithm	Mean	Best	Worst	Std	Median	Rank
GAO	2996.3482	2996.3482	2996.3482	9.33E−13	2996.3482	1
WSO	2996.5979	2996.3483	2998.5076	0.515253	2996.3624	3
AVOA	3000.3195	2996.3482	3009.3227	3.495813	3000.2318	4
RSA	3243.4118	3160.6387	3294.782	50.671452	3256.5192	9
MPA	2996.3482	2996.3482	2996.3482	2.81E−06	2996.3482	2
TSA	3027.8743	3011.7899	3039.9713	8.9331108	3029.4495	7
WOA	3131.7628	3033.2634	3391.7111	93.64881	3102.3919	8
MVO	3025.8394	3006.8197	3061.3925	11.679823	3026.2269	6
GWO	3003.637	3000.8991	3008.8921	2.2089208	3003.1807	5
TLBO	6.128E+13	4999.6584	4.435E+14	1.02E+14	2.4E+13	12
GSA	3400.1113	3149.0914	3947.2386	231.00301	3285.8903	10
PSO	9.044E+13	3266.1003	4.581E+14	1.092E+14	6.468E+13	13
GA	4.354E+13	3305.145	2.81E+14	6.859E+13	1.745E+13	11

**Table 11:** Performance of optimization algorithms on welded beam design problem.

Algorithm	Optimum Variables				Optimum Cost
	*h*	*l*	*t*	*b*	
GAO	0.2057296	3.4704887	9.0366239	0.2057296	1.7246798
WSO	0.2057296	3.4704888	9.0366238	0.2057296	1.7248523
AVOA	0.205056	3.4850976	9.0365299	0.2057339	1.7257923
RSA	0.1977725	3.5270192	9.8188942	0.2163579	1.9455461
MPA	0.2057296	3.4704887	9.0366239	0.2057296	1.7248523
TSA	0.2043787	3.4924081	9.0609	0.2061055	1.7327713
WOA	0.2127738	3.3465331	8.9813136	0.2191754	1.8098061
MVO	0.2059617	3.465488	9.0437236	0.2060167	1.7279454
GWO	0.2056085	3.4732685	9.0362859	0.2057905	1.7254435
TLBO	0.3021777	4.3080233	7.0649912	0.3989007	2.86852
GSA	0.2833168	2.8111202	7.6140935	0.2957385	2.0415263
PSO	0.3526171	3.4301508	7.5466754	0.5299732	3.7483578
GA	0.2220901	6.5032092	7.9154768	0.2925869	2.6371953

**Table 12:** Statistical results of optimization algorithms on welded beam design problem.

Algorithm	Mean	Best	Worst	Std	Median	Rank
GAO	1.7246798	1.7246798	1.7246798	2.28E−16	1.7246798	1
WSO	1.7248526	1.7248523	1.7248572	1.101E−06	1.7248523	3
AVOA	1.7568691	1.7257923	1.8286017	0.0321107	1.7446373	7
RSA	2.127039	1.9455461	2.4331287	0.1269176	2.1049933	8
MPA	1.7248523	1.7248523	1.7248523	2.95E−09	1.7248523	2
TSA	1.7409627	1.7327713	1.7490567	0.0049359	1.7410475	6
WOA	2.2407055	1.8098061	3.7687515	0.5650313	2.0426429	9
MVO	1.7392665	1.7279454	1.7690502	0.0121131	1.7356826	5
GWO	1.7269657	1.7254435	1.7305264	0.0011999	1.7267498	4
TLBO	2.929E+13	2.86852	2.826E+14	7.143E+13	5.2174609	12
GSA	2.3577996	2.0415263	2.6295	0.1686298	2.3838126	10
PSO	4.039E+13	3.7483578	2.445E+14	7.713E+13	6.1318573	13
GA	9.913E+12	2.6371953	1.073E+14	3.043E+13	5.1880494	11

**Table 13:** Performance of optimization algorithms on tension/compression spring design problem.

Algorithm	Optimum Variables			Optimum Cost
	*d*	*D*	*p*	
GAO	0.0516891	0.3567177	11.288966	0.0126019
WSO	0.0516873	0.3566758	11.291426	0.0126652
AVOA	0.0512511	0.3462947	11.933872	0.0126696
RSA	0.0503175	0.3192513	14.30236	0.0130991
MPA	0.0516905	0.3567534	11.286873	0.0126652
TSA	0.0510725	0.3420852	12.221253	0.01268
WOA	0.0512287	0.3457669	11.968427	0.01267
MVO	0.0503175	0.3243542	13.574804	0.0127396
GWO	0.0519242	0.3623905	10.968951	0.01267
TLBO	0.0658133	0.8277549	3.7462401	0.0169026
GSA	0.0547015	0.4310298	8.235468	0.0130247
PSO	0.0657408	0.8250149	3.7462402	0.0168129
GA	0.0662247	0.83462	3.7462402	0.0172493

**Table 14:** Statistical results of optimization algorithms on tension/compression spring design problem.

Algorithm	Mean	Best	Worst	Std	Median	Rank
GAO	0.0126019	0.0126019	0.0126019	6.88E−18	0.0126019	1
WSO	0.0126749	0.0126652	0.012805	3.115E−05	0.0126656	3
AVOA	0.013254	0.0126696	0.0139577	0.0004844	0.0131947	8
RSA	0.0131701	0.0130991	0.0132952	6.028E−05	0.0131518	6
MPA	0.0126652	0.0126652	0.0126652	2.47E−09	0.0126652	2
TSA	0.0129235	0.01268	0.0134133	0.0002099	0.0128595	5
WOA	0.0131928	0.01267	0.0142585	0.000525	0.0130205	7
MVO	0.0159749	0.0127396	0.0172239	0.0014311	0.0167707	9
GWO	0.0127154	0.01267	0.0129094	4.805E−05	0.0127132	4
TLBO	0.0173644	0.0169026	0.0178919	0.000311	0.017326	10
GSA	0.0185374	0.0130247	0.0295218	0.0037009	0.0181672	11
PSO	1.818E+13	0.0168129	3.225E+14	7.217E+13	0.0168129	13
GA	1.42E+12	0.0172493	1.469E+13	4.24E+12	0.0238647	12

## Data Availability

Data are contained within the article.

## Appendix A

**Table A1:** Control parameters values of competitor metaheuristic algorithms.

Algorithm	Parameter	Value
GA		
	Type	Real coded
	Selection	Roulette wheel (Proportionate)
	Crossover	Whole arithmetic (Probability = 0.8, *α* ∈ [−0.5, 1.5]
	Mutation	Gaussian (Probability = 0.05)
PSO		
	Topology	Fully connected
	Cognitive and social constant	(*C*_1_, *C*_2_) = (2, 2)
	Inertia weight	Linear reduction from 0.9 to 0.1
	Velocity limit	10% of dimension range
GSA		
	Alpha, *G*_0_, *R_norm_*, *R_power_*	20, 100, 2, 1
TLBO		
	*T_F_*: teaching factor	*T_F_* = round [(1 + *rand*)]
	random number	*rand* is a random number between [0–1]
GWO		
	Convergence parameter (*a*)	*a*: Linear reduction from 2 to 0.
MVO		
	wormhole existence probability (WEP)	Min(WEP) = 0.2 and Max(WEP) = 1.
	Exploitation accuracy over the iterations (*p*)	*p* = 6.
WOA		
	Convergence parameter (*a*)	*a*: Linear reduction from 2 to 0.
	*r* is a random vector in [0–1]	
	*l* is a random number in [−1, 1]	
TSA		
	P_min_ and P_max_	1, 4
	*c*1, *c*2, *c*3	random numbers lie in the range of [0–1]
MPA		
	Constant number	*P* = 0.5
	Random vector	*R* is a vector of uniform random numbers in [0, 1]
	Fish Aggregating Devices (*FADs*)	*FADs* = 0.2
	Binary vector	*U* = 0 or 1
RSA		
	Sensitive parameter	*β =* 0.01
	Sensitive parameter	*α* = 0.1
	Evolutionary Sense (ES)	ES: randomly decreasing values between 2 and −2
AVOA		
	L_1_, L_2_	0.8, 0.2
	w	2.5
	P_1_, P_2_, P_3_	0.6, 0.4, 0.6
WSO		
	F_min_ and F_max_	0.07, 0.75
	*τ*, *a*_o_, *a*_1_, *a*_2_	4.125, 6.25, 100, 0.0005
